# Liquid Biopsy Biomarkers in Metastatic Castration-Resistant Prostate Cancer Treated with Second-Generation Antiandrogens: Ready for Clinical Practice? A Systematic Review

**DOI:** 10.3390/cancers17152482

**Published:** 2025-07-27

**Authors:** Andrei-Vlad Badulescu, Razvan Rahota, Alon Vigdorovits, Ovidiu Laurean Pop

**Affiliations:** 1 Doctoral School of Biomedical Sciences, University of Oradea, 410087 Oradea, Romania; alonvigdorovits@gmail.com; 2 Department of Morphological Sciences, Faculty of Medicine and Pharmacy, University of Oradea, 410073 Oradea, Romania; drovipop@gmail.com; 3 Department of Pathology, Bihor County Clinical Emergency Hospital, 410169 Oradea, Romania

**Keywords:** metastatic castration-resistant prostate cancer, liquid biopsy, cell-free DNA, circulating tumor cells, extracellular vesicles, circulating RNA

## Abstract

**Background**: Second-generation androgen receptor signaling inhibitors are one of the main treatment options in metastatic castration-resistant prostate cancer (mCRPC). Nonetheless, a considerable proportion show limited response to treatment, which indicates the need for convenient, easily accessible predictor biomarkers, a role suited for liquid biopsy. **Methods**: We conducted a PRISMA-compliant systematic review of four databases (Embase, Medline, Scopus, Web of Science) to identify all studies (observational studies and clinical trials) investigating cell-free DNA, circulating tumor cells, exosomes, and circulating RNAs as prognostic markers in metastatic castration-resistant patients starting androgen receptor signaling inhibitors. We excluded studies that evaluated combination therapies, rare histological subtypes or included nonmetastatic or castrate-sensitive disease. We also evaluated whether published papers followed reporting guidelines (REMARK, STROBE, or CONSORT for abstracts). **Results**: We identified a total of 123 reports, from which we identified only a few well-studied and consistent biomarkers: androgen receptor overexpression/copy number gain and splice variant 7, as well as disease burden markers (circulating tumor DNA fraction and circulating tumor cell concentration). Alterations or copy number loss in tumor suppressors PTEN, RB1, and TP53 were second in terms of quantity and consistency of evidence. However, a large majority of identified biomarkers were relatively understudied or inconsistent. We identified two potential vulnerabilities: inconsistent adherence to reporting guidelines and the under-inclusion of patients of non-Western European ancestry. **Conclusions**: A large number of biomarkers were linked to worse outcomes in prostate cancer; nonetheless, in most cases, the evidence is limited or inconsistent, or even contradictory. The main exceptions pertain to androgen receptor signaling and disease burden, and, to a smaller extent, to certain tumor suppressor genes. Further studies are needed to confirm their clinical utility, using clear and consistent methodologies and including patients from currently understudied populations.

## Simple Summary

Liquid biopsy techniques (circulating and exosomal nucleic acids and circulating tumor cells) represent a convenient method to study the molecular landscape of metastatic castration-resistant prostate cancer. Our systematic review attempted to identify the most promising and best-characterized liquid biopsy biomarkers. We concluded that, despite the large number of studies, relatively few liquid biopsy markers have been studied in sufficient depth and with sufficiently consistent results: nonspecific markers, such as circulating tumor DNA fraction, circulating tumor cell count, as well as androgen receptor splice variant 7 and androgen receptor overexpression and amplification, and potentially also three tumor suppressor genes, namely, PTEN, RB1, and TP53. This highlights the need for more in-depth studies of potentially promising biomarkers, ideally in large and diverse populations.

## 1. Introduction

Prostate cancer (PCa) is the second most common cancer in men, with almost 1.5 million new cases (14.2% of all new cancer cases) worldwide in 2022, as well as the fifth cancer by mortality (with almost 397,000 deaths, or 7.3% of all cancer deaths in men), according to the GLOBOCAN estimates of the International Agency for Research on Cancer [1]. Most commonly, PCa presents initially as a hormone-dependent disease, which can be managed with androgen deprivation therapy (ADT) through gonadal suppression or ablation; nonetheless, resistance to simple androgen deprivation and dissemination eventually occurs, leading to metastatic castration-resistant prostate cancer (mCRPC) [2]. It should be noted that androgen receptor (AR) signaling remains relevant even in this setting, and androgen receptor signaling inhibitors (ARSIs) are, alongside chemotherapy (ChT) with the two taxanes, docetaxel (DTX) and cabazitaxel (CBZ), and poly (ADP-ribose) polymerase inhibitors (PARPis), the main therapeutic options available in this stage [3].

Two approaches to targeting the AR axis in mCRPC are currently approved: inhibiting steroidogenesis and targeting the AR directly. The former, originally represented by ketoconazole, a weak inhibitor of adrenal steroidogenesis, is presently achieved with abiraterone acetate (ABI), an inhibitor of cytochrome P450 17A1 (CYP17A1), an enzyme with 17,20-lyase and 17α-hydroxylase activity responsible for androgen synthesis in the testis and adrenal cortex [4,5]; subsequently, ABI decreases the availability of dehydroepiandrosterone and androstenedione, which are converted to testosterone or, respectively, dihydrotestosterone (via an intermediate 5α-androstanedione) by the malignant cells [6]. On the other hand, targeting the AR in mCRPC is achieved using the second-generation antiandrogens enzalutamide (ENZ), apalutamide (APA), or darolutamide, which act as AR antagonists, as well as inhibiting its translocation to the nucleus and its binding to androgen response elements. APA and darolutamide have essentially similar mechanisms of action, improved toxicity profiles, and (particularly darolutamide) retain their efficacy in the context of certain AR point mutations that confer resistance to ENZ [7,8,9].

**Figure 1:**
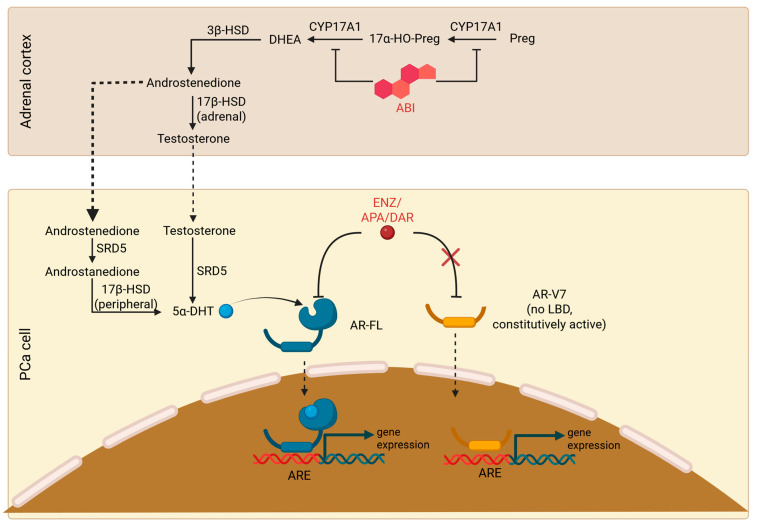
The mechanism of action of available ARSI and ARSI resistance in AR splice variant 7. Based on the studies of [6,12]. Created in BioRender. Badulescu, A. (2025) https://BioRender.com/m0fjgdu. Abbreviations: ABI, abiraterone; APA, apalutamide; ARE, androgen response element; AR-FL, full-length AR; AR-V7, AR splice variant 7; DHEA, dehydroepiandrosterone; DHT, dihydrotestosterone; HSD, hydroxysteroid dehydrogenase; LBD, ligand-binding domain; Preg, pregnenolone; and SRD5, steroid reductase 5 (5α-reductase).

Despite their different mechanisms of action, cross-resistance between ABI and AR antagonists is common and mediated through multiple and incompletely understood pathways: the best known is the emergence of AR splice variant-expressing mCRPC, of which the most common is AR-V7, an AR variant lacking the ligand-binding domain. The exact mechanisms of AR-V7 signaling are unclear, and heterodimerization with full-length AR (AR-FL) is believed to be responsible for androgen signaling [10,11]. In a seminal study by Antonarakis et al., the detection of AR-V7 on circulating tumor cells (CTCs) was associated with dismal responses to both ENZ and ABI: a 0% prostate-specific antigen (PSA) response rate and a median PSA progression-free survival (PSA-PFS) of little over 1 month in both groups, with a hazard ratio for PSA progression of 7.4 in the ENZ cohort and 16.1 in the ABI cohort [12]. Figure 1 provides an overview of the mechanism of action of currently available ARSI (ABI and, respectively, ENZ/APA/darolutamide) in comparison to AR-V7.

Nonetheless, AR splice variants are not the only determinant of response to ARSIs: in the above-mentioned study, PSA response rates were only 53% and 68% in the ENZ and ABI cohorts, respectively [12]. Moreover, a meta-analysis of the impact of AR-V7 expression in liquid biopsies, either CTCs or cell-free DNA (cfDNA), while supporting the significant association between AR-V7 expression and poor PFS and overall survival (OS) with ARSI treatment, does show a certain heterogeneity, further highlighting the relevance of other biomarkers beyond AR-V7. Another significant observation of the same meta-analysis is that AR-V7 expression has a lower impact on PFS and OS under ChT, an argument for the use of AR-V7 in guiding the choice of treatment.

Liquid biopsy refers to the molecular testing of biological fluids (most commonly blood but occasionally urine, serosal, cerebrospinal, or seminal fluid). Circulating tumor cells, cell-free DNA (of particular relevance being the circulating tumor DNA fraction thereof), and exosomes (and especially their content, mainly miRNA species but also proteins or metabolites) are the best studied liquid biopsy markers; other markers, such as tumor-educated platelets and various circulating RNA species in plasma or whole blood, have also shown potential.

PCa represents an ideal context for employing liquid biopsies for multiple reasons. On the one hand, its evolution from gonadal androgen dependence to castration resistance and then from ARSI-sensitivity to ARSI refractoriness strongly supports a rapid, repeatable, and minimally invasive approach, as is the case with blood-based liquid biopsy techniques. Taking into account the fact that PCa patients tend to be elderly and frail [13], the existence of an alternative to the invasiveness of a metastatic site biopsy can be particularly beneficial. Likewise, the long course of the disease inevitably leads to considerable tumor heterogeneity, which can be lost when performing conventional biopsies on metastatic sites; not least, urine liquid biopsy has shown some utility as a screening tool, allowing patients with low-grade, clinically insignificant cancer to avoid ultrasound-guided prostate biopsy [14,15,16,17].

Nonetheless, the wider adoption of liquid biopsy in clinical practice is relatively slow. Specifically, in the last (1.2025) version of the National Comprehensive Cancer Network Clinical Practice Guidelines in Oncology (NCCN^®^ Guidelines), the pretreatment workup still overwhelmingly consists of metastasis biopsy and tissue biomarker evaluation: cfDNA testing is only recommended for homologous recombination repair (HRR) deficiency prior to rucaparib, whereas AR-V7 testing in CTCs prior to commencing ARSI only has a weak recommendation [18]. This is explained by a series of potential limitations of liquid biopsy techniques, such as the lack of standardization of both the experimental and data analysis protocols, risk of sample contamination, an intrinsically low sensitivity due to small amounts of the circulating biomarker, interference from clonal hematopoiesis, and finally a lack of sufficient clinical and real-world evidence [19,20].

In order to summarize the current progress on the identification of liquid biopsy biomarkers for antiandrogen response in mCRPC, we performed a systematic review of studies of well-known (such as AR-V7) and emerging liquid biopsy markers in predicting outcomes under AR axis-targeting agents.

## 2. Materials and Methods

### 2.1. Study Design and Literature Search

Our study was conducted in accordance with the PRISMA 2020 statement [21] and aimed to retrieve studies published up until 1 December 2024, in the following databases: Embase, Medline, Scopus, and Web of Science. The search queries are presented in Supplementary Materials Data S1. Summary data for the identified records were exported as comma-separated value files; the four files were then merged in a spreadsheet application, which was then used for duplicate removal. Full texts were retrieved using institutional access provided by the University of Oradea; when we were unable to retrieve certain articles, we attempted to contact the authors. Two authors screened the titles and abstracts of identified studies; in a second step, the two reviewers evaluated the full texts for eligibility. Disagreements were resolved through discussion.

We were primarily interested in the following outcome variables: hazard ratios for (clinical, radiographic, PSA or unspecified) progression-free survival (PFS) and overall survival (OS) according to the univariate Cox regression model, as well as their 95% confidence intervals; if these were not reported, other data, namely, multivariate Cox hazard ratios, median survival, and *p*-values for the log-rank test were collected. For some articles, median survival times were not explicitly stated but were only reported in Kaplan–Meier curves; in this case, we estimated median survival from the graphs geometrically.

Additionally, we collected data for the following variables: the prevalence of each biomarker, whether the biomarker was evaluated in multivariable analyses, the country in which the study was performed, and whether the studies adhered to reporting guidelines.

Due to the expected heterogeneity of the identified studies (a large number of individual biomarkers with few studies per biomarker, various study methodologies, etc.), we did not attempt to perform a statistical synthesis.

### 2.2. Inclusion and Exclusion Criteria

We used the following inclusion criteria to assess eligibility:

- Full-text articles and conference abstracts.
- Observational studies and clinical trials of patients with mCRPC treated with AR axis-targeting agents.
- Studies that used liquid biopsy to obtain the following biomaterials: circulating tumor cells, cell-free nucleic acids, and extracellular vesicles.

Reports were excluded according to the following criteria:

- Articles not in English.
- Specific study types: Case reports and case series, clinical trial protocols, editorials, opinion articles, surveys, in vitro, in silico, and animal-model studies, reviews, and pooled analyses (when the individual studies were already retrieved).
- Other study populations: Studies that grouped both mCRPC and nonmetastatic or castrate-sensitive PCa, studies of specific subtypes of mCRPC (i.e., neuroendocrine, small-cell, aggressive-variant PCa).
- Other or unclear treatment (other treatments than AR axis-targeting agents—ChT, PSMA-targeted radionuclide therapy, or combinations of AR axis-targeting agents and other treatments).
- Inappropriate or unclear timing of liquid biopsy (more than one month before or after the beginning of treatment).
- Articles not reporting time-to-event data: PFS/time to progression (clinical, radiographic, PSA, or unspecified) or OS.
- Preliminary results.

### 2.3. Handling of Abstracts

The inclusion of abstracts and the gray literature in systematic reviews is recommended by the Cochrane Handbook, since little more than a third of conference articles actually reach full publication [22,23]. However, it can lead to double counting of studies, and the methodology is difficult to ascertain due to the size constraints of the abstract [24,25]. Nonetheless, due to the relative novelty of the field, we decided to include abstracts as well, in order to identify emerging biomarkers. In this context, two remarks need to be made.

Firstly, preliminary data (typically reported as an abstract) was excluded when newer results (usually a full text, but sometimes another, newer abstract) were available.

Secondly, in the situation when a study consisted of a full text and (at least) one abstract, and the full text had to be excluded due to an unaccepted methodology, we proceeded to exclude the abstract(s) as well, even when double counting would not have been an issue (e.g., the abstract reports data on a different biomarker than the full text). The rationale is that the methodology is the same for all reports of the same study.

### 2.4. Quality Assessment

The reporting quality of full-text reports was evaluated using the REMARK checklist [26] and with the CONSORT [27] (abstracts of clinical trials) and STROBE [28] (abstracts of observational studies) checklists for abstract-only reports. Biomarker studies that used data collected as part of clinical trials were also evaluated with STROBE. The compliance of identified studies to reporting guidelines was evaluated by two authors; disagreements were resolved by discussion. Since the three checklists are primarily guidelines for writing, rather than evaluating articles, there is no recommended threshold for which an article should be considered checklist-compliant. We decided on a 75% threshold to define which articles can be considered to adhere to the checklist (at least 15 of 20 items for REMARK, at least 13 of 17 items for CONSORT, and at least 8 of 11 items for STROBE).

Whether the articles followed the checklists is mentioned in the Section 3 at the first mention of each article (in the text or tables). The full list of articles, including the rating of each item in the checklists, is presented in Supplementary Materials Data S2.

## 3. Results

### 3.1. Search Results and Study Characteristics

We identified 3559 records from four databases (Embase, PubMed, Scopus, and Web of Science) [21]. After removing 1674 duplicate records, we proceeded to screen the remaining 1885 records according to title and abstract. At this stage, we removed 1521 records, leaving us with 364 records for full-text assessment. We were unable to retrieve six reports, even after attempting to contact the authors.

Of the 358 retrieved reports, we excluded a total of 235 reports: 1 study did not have an English-language full text, 5 reports were removed due to study type (one in vitro study, one survey, two case series, and one pooled analysis of two already included studies), 28 studies did not use liquid biopsy/did not report liquid biopsy results, 92 studies did not provide sufficient outcome data (i.e., did not report progression-free survival or overall survival), 5 investigated a different population (i.e., both castration-resistant and castration-sensitive patients or nonmetastatic CRPC), 54 studies investigated other treatments (i.e., either used a different treatment entirely, or used ARSIs in combination with another treatment, or did not report outcomes in the ARSI-only subgroup), and 18 studies had performed liquid biopsy in an inappropriate or unclear timeframe (either the timing was not reported, or the biofluid sampling was performed more than one month before or one month after the beginning of treatment). Finally, we excluded 32 articles that reported preliminary results when newer data was available.

**Figure 2:**
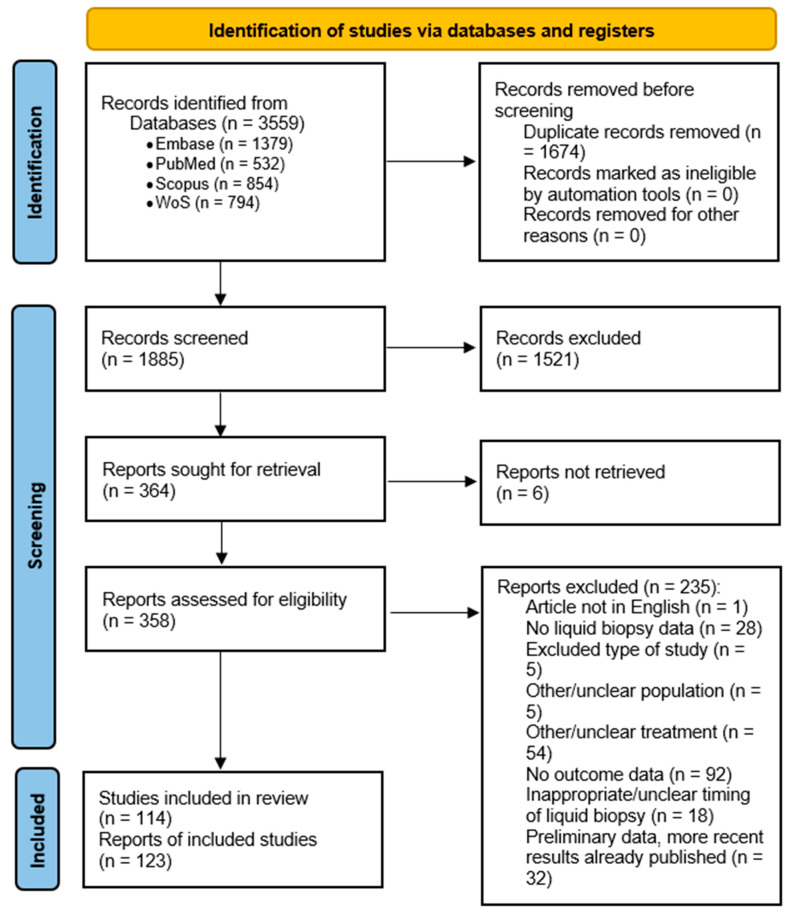
The PRISMA flow diagram.

The exclusions above include six abstracts that were removed after their corresponding full-text articles revealed that the studies had a methodology that did not correspond to the inclusion and exclusion criteria: four for having an excluded or unclear treatment, one because of the timing of the liquid biopsy, and one for not having used liquid biopsy. Finally, we were left with 114 studies, consisting of 123 reports: 88 full-text articles and 35 abstracts. The PRISMA flowchart [21] is presented in Figure 2 below.

With respect to the evaluated biomarkers, we identified 56 reports that investigated CTCs only and 41 that evaluated cfDNA only. Circulating RNA was investigated in 17 studies: 7 for total plasma RNA, 5 for exosomal RNA, and 4 investigated whole-blood RNA. A total of 11 papers evaluated multiple markers; namely, 5 evaluated both CTCs and cfDNA, and there was 1 article for each of the following: cfDNA and plasma RNA, cfDNA and exosomal RNA, CTC and exosomal RNA, CTC and whole-blood RNA, CTC, and extracellular vesicles.

Regarding the treatment that was evaluated in each article, the vast majority of identified papers investigated approved second-generation antiandrogens. Specifically, we found 28 reports investigating ABI alone; 15 studied ENZ alone; 4 articles described separate ABI and ENZ cohorts; 67 articles used ENZ or ABI but did not evaluate them separately; 4 used ENZ, ABI, or APA; and 2 used unspecified (most likely second-generation) antiandrogens. Three reports used combinations of androgen-targeted agents: one paper investigated APA with or without ABI, one ENZ with or without ABI, and one used testosterone plus ABI or ENZ (as bipolar androgen therapy). There were no studies of darolutamide, investigational hormonal agents, or first-generation androgens that matched the inclusion criteria.

The identified biomarkers are presented in the following sections. Since the same report could have contributed more than one result (i.e., the same report investigated an ABI and an ENZ cohort, or two different thresholds for the same biomarker), the number of entries in the tables is typically larger than the number of reports.

### 3.2. Nonspecific Liquid Biopsy Markers

We identified five types of “nonspecific” markers (i.e., not representing a specific molecular alteration) in the included studies: cfDNA concentration, ctDNA fraction, CTC counts, CTC dynamics, CTC nuclear size, and tumor-derived extracellular vesicle (tdEV) counts.

CTC counts are the best studied marker, with 24 reports [29,30,31,32,33,34,35,36,37,38,39,40,41,42,43,44,45,46,47,48,49,50,51,52], followed by ctDNA fraction (12 reports, [53,54,55,56,57,58,59,60,61,62,63,64]), cfDNA concentration (3 reports, [65,66,67]), and CTC dynamics (3 reports, [51,68,69]); finally, CTC nuclear size [70] and tdEV counts [30] were each evaluated in 1 report.

CTC counts were shown to be significantly associated with a worse prognosis across a wide range of assays and thresholds. CTC counts were shown to be significantly associated with worse PFS or OS across different ARSI and prior treatments, as well as different populations. The prevalence of CTC positivity ranged from 22% to 73%; nonetheless, differences between each assay’s sensitivity and between the investigators’ choice of threshold need to be taken into account. More details are provided in Table 1.

The evolution of CTC counts relative to baseline was analyzed in three reports. Specifically, the conversion from <5 CTCs to ≤5 CTCs/7.5 mL blood (CellSearch^®^ assay, Menarini Silicon Biosciences, Bologna, Italy) is associated with significantly worse OS; similarly, conversion from ≥5 to <5 CTCs/7.5 mL blood is associated with better PFS. CTC negativation (from detectable to undetectable CTC counts) was linked with improved outcomes, and likewise a lack of CTC negativation was linked to shorter OS. Detailed results are also presented in Table 1 below.

CTC nuclear size was evaluated in a single study, an abstract by Gill et al. (2017) [70]: in American mCRPC patients treated with first-line ABI, a nuclear diameter over 23.8 μm was not associated with a statistically significant difference in outcomes (median PFS 5.8 versus 6.8 months, *p* = 0.3). The prevalence of the alteration was not reported. The paper does not follow the STROBE checklist.

ctDNA fraction was evaluated in 12 reports. Typically, each paper reported a different threshold for ctDNA positivity, ranging from 1% to 30%—in some cases, the investigators used median ctDNA fraction as a threshold. Significant correlations with worse outcomes (i.e., shorter time to progression, PFS, or OS) were identified in all included reports. More detailed information is provided in Table 2.

cfDNA concentration was investigated in three papers. All three studies reported a significantly lower OS in the cfDNA-high group; PSA PFS and clinical-radiographic PFS were reported in one study and were likewise worse in cfDNA-high patients (see Table 2).

Finally, tdEV concentration was analyzed in one Belgian study [30] of ABI/ENZ (prior treatment allowed), which reported worse PFS (HR 2.42, 95% CI 1.56–3.73) and OS (HR 4.52, 95% CI 2.45–8.34) in the tdEV-high group, a result that lost statistical significance when controlling for prior ChT, baseline PSA, and CTC counts. The prevalence of the alteration was not reported. The article followed the REMARK checklist.

### 3.3. AR-Based Markers

By far the best studied AR-related biomarkers are AR splice variant 7 (28 reports: [12,33,40,47,48,49,50,71,72,73,74,75,76,77,78,79,80,81,82,83,84,85,86,87,88,89,90,91]) and AR copy number gain or overexpression (26 reports: [35,41,47,50,54,56,59,60,63,64,67,74,75,80,82,83,87,92,93,94,95,96,97,98,99,100,101,102,103,104]); fewer studies have investigated AR point mutations/single-nucleotide variants (SNVs) (8 reports: [56,59,92,97,98,103,105,106]); other markers were analyzed in a few papers each.

AR-V7 was evaluated from various sources, primarily CTCs and RNA. It has demonstrated predictive power in treatment-naïve and pretreated patients and in patients treated with ABI, ENZ, or APA but not in a study of ABI/ENZ plus bipolar androgen therapy. The prevalence of AR-V7 positivity ranged from 8.7% to just over 50%. The studies are listed in Table 3.

Additionally, two articles evaluated the combination of detectable CTCs and AR-V7 to CTC-negative patients. Namely, a 2017 study by Antonarakis et al. indicates worse outcomes (PSA PFS HR 5.3, 95% CI 3.12–9.02; crPFS HR 5.32, 95% CI 3.03–9.35; OS HR 4.92, 95% HR 2.21–10.96; multivariable analysis) in American CTC+/AR-V7+ patients relative to CTC-negative patients (treatment with ABI/ENZ, prior ARSI or taxanes allowed); conversely, CTC+/AR-V7 negative patients had a significantly worse PFS but not a significantly different OS (PSA PFS HR 1.83, 95% HR 1.18–2.82; crPFS HR 2.14, 95% HR 1.33–3.44; OS HR 1.65, 95% HR 0.81–3.37; multivariable analysis). In total, 55.9% of participants were CTC+/AR-V7-, and 17.8% were CTC+/AR-V7+ [107]; the article adhered to the REMARK recommendations.

The same approach was followed by Schlack et al. in a 2020 study of ABI/ENZ-treated German patients [108]. CTC+/AR-V7+ patients had a shorter crPFS and OS (median PSA PFS 5 months, 95% CI 3.6–6.4 vs. 17 months, 95% CI 15.7–18.3; median crPFS 9 months, 95% CI 1.1–16.9 vs. not reached, median OS 15 months, 95% CI 7.9–22.1 vs. not reached) compared to CTC-negative patients. Similar but less striking results were reported for CTC+/AR-V7- patients: median PSA PFS 13 months (95% CI 6.8–19.2), median crPFS 10 months (95% CI 6.2–13.8), and median OS 28 months (95% CI 16.8–39.2); the study did not follow the REMARK checklist for biomarker studies.

Ample evidence is also available for AR copy number gain. AR amplification is associated with worse prognosis in both first-line and pretreated patients. In addition, AR overexpression was evaluated in four reports, three of which indicated that AR overexpression is significantly associated with shorter PFS. The results are presented in Table 4 below.

AR point mutations were investigated in eight studies, using both ABI and ENZ, and in a first or second-line setting. It should be noted that most identified reports in this category failed to achieve statistical significance and that the prevalence of these biomarkers is comparatively low. The results are presented in Supplementary Table S3.1.

Finally, 18 reports [44,46,52,59,83,89,98,106,109,110,111,112,113,114,115,116,117,118] investigated other AR-related biomarkers, ranging from AR-V7 on CTCs by immunohistochemistry (IHC) [112], nuclear-localized AR-V7 [89,113,114,119], to the number of detected AR splice variants [52], and others. Specifically, AR-V7 by IHC was associated with a numerically shorter (but not statistically significant) PSA PFS, nuclear-localized AR-V7 was linked with worse outcomes in three of four studies, and the detection of any AR variants, but not the number of detected variants, is associated with worse PFS and OS. All 18 reports are presented in more detail in Supplementary Table S3.2.

### 3.4. Epigenetic Markers

Epigenetic markers were investigated in a number of papers: microRNAs (miRNA) are the best studied, with five reports; long non-coding RNAs (lncRNA) (specifically, NAALADL2-AS2) were evaluated in two studies, histone modifications in two; while circular RNAs (circRNA) and DNA methylation were each studied in one paper. In all cases, non-coding RNAs were evaluated in total plasma RNA, while DNA methylation was studied in cfDNA and histone modifications in CTCs.

miR-375 was evaluated in four reports. High expression of miR-375 was shown to be a significant predictor of PFS (HR 5.26, 95% CI 2.13–12.9) by Benoist et al. (2020), who studied Dutch patients treated with ENZ who were allowed to have received DTX in the castration-sensitive stage; 36.1% of patients were considered miR-375-high [120]. The results retained significance in multivariable analysis. Two abstracts from the same team came to similar conclusions: specifically, Boerrigter et al. (2020) [121] in ABI-treated patients, who described a numerically shorter PFS in treatment-naïve patients (PFS 352 vs. 456 days, *p* = 0.076), as well as Boerrigter et al. (2021) [88] in patients who had received DTX (PFS HR 1.78, 95% CI 0.93–3.41) [88]. On the other hand, miR-375 was demonstrated by Zedan et al. (2020) to be significantly associated with a shorter time to radiographic progression (HR 2.17, 95% CI 1.14–4.19) but not overall survival (HR 1.403, 95% CI 0.74–2.71) in an ABI-treated Danish cohort [122]. Prior treatments, multivariable analyses, or biomarker prevalence were not reported. The three studies of Dutch patients followed the relevant checklists (REMARK for Benoist et al. (2020) [120], STROBE for Boerrigter et al. (2020) [121], and Boerrigter et al. (2021) [88]).

miR-141 was investigated in two reports: by Zedan et al. (2020) in Danish patients starting ABI [122] and by Sharova et al. (2021) in Italian patients initiating ABI/ENZ [123]. Both reports correlate miR-141 overexpression with a significantly shorter OS (HR 3.202, 95% CI 1.25–9.37 and HR 3.82, 95% CI 1.14–12.79, respectively), respectively, as well as a worse time to radiographic progression (HR 3.18, 95% CI 1.31–7.32) and clinical or radiographic PFS (HR 7.43, 95% CI 2.18–25.37), respectively. Prior treatments were not stated in Zedan et al., while Sharova et al. allowed prior DTX. Multivariable analyses were only performed by Sharova et al.—miR-141 was not a significant predictor when adjusting for clinical parameters and the expression of other miRNAs. The proportion of miR-141-high patients was not reported by either study. Sharova et al. (2020) [123] generally followed the REMARK checklist.

miR-3687 was evaluated in two Dutch studies of ABI: (Boerrigter et al. (2021) [88]) and, respectively, ENZ-treated patients (Benoist et al. (2020) [120]). Both papers indicate a shorter PFS in the miR-3687-high group, but only the latter achieved statistical significance (HR 2.41, 95% CI 0.73–7.99 in the ABI study [88] and 4.57. 95% CI 1.86–11.2 in the ENZ study [120]). Prior DTX was allowed in both studies; in the latter only in a castration-sensitive context. In total, 30.6% of patients were positive for miR-3687 in the latter study, and the results retained significance in multivariable analysis [120].

High expression of NAALADL2-AS2 was evaluated in the same two studies. NAALADL2-AS2 expression above the cutoff was a significant predictor of better prognosis (PFS HR 0.23, 95% CI 0.07–0.82) in the ENZ study of Benoist et al. [120] but not in the ABI study of Boerrigter et al. [88] (PFS HR 0.60, 95% CI 0.31–1.15). Nonetheless, the association lost statistical significance when adjusting for possible confounders. In total, 31.6% of patients were NAALADL2-AS2-high in Benoist et al. (2020) [120].

Finally, miR-21, miR-93, miR-103a, miR-125b-1-5p, miR-221-3p, and miR-223 were each evaluated in one report. miR-21 underexpression was correlated by Sharova et al. with a significantly worse prognosis in Italian patients treated with ABI/ENZ (crPFS HR 7.38, 95% CI 2.56–21.25 and OS HR 5.16, 95% CI 1.70–15.70); however, the association was not significant in multivariable analysis [123].

In the same study, a low expression of miR-223 was also associated with worse outcomes (crPFS HR 3.52, 95% CI 1.52–8.13, OS HR 3.82, 95% CI 1.27–11.52); once again, the association was not significant in multivariable analysis [123].

Detassis et al. (2024) investigated the correlation between the overexpression of miR-103a-3p and PFS in Italian patients receiving ABI (prior DTX, CBZ, or ENZ were permitted) [124]. The authors identified a significantly shorter PFS in miR-103a-high patients (median PFS approximately 115 vs. 265 days, log-rank *p* < 0.001). No multivariable analysis was reported nor the proportion of miR-103a-high patients [124]. The study did not adhere to the REMARK guidelines.

miR-93-5p and miR-125b-1-5p were not significantly associated by Zedan et al. (2020) with either time to radiographic progression or OS (miR-93-5p: TTRP HR 2.38, 95% CI 0.91–6.28 and OS HR 1.66, 95% CI 0.62–4.66; miR-125b-1-5p: TTRP HR 1.27, 95% CI 0.74–2.18 and OS HR 1.502, 95% CI 0.82–2.67) [122]. Finally, miR-221-3p overexpression was significantly correlated with a worse OS (HR 2.36, 95% CI 1.03–5.34) but not with time to progression (TTRP HR 1.33, 95% CI 0.69–2.53).

There was one study of circular RNA species, namely, Tao et al. (2023), who evaluated a cohort of Chinese patients treated with first-line ABI [125]. The underexpression of hsa_circ_0113177, hsa_circ_0127731, hsa_circ_0002048, hsa_circ_0097211, hsa_circ_0116020 and hsa_circ_0002910 was associated with shorter OS. Additionally, a 5-RNA model based on circCEP112 (hsa_circ_0002910), circFAM13A (hsa_circ_0070440), circBRWD1 (hsa_circ_0116020), circVPS13C (hsa_circ_0000607), and circMACROD2 (hsa_circ_0114706) was associated with significantly worse PFS and OS in one testing and two validation cohorts. The authors adhered to the REMARK checklist.

Peter et al. (2022) investigated the correlation between cfDNA methylation patterns and time to progression in patients treated with first-line ABI/ENZ [126]. None of the nine analyzed genomic regions showed significant correlations with PFS when evaluated at baseline. It should be noted that eight of the nine regions are significantly associated with outcomes when evaluated after the start of treatment (which was outside the scope of this review). The authors did not closely adhere to the REMARK checklist.

Regarding histone modifications, SIRT2 expression in CTCs, which is inversely correlated with H3K18 acetylation, was evaluated by Filon et al. (2023) in a cohort of variously pretreated (ARSI, taxanes, immunotherapy, sacituzumab govitecan, radium-223) American mCRPC patients initiating ABI/ENZ [127]. Patients with SIRT2 expression in the highest quartile had a numerically better PFS compared to patients with SIRT2 expression in the lower three quartiles, but the result was not statistically significant (HR 0.53, 95% CI 0.21–1.32). No multivariable analysis was reported. The authors generally did not follow the REMARK recommendations.

Alterations in KMT2C and KMT2D, two histone-modifying lysine methyltransferases, were investigated by Mizuno et al. (2021) in Japanese patients treated with ABI/ENZ (previous treatment with the other ARSI permitted) [111]. No significant associations were detected (PFS HR 1.51, 95% CI 0.64–3.56, OS HR 1.62, 95% CI 0.49–5.40).

### 3.5. DNA Damage Response and Homologous Recombination Response Pathways

Several markers referring to the DNA damage response (DDR) and homologous recombination response (HRR) pathways, namely, TP53 (DDR), BRCA2 (HRR), and BRCA1 and ATM (both pathways), were investigated in a number of studies, either individually or pooled with one another.

Specifically, TP53 alterations were found to be significantly associated with shorter PFS or time to progression in all but one of the seven [46,56,59,64,98,109,111] included reports; detailed results are presented in Supplementary Table S3.3.

ATM alterations were investigated in one 2021 study by Dong et al. [103] of Chinese patients who received ABI in a first-line or later-line context. The prevalence of ATM alterations detected in cfDNA was 7.5%, and the study failed to prove a significant association between the biomarker and outcomes (PFS HR 1.613, 95% CI 0.382–6.811); no multivariate analysis was reported.

BRCA2 alterations were evaluated in four reports: two studies of BRCA2 copy number gain and two for BRCA2 deleterious mutation or loss, with inconsistent results. Gupta et al., in a 2020 study of American patients treated with ABI/ENZ (pretreatment with the other ARSI was permitted), BRCA2 gain in CTCs (prevalence 25%) was associated with worse outcomes (rPFS HR 4.3, 95% CI 1.9–9.8, multivariate analysis not performed) [80], while Du et al. (2020), investigating American patients treated with first-line ABI, found a lower risk of death in BRCA2-amplified (evaluated in cfDNA, prevalence not reported) patients (OS HR 0.42, 95% CI 0.19–0.90), which lost significance in multivariate analysis [60].

BRCA2 deleterious mutations or copy number loss was evaluated in two cfDNA studies: Shevrin et al. (2021) reported a shorter median OS in American patients with BRCA2 alterations (prevalence not reported) treated with ABI/ENZ (23 vs. 38 months, *p* = 0.022, multivariate analysis not reported) [128], while Dong et al., in a cohort of ABI-treated Chinese patients (prior treatment allowed), did not demonstrate a significant correlation between BRCA2 alterations (prevalence 10%) and outcomes (PFS HR 0.719, 95% CI 0.22–2.346, multivariate analysis not reported) [103]. The abstract by Shevrin et al. did not closely follow the STROBE checklist for abstracts of observational studies.

Multiple DDR (other than TP53) or HRR markers were evaluated in six reports [56,59,64,109,111,129], in all cases from cfDNA, sometimes with conflicting results. Namely, BRCA2 or ATM alterations were found to be significantly associated with worse outcomes in two studies [56,109], whereas a third paper [59] indicated no significant association between outcomes and the presence of either BRCA1/BRCA2/ATM deleterious mutation or copy number loss, or BRCA1/BRCA2/ATM deleterious mutation alone. More detailed results are presented in Supplementary Table S3.4.

HRR pathway defects were associated with worse outcomes in two studies of ABI/ENZ: by Zhang et al. (2022) in American patients (PFS HR 2.42, 95% CI 1–5.9) [129], and by Mizuno et al. (2021) [111] in Japanese patients (PFS HR 2.83, 95% CI 1.40–5.73 and OS HR 1.86, 95% CI 0.76–4.59).

The DDR pathway as a whole was investigated by Jayaram et al. (2021) [64] in an international multicenter study: alterations in the DDR pathway are associated with worse radiographic PFS (HR 2.13, 95% CI 1.30–3.48, significant in multivariate analysis) but similar OS (HR 1.32, 95% CI 0.78–2.30); the prevalence of DDR alterations was 17.2%.

### 3.6. Cell Cycle Regulation-Related Markers

Of the genes involved in cell cycle regulation, most available evidence refers to RB1 alterations (eight reports [56,59,60,64,97,98,109,111], as well as one report of RB1 and TP53 [59], one of RB1 or TP53 [103], and one of RB1 underexpression and E2F overexpression [129]).

RB1 alterations (deleterious mutations or copy number loss) were linked to worse outcomes in four of the seven studies that evaluated PFS/time to progression and in four of the five studies evaluating OS, associations that generally lost their significance in multivariable analysis. Detailed results are presented in Supplementary Table S3.5.

The combination of TP53 and RB1 alterations in cfDNA was investigated by Torquato et al. (2019) [59] in American mCRPC patients: the authors reported a numerically worse (but not statistically significant) PFS and a statistically significant shorter OS (PFS HR 1.66, 95% CI 0.71–3.91, OS HR 4.5, 95% CI 1.79–11.28; OS was significant after controlling for ctDNA fraction). The patients received treatment with ABI or ENZ, and prior treatment was allowed. In total, 9.7% of the studied cohort presented both alterations.

Either TP53 or RB1 alteration in cfDNA was studied by Dong et al. (2021) [103] in ABI-treated Chinese patients: the authors identified a strikingly worse PFS in patients with either alteration (HR 7.401, 95% CI 2.935–18.665). Prior treatment was allowed, the prevalence of either alteration was 13.8%, and multivariable analyses were not performed.

The presence of RB1 underexpression and E2F overexpression by CTC RNA sequencing was reported in an abstract by Zhang et al. (2022) [129] to be associated with shorter PFS in American patients treated with ABI or ENZ (prior treatment with the other agent was permitted): HR 3.5, 95% CI 1.5–8.2. The prevalence of the alteration was not reported.

### 3.7. PI3K Pathway

The PI3K pathway as a whole was investigated in three reports [56,59,111], PTEN alone in six studies [33,60,64,80,98,100], and PIK3CA in three reports [60,64,100].

The presence of any PI3K pathway alterations was linked with worse PFS/time to progression in two of the three studies. On the other hand, PTEN alteration or loss was significantly associated with shorter PFS or OS in a majority of identified papers, while PIK3CA gain in cfDNA was significantly associated with worse OS in all three studies and with shorter PFS in one of two studies reporting PFS. More detailed results, including the proportion of patients with the respective alterations and multivariable analyses, are reported in Supplementary Table S3.6.

### 3.8. WNT Pathway

Of the genes relevant to the WNT pathway, APC was evaluated in one study [66], WNT5B in two [82,87], and the WNT pathway as a whole in three studies [56,59,111]. Key findings are presented in this section, and detailed results are presented in Supplementary Table S3.7.

The presence of any WNT pathway alterations was not significantly associated with PFS/time to progression in any of the three studies but was correlated with worse OS in one of the two studies reporting this outcome. An APC copy number above the median (in cfDNA) was associated with shorter OS, while WNT5B overexpression in CTCs was linked to shorter crPFS and PSA PFS.

### 3.9. PSA (KLK3), PSMA, and PSCA

The prostate cancer biomarkers PSA (also known as KLK3), PSMA, and PSCA were evaluated in six [50,78,79,82,87,88], four [50,130,131,132], and two [82,87] papers, respectively. KLK3 was evaluated in circulating RNA in three reports and CTCs in the other three. PSMA and PSCA were only evaluated in CTCs.

Regarding KLK3, only three of the seven cohorts (from six reports) that evaluated PFS/time to treatment failure reported a significant association of KLK3 overexpression with outcomes. PSMA was investigated in four reports, of which three show a significantly worse PFS in patients with PSMA-positive CTCs. PSCA was evaluated in two reports; both papers suggest a significant association between PSCA expression and worse biochemical, clinical, and radiographic progression. More detailed results are presented in Supplementary Table S3.8.

### 3.10. Other Individual Biomarkers

Finally, 34 more biomarkers were identified, which were investigated in one or at most two papers. In alphabetical order, these are AKR1C3, ANXA2, BMP7, BRAF, CDK12 (two studies), CYP17A1, FOXA1, GAS6, GR, GRHL2, GSTP1, HER2, HOXB13 (two studies), KLK2, MET, MYC, MYCN, NBN, NCOA2, NCOR1, NF1, NKX3–1 (two studies), OCT4, OPHN1, PD-L1, RUNX2, SOX2, SPINK1, SPOP, SYP, TMPRSS2-ERG fusion (two studies), TSPAN8 (two studies), TUBB3, and ZHFX3 (two studies). The main results of these studies are presented below.

High exosomal expression of AKR1C3, which codes for a key steroidogenic enzyme, was found to be significantly associated with worse outcomes in Chinese patients treated with ABI (PFS HR 3.81, 95% CI 1.69–8.58; OS HR 5.41, 95% CI 2.44–12.01), a result that retained significance in multivariate analysis; additionally, the prevalence of high exosomal AKR1C3 is relatively high at 24.2% [133]. The study did not closely adhere to the REMARK guidelines.

ANXA2 (annexin A2) expression in CTCs was associated by Morgan et al. (2017) with shorter PFS in American patients treated with ABI/ENZ (PSA PFS HR 3.53, 95% CI 1.06–11.70 and crPFS HR 7.9, 95% CI 1.83–34.05) [87].

BMP7 expression in CTCs was correlated by Chung et al. (2017) with shorter PSA PFS in American ABI/ENZ-treated patients (HR 1.46, 95% CI 1.09–1.95) [131]. The authors adhered to the STROBE checklist.

The detection of BRAF alterations in cfDNA was associated by Shevrin et al. (2021) with a shorter OS (*p* = 0.008) in American mCRPC patients treated with ABI/ENZ; median OS was 24 months in participants with BRAF alteration, compared to 58 months in all patients [128].

The detection of CDK12 amplification in cfDNA was linked by Du et al. (2020) to improved OS in American patients treated with first-line ABI (HR 0.37, 95% CI 0.15–0.96), but the result did not retain statistical significance in multivariable analysis [60]. Comparable results were obtained for CDK12 loss, which was associated with worse PFS in pretreated and treatment-naïve Chinese patients who received ABI (HR 3.877, 95% CI 1.57–9.59); 15.4% of the study participants presented this alteration [103].

CYP17A1 gain in cfDNA was correlated with shorter survival in an Italian study of ABI in a post-DTX context (PFS HR 2.79, 95% CI 1.45–5.35 and OS HR 2.61, 95% CI 1.24–5.48) [96]. The prevalence of CYP17A1 gain was 28.3%, and the results were significant after controlling for potential confounders.

FOXA1 detection in whole-blood RNA was linked by Todenhöfer et al. (2017) to marginally worse outcomes (PSA PFS HR 2.0, 95% CI 1.0–4.2 and OS HR 2.0, 95% CI 0.9–4.8) in Canadian patients treated with ABI (prior taxanes were allowed) [78]. One-third of participants were FOXA1-positive.

GAS6 positivity in CTCs was reported in one study to be associated with shorter PSA PFS (HR 2.29, 95% CI 1.16–4.51) in American patients treated with ABI or ENZ [131].

Glucocorticoid receptor (GR) protein expression was correlated with worse survival in American patients treated with ABI/ENZ (OS HR 4.16, 95% HR 1.23–14.0 in a multivariable analysis); additionally, the prevalence of GR positivity was relatively high, at 51.9% [29].

According to Todenhöfer et al. (2017), GRHL2 expression in whole-blood mRNA failed to demonstrate a significant association with outcomes (PSA PFS HR 1.5, 95% CI 0.7–3.1, OS HR 2.1, 95% CI 0.9–4.7) in Canadian patients treated with ABI [78].

Above-median GSTP1 expression in cfDNA was associated with shorter OS in treatment-naïve Dutch patients who received ABI or ENZ (median OS ~19 months vs. not reached, *p* = 0.02) [66].

The detection of HER2-positive CTCs was identified in a large proportion (63%) of pretreated French patients and was significantly correlated with shorter PSA PFS and rPFS (PSA PFS HR 3.35, 95% CI 1.3–8.7, cPFS HR 3.59, 95% CI 0.8–16.4, rPFS HR 4.26, 95% CI 1.4–12.9), a result that retained significance in multivariable analysis [134]. The article does not follow the REMARK guidelines.

HOXB13 positivity was evaluated in two papers: in whole-blood mRNA by Todenhöfer et al. (2017) [78] and in CTCs by Halabi et al. (2024) [135]. The former study investigated Canadian patients managed with ABI and allowed prior treatment with taxanes, while the latter evaluated American patients who received ABI or ENZ and allowed prior treatment with the other ARSI. The prevalence of HOXB13 positivity was slightly higher in the former study (51.9% vs. 44%). Both studies reported significantly worse outcomes in the HOXB13 positive group (Todenhöfer et al.: PSA PFS HR 2.8, 95% CI 1.3–6.8 and OS HR 3.0, 95% CI 1.2–9.3 vs. Halabi et al.: rPFS HR 3.17, 95% CI 1.86–5.40 and OS HR 2.76, 95% CI 1.54–4.93—comparing HOXB13+/CTC+ patients to CTC-negative patients). The CTC study also demonstrated that HOXB13 is a significant biomarker after controlling for confounding variables, whereas no multivariable analysis was reported in the mRNA study. Halabi et al. (2024) [135] adhered to the REMARK checklist.

Todenhöfer et al. (2017) also investigated KLK2 in whole-blood mRNA in ABI-treated (prior taxanes allowed) patients: KLK2 positivity was identified in two thirds of participants and was significantly associated with shorter survival: PSA PFS HR 2.2 (95% CI 1.1–4.6), OS HR 2.4 (95% CI 1.1–6.1) [78].

cfDNA MET amplification was reported in one abstract of a study of Canadian mCRPC patients treated with ENZ (prior ABI and DTX were permitted): MET gain was reported in 13% of participants and was associated with significantly worse clinical and radiographic PFS (crPFS HR 4.5, *p* < 0.001) [106].

MYC amplification was investigated in Japanese patients by Mizuno et al. (2021): participants received either ABI or ENZ, and prior treatment with the other ARSI was permitted [111]. Significantly worse outcomes were reported in patients with MYC gain: PFS HR 4.63 (95% CI 2.23–9.64) and OS HR 6.53 (95% CI 2.78–15.4).

MYCN amplification in CTCs was described by Gupta et al. (2020) in 30% of American patients receiving ABI/ENZ either in a first-line context or after progression on the other ARSI [80]. The presence of this alteration is numerically correlated with shorter PFS but fails to reach statistical significance (rPFS HR 1.9, 95% CI 0.9–3.9).

Mizuno et al. also determined that NBN amplification is associated with shorter survival during ABI/ENZ therapy (PFS HR 3.63, 95% CI 1.72–7.65 and OS HR 6.19, 95% CI 2.48–15.5) [111].

NCOA2 alterations in cfDNA were studied by both Du et al. (2020) in American patients receiving first-line ABI [60] and by Fettke et al. (2021) in Australian patients receiving ABI/EBZ (first-line, after ChT or after the other ARSI) [98]. The former study reports a shorter overall survival in ABI-treated participants with NCOA2 gain (OS HR 1.59, 95% CI 1.01–2.52); however, this association loses significance in multivariable analysis [60]. On the other hand, NCOA2 gain is associated with much shorter OS and PFS in the latter study (PFS HR 21, 95% CI 5.8–77 and OS HR 9.6, 95% CI 3.1–30), both of which retain significance in multivariable analysis. Single-nucleotide variants in NCOA2 are reported by Fettke et al. to be correlated with worse OS but not PFS (PFS HR 0.75, 95% CI 0.18–3.1 and OS HR 3.1, 95% CI 1.1–8.8). The presence of any NCOA2 alteration is associated with worse OS (HR 5.3, 95% CI 2.3–12, also significant in multivariable analysis) but not PFS (HR 2.2, 95% CI 0.92–5.1). However, the prevalence of NCOA2 alterations as reported by Fettke et al. is rather low: 7.7% for NCOA2 copy number gain, 5.5% for NCOA2 single-nucleotide variants, and 13.2% for any NCOA2 alteration [98].

NCOR1 copy number gain in cfDNA is reported by Du et al. (2020) to be associated with improved prognosis (OS HR 1.59, 95% CI 1.01–2.52) in ABI-treated patients; however, this association loses significance after adjusting for confounding factors [60].

The detection of NF1 alterations in cfDNA is linked by Shevrin et al. (2021) to a statistically significant (*p* = 0.036) decrease in median survival (median OS 20 months in NF1-altered patients, compared with 58 months in NF1-altered and wild-type patients) [128].

NKX3-1 copy number gain was evaluated in two studies: by Chung et al. (2019) [82] in CTCs extracted from American patients starting treatment with ABI/ENZ (prior therapies were not reported) and by Du et al. (2020) [60] in cfDNA samples of American patients receiving first-line ABI. While Du et al. suggest that NKX3-1 amplification is associated with significantly better survival (OS HR 0.42, 95% CI 0.24–0.73), Chung et al. identified a shorter PFS in NKX3-1-amplified patients (median PSA PFS ~70 vs. ~400 days, *p* < 0.001, and median crPFS ~100 vs. ~650 days, *p* = 0.025).

According to Ma (2023), stem cell marker OCT4 positivity in CTCs was a relatively frequent biomarker (48.6%) and was linked to shorter PFS in Chinese patients receiving ABI as an unspecified line of treatment: rPFS HR 3.58 (1.78–7.20), OS HR 3.96 (1.43–10.91), comparing patients with detectable CTCs and OCT4 expression to patients with detectable CTCs but OCT4-negative and CTC-negative patients [136]. The result retained significance in multivariable analysis. The author generally followed the REMARK recommendations.

OPHN1 copy number gain was evaluated in cfDNA by Du et al. (2020) in American patients receiving first-line ABI: a significant association with shorter OS was identified (HR 1.94, 95% CI 1.22–3.09), which retained significance in multivariable analysis [60].

The expression of immune checkpoint PD-L1 mRNA in plasma exosomes was studied by Conteduca et al. (2022) in treatment-naïve Italian patients receiving ABI/ENZ; above-median PD-L1 expression was associated with a significantly shorter rPFS (HR 3.1, 95% CI 1.1–8.5), a result that retained significance after adjusting for potential confounding variables [137]. The abstract followed the STROBE checklist for abstracts.

Gupta et al. (2020) also identified RUNX2 loss in CTC-derived DNA in 30% of patients initiating ABI/ENZ (prior treatment with the other agent was permitted) in a United States study. The alteration had a significant impact on PFS (rPFS HR 1.25, 95% CI 0.625–2.5) in univariate analysis [80].

In a 2017 abstract by Morgan et al., SOX2 expression in CTCs was associated with worse outcomes in American patients starting ABI/ENZ (PSA PFS HR 3.7, 95% CI 1.11–12.25 and crPFS HR 4.81, 95% CI 1.35–17.05) [87].

SPINK1 loss in CTCs was linked by Chung et al. (2019) with shorter median PFS in American patients receiving ABI/ENZ (median PSA PFS ~45 vs. ~140 days and median crPFS ~35 vs. ~200 days, both *p* < 0.001) [82].

The presence of SPOP mutation was investigated in Canadian patients starting ABI/ENZ in a first-line context: no significant association was identified (time to progression HR 1.00, 95% CI 0.51–1.97) [56].

Synaptophysin (SYP) expression in CTCs was linked to a shorter time to progression (*p* = 0.024, no HR or median time reported) in American patients receiving ABI/ENZ [43].

TMPRSS2-ERG fusion was investigated by Todenhöfer et al. (2017) in whole-blood mRNA from Canadian patients starting ABI (prior taxanes allowed) [78] and by Danila et al. (2011) in CTCs in American patients starting ABI (prior ARSI or ChT allowed) [39]. Neither study managed to demonstrate a significant association between TMPRSS2-ERG fusion and outcomes (Todenhöfer et al.: PSA PFS HR 2.0, 95% CI 0.7–4.6 and OS HR 1.8, 95% CI 0.5–4.8; Danila et al.: median OS 64 vs. 68 weeks, *p* = 0.782). The prevalence of TMPRSS2-ERG fusion was lower in Todenhöfer et al.’s mRNA assay (14.8%) compared to Danila et al. (37%).

TSPAN8 expression in CTCs was investigated by the same team in American patients receiving ABI/ENZ in two different studies: the abstract of Chung et al. (2017) reported worse biochemical survival in biomarker-positive patients (PSA PFS HR 2.04, 95% CI 1.21–3.46) [131], which was confirmed in the full text by Chung et al. (2019) (median PSA PFS ~70 vs. ~230 days, *p* = 0.007 and median crPFS ~80 vs. ~285 days, *p* = 0.028) [82].

TUBB3 expression was evaluated by Zhu et al. (2021) [138] in exosomes extracted from Chinese patients initiating first-line ABI: TUBB3-high patients had significantly shorter biochemical PFS, as well as a shorter OS—the latter narrowly missing the significance threshold (PSA PFS HR 2.271, 95% CI 1.152–4.475 and OS HR 2.137, 95% CI 0.915–4.989). The association with PSA PFS retained significance in multivariable analysis; additionally, the proportion of TUBB3-positive patients was rather high, at 42.3% [138]. The authors generally adhered to the REMARK checklist.

ZFHX3 was investigated in two cfDNA studies: ZFHX3 alterations (specifically, point mutations and two-base microindels) by Mizuno et al. (2021) [111] in Japanese patients receiving ABI/ENZ (prior ARSI permitted) and ZFHX3 copy number gain by Du et al. (2020) [60] in American patients starting first-line ABI. The former study identified a significant negative impact of ZFHX3 alterations on outcomes (PFS HR 3.76, 95% CI 1.88–7.55 and OS HR 11.9, 95% CI 4.99–28.6); likewise, ZFHX3 amplifications were linked to improved survival (OS HR 0.43, 95% CI 0.24–0.75) and retained significance in multivariable analysis.

### 3.11. Multiple Biomarkers

A number of studies investigated the impact of multiple molecular markers simultaneously. We also include in this section large-scale alterations such as large (chromosomal arm) indels, alterations in multiple genes relevant to a certain pathway or other gene panels, and genome-wide alterations (phenotypic diversity, chromosomal instability).

Chromosomal instability was investigated in CTCs by Brown et al. (2021) in American patients starting ABI/ENZ [139] and by de Bono et al. (2021) in an international multicenter ABI/ENZ trial [140]. Only the former trial proved a significant association between a chromosomal instability phenotype and worse outcomes (Brown et al.: crPFS HR 2.3, 95% CI 1.5–3.6 and OS HR 3.2, 95% CI 2.0–5.1, also significant in multivariable analysis; de Bono et al.: rPFS HR 0.84, 95% CI 0.52–1.38 and OS HR 1.57, 95% CI 0.92–2.67 in a multivariable analysis). Prior treatment with the other ARSI was allowed in the former trial, and prior treatment with taxanes as well as the other ARSI was allowed in the latter. The proportion of biomarker-positive patients in Brown et al. (2021) [139] was 36.4% and 49.3% in de Bono et al. (2021) [140], respectively.

Phenotypic heterogeneity in fluorescence microscopy slides of CTCs was evaluated independently in two studies. Oeyen et al. (2021) obtained significant results in Belgian patients receiving ABI/ENZ (PFS HR 1.79, 95% CI 1.07–2.98 and OS HR 2.32, 95% CI 1.09–4.96); however, the results lost significance when controlling for prior treatment, baseline PSA, and CTC counts [30]. In the second study, Scher et al. (2017) investigated American patients commencing ABI/ENZ/APA (prior ARSI or ChT were permitted) and identified significantly worse OS in patients with highly heterogeneous CTCs (OS HR 3.84, 95% CI 2.11–7), a result that retained significance in multivariable analysis [141]. Both studies adhered to the REMARK checklist.

Two reports of the same multicenter international trial of ABI/ENZ in treatment-naïve and treatment-experienced patients, namely, De Laere et al. (2019) [142] and De Laere et al. (2021) [143] investigated whether a high number (at least four) of driver gene mutations in cfDNA and, respectively, a large pathway complexity (mutations in at least three oncogenic pathways in cfDNA) are associated with survival. Both the former (PFS HR 1.85, 95% CI 1.06–3.23) and latter (PFS HR 1.7, 95% CI 1.02–2.84 and OS HR 2.5, 95% CI 1.06–5.71) were associated with worse outcomes in multivariate analysis. The proportion of patients with at least four driver mutations was 20.8%, very similar to that of patients with at least three affected pathways (20.2%). Both studies adhered to their respective checklists (STROBE for abstracts of observational studies for De Laere et al. (2019) [142] and REMARK for De Laere et al. (2021) [143]).

Genome-wide aneuploidy in cfDNA was evaluated independently in three studies. Kohli et al. (2018), investigating American ChT-naïve patients receiving ABI, obtained significantly worse OS (HR 1.63, 95% CI 1.14–2.31) in patients with a high “Plasma Genome Abnormality” (a CNV-based score) [38]. Isebia et al. (2023), in Dutch patients receiving ABI or ENZ, associated a high genome-wide aneuploidy score with shorter time under treatment (82 days vs. 221 days, *p* < 0.001) [144]. The authors adhered to the STROBE guidelines for abstracts. Finally, the impact of a high CNV burden was evaluated by Nørgaard et al. (2023) in Danish patients starting ABI/ENZ in a first-line setting (prior DTX in the castration-sensitive stage was permitted) [53]. Specifically, patients with a high CNV burden had worse PSA PFS and OS (PSA PFS HR 2.38, 95% CI 1.60–3.54 and OS HR 13.01, 95% CI 2.93–57.85); however, the results narrowly failed to achieve statistical significance when controlling for ctDNA fraction.

Additionally, Nørgaard et al. (2023) also reported four large-scale indels that were linked to worse outcomes: chromosome 13q deletion (PSA PFS HR 2.86, 95% HR 1.52–5.36; prevalence 38.9%), chromosome 7 amplification (OS HR 4.53, 95% CI 1.68–12.2; prevalence 35.7%), chromosome 8p deletion (OS HR 3.86, 95% CI 1.39–10.7; prevalence 44.8%), and chromosome 9q amplification (OS HR 2.64, 95% CI 1.02–6.81; prevalence 29.4%), results that retained significance after controlling for ctDNA fraction [53].

Six studies evaluated panels of multiple genes. Chung et al. (2019) investigated the prognostic potential of a score based on the high expression of eight genes/variants in CTCs, namely, AR, AR-V7, NKX3.1, PSA, PSCA, TSPAN8, WNT5B, and SPINK1, in American patients starting ABI or ENZ [82]. Patients with a high score tended to have shorter PFS (median PSA PFS ~60 vs. ~145 days, *p* = 0.026, and median crPFS ~80 vs. ~300 days, *p* = 0.011).

Haas et al. (2021) reported that another CTC-based, eight-gene model (MSLN, ARG2, FGF8, KLK3, ESRP2, NPR3, CCND1, and WNT5A) was associated with significantly worse outcomes (PFS HR 2.95, 95% CI 1.46–5.98 and OS HR 5.32, 95% CI 1.91–14.8) in American patients starting ABI/ENZ [145]. The article generally adhered to the REMARK recommendations.

Groen et al. identified a CTC-based gene signature (referred to as “ARSI 1”) that was strongly predictive of PFS and OS (PFS HR 13.05, 95% CI 2.47–68.92 and OS HR 10.78, 95% CI 2.35–39.42) in Dutch patients initiating ABI or ENZ. Moreover, the results retained statistical significance when adjusting for age, baseline PSA, and AR-V7 expression [146]. The article was not structured according to the REMARK recommendations.

An abstract by Halabi et al. (2024) evaluated a cfDNA model based on the presence of copy number gain in AR, AR enhancer, MYC, and RSPO2, and copy number loss or pathogenic mutations in APC, BRCA2, CHD1, FANCA, MSH6, NKX3-1, PPP2R2A, PTEN, RB1, TP53, and ZBTB16, as well as ctDNA fraction, in American patients starting ENZ with or without ABI [147]. Patients defined as low risk based on this score had an almost five times lower risk of death (OS HR 0.22, 95% CI 0.17–0.27) compared to high-risk patients; intermediate-risk patients likewise had a better prognosis (OS HR 0.42, 95% CI 0.35–0.51). The article adhered to the STROBE checklist.

McKay et al. (2021), evaluating American patients starting ENZ (previous ABI, ketoconazole or ChT allowed), linked the presence of either AR variants or synaptophysin or at least two of KLK2, LKL3, TMPRSS2, FOLH1, or NKX3-1 in CTCs with a shorter time to radiographic (HR 2.57, 95% CI 0.56–11.88) and PSA progression (HR 1.18, 95% CI 0.3–4.57), as well as OS (HR 6.29, 95% CI 1.22–32.5) [148]. The paper adhered to the REMARK guidelines.

Kwan et al., investigating Australian patients initiating ABI or ENZ (previous treatment with the other agent or ChT permitted), connected the presence of aberrations in either the AR or the PTEN/PI3K/AKT pathways with worse outcomes: cPFS HR 2.5 (95% CI 1.1–6.1) and OS HR 3.1 (95% CI 1.1–8.8) for one alteration, cPFS HR 4.6 (2.0–11), OS HR 5.6 (2.2–14) for two or more alterations [100]. In total, 28.2% of patients presented one aberration, and 44.9% presented two or more. In multivariable analysis, the presence of one aberration was only significant for OS, while two or more aberrations were significant for both cPFS and OS.

Six studies evaluated pairs of biomarkers. Specifically, Miyamoto et al. (2012) correlated the presence of >10% CTCs positive for both PSA and PSMA (by immunofluorescence) with shorter median survival (median OS ~180 days vs. not reached, *p* = 0.048) in American patients beginning ABI who had not received ARSIs in previous lines of treatment [149]. The study did not follow the REMARK checklist for biomarker studies.

The combination of CTC clusters and AR-V7 was evaluated by Okegawa et al. (2018) in Japanese patients starting ABI or ENZ [150]. The presence of CTC clusters and no AR-V7 expression was linked to worse outcomes compared to CTC cluster-negative patients (PSA PFS HR 3.9, 95% CI 1.8–5.1; rPFS HR 3.3, 95% CI 2.1–6.2; OS HR 3.7, 95% CI 1.4–6.1). The risk of progression or death was even higher in participants positive for both CTC clusters and AR-V7 (PSA PFS HR 7.1, 95% CI 2.5–11.3; rPFS HR 5.6, 95% CI 2.4–7.4; OS HR 4.8, 95% CI 2.1–5.6). In total, 23.5% of patients were positive for CTC clusters but not AR-V7, and 26.5% were positive for both. The results remain significant in multivariable analysis. The authors followed the REMARK guidelines.

Qu et al. (2017) evaluated the combination of PSA (KLK3) positivity and high AR-V7 expression in whole-blood RNA extracted from American patients commencing ABI or ENZ (prior taxanes were permitted, as well as treatment with the other ARSI) [79]. The above-mentioned marker combination was associated with worse outcomes in both the ABI and ENZ cohorts (time to treatment failure HR 2.82, 95% CI 1.11–7.18 in ABI-treated patients and 3.08, 95% CI 1.02–9.30 in ENZ-treated patients; both results by multivariable analysis). 25.9% in the ABI cohort and 25.5% in the ENZ cohort were PSA-positive and had a high AR-V7 expression.

The combination of BRAF and AR alterations in cfDNA was investigated by Shevrin et al. (2021) in American patients starting ABI or ENZ, who identified a shorter median overall survival in patients positive for both alterations (median OS 18.7 months, compared to 58 months in the overall sample, *p* = 0.031) [128].

AR and NCOA2 alterations in cfDNA were studied by Fettke et al. (2021) in Australian patients starting ABI/ENZ [98]. Patients with both AR and NCOA2 alterations (7.7% of patients) had the highest risk for progression or death (PFS HR 7.4, 95% CI 2.5–22 and OS HR 5.3, 95% CI 1.8–16); the predictor retained significance for both PFS and OS after adjusting for clinical and molecular covariates. The presence of aberrations in either AR or NCOA2 (40.7% of patients) was also associated with worse outcomes (PFS HR 1.8, 95% CI 1.0–3.1 and OS HR 3.1, 95% CI 1.7–5.8); in multivariate analysis, this was only significant in the case of PFS. The presence of AR or NCOA2 copy number gain (25.3% of patients) had a similar prognostic significance (PFS HR 2.5, 95% CI 1.3–4.7 and OS HR 3.2, 95% CI 1.7–6.2); this predictor was significant in multivariable analyses for both PFS and OS.

Finally, the identification of either SPOP or CHD1 alterations in cfDNA was linked to shorter OS, but not PFS, in Japanese patients receiving ABI/ENZ (prior treatment with the other ARSI was permitted): PFS HR 2.14 (95% CI 0.85–5.42) and OS HR 8.72 (95% CI 3.37–22.6) [111]. 

## 4. Discussion

The utility of liquid biopsy in predicting response to ARSIs cannot be overstated: it allows patients to avoid the invasiveness of a second biopsy and the futility of a potentially inefficient treatment. However, despite the more than 10-year history of the field (see Antonarakis et al. (2014) [12]) and its rapid development, the adoption of liquid biopsy in clinical practice is slow.

As stated before, the landscape of liquid biopsy research in mCRPC patients starting ARSI treatment is remarkably complex, encompassing both specific molecular markers (i.e., single gene products) and nonspecific markers that primarily reflect disease burden (such as CTC count, cfDNA concentration, ctDNA fraction), markers pertaining to androgen receptor signaling or to other pathways, and both well-studied (CTCs, AR-V7, AR copy number gain) and emerging biomarkers.

The main characteristics that we took into account to evaluate the predictive potential of a biomarker were the type of assay or biomaterial, prior treatment, the country/population in which the study was performed, and whether the results retained significance when adjusting for confounding variables. The latter is itself difficult to generalize, as the variables included in multivariable analyses (typically, those parameters that were found significant in univariate analysis) differed significantly from one study to another. The country is an imperfect predictor for shared genetic background, but previous experience (such as in non-small cell lung carcinoma, with a high prevalence of EGFR mutations in East Asian patients compared to Western or South Asian patients [151]) indicates that it is a potential confounder that needs to be addressed. Finally, the choice of ARSI was evaluated; however, a significant proportion of studies pooled ABI- and ENZ-receiving patients, despite their different mechanisms of action. Adherence to REMARK or other comparable guidelines was investigated; nonetheless, such checklists were designed to standardize reporting, rather than as quality assessment tools—for this reason we did not exclude from the analysis studies that did not comply with the checklists.

A simple classification based on the number of studies with positive (i.e., significant) results can highlight those biomarkers in the most advanced stage of research. Nonetheless, it must be said that the number of reports for each biomarker cannot compensate for shared limitations (i.e., if all studies were performed in patients with similar previous therapies, in patients with similar genetic ancestry, etc., or if the biomarker is too rare to be of practical use). Nonetheless, we can group the markers into four categories: best-studied biomarkers, with at least 10 reports, of which a majority obtained significant results; moderately well studied biomarkers, with three or more studies, of which a majority significant; potential biomarkers, with one or two studies, at least one significant; and finally the least promising biomarkers, which were not significantly associated with outcomes in any study. The classification of all identified biomarkers is presented in Table 5.

There are four biomarkers supported by the largest body of evidence: two disease burden biomarkers (CTC counts and ctDNA fraction) and two androgen signaling markers (AR-V7 and AR copy number gain or overexpression).

CTC counts were evaluated in 24 reports and in various circumstances: various assays or thresholds, different prior treatments, and in different ethnic groups.

ctDNA fraction was likewise studied in a large number of papers and in diverse therapeutic contexts; nonetheless, it shows a potential barrier to its wider applicability compared to CTC counts: the lack of studies in populations of non-European ancestry.

AR biomarkers, such as splice variant 7 and AR copy number gain or overexpression, are also well-investigated; however, only AR-V7 and to a limited extent (one study [103]) AR copy number gain were studied in non-European populations.

The second category includes markers evaluated in at least three studies. Generally, biomarkers in this group tended to also have more inconsistent results and more significant limitations than those in the first category. We include here the less-studied AR biomarkers (AR point mutations, nuclear localized AR-V7, various unspecified AR alterations, and certain splice variants other than AR-V7), one disease burden biomarker (cfDNA concentration), one better-studied epigenetic biomarker (miR-375), as well as markers that describe other processes or signaling pathways: TP53, BRCA2, RB1, PTEN, PIK3CA, the PI3K and WNT pathways, KLK3 (PSA), PSMA, and genome-wide aneuploidy. Three markers in this group, all well-known tumor suppressor genes, are noteworthy for having more consistent results: TP53, RB1, and PTEN. We will focus our discussion on the latter three and AR-related biomarkers. Additionally, we briefly mention miR-375, the only epigenetic biomarker supported by a larger body of evidence, as well as BRCA2.

AR point mutations were investigated in eight studies. Despite an apparently high number of reports, it shows some important limitations: of the eight identified studies, only one [92] specifically states the investigated SNVs, and only three studies report significantly worse outcomes; additionally, AR point mutations have a relatively low prevalence compared to other markers, which might limit their applicability.

Intuitively, the nuclear localization of AR-V7 should also influence prognosis, to an even greater extent than AR-V7 expression in general, since only nuclear-localized AR can influence transcription [152]. However, it was only evaluated (using immunofluorescence in CTCs) in four reports from the United States [89,113,114], three of which reported significantly worse outcomes. Interestingly, a fifth study, which evaluated AR-V7 by immunohistochemistry in CTCs, failed to prove a significant association [112].

Finally, unspecified AR alterations were evaluated in six reports: all studies reported significantly shorter PFS/TTP or OS in patients with AR alterations. Three studies evaluated unspecified AR splice variants in CTCs, all with significant results.

The well-known tumor suppressor TP53 is relatively well studied, with seven reports, of which six were statistically significant. The association between TP53 and worse outcomes was reported in both treatment-experienced and treatment-naïve patients, in both European or North American, as well as in Asian (specifically, Japanese [111]) populations; additionally, the prevalence of TP53 alterations was relatively high. Moreover, it has been documented in cfDNA in various solid tumors; interestingly, it was often detected only in liquid biopsy but not in tissue-based analysis [153]. TP53 mutations are common throughout various cancer types. According to the (tissue-based) Cancer Genome Atlas (TCGA) project, TP53 heterozygous deletions are moderately common in PCa, occurring in approximately one third of cases, but significantly less frequently than in other common solid tumors such as breast adenocarcinoma (51%) or rectal adenocarcinoma (64%) [154].

RB1, another tumor suppressor primarily involved in cell cycle regulation, is likewise relatively well investigated, with eight reports and generally significant results. All studies used cfDNA; three were performed in a first-line context, while the rest allowed prior treatment; one trial was performed in Japanese patients; and one was an international multicenter trial. However, the marker retained statistical significance in only one of the six studies that performed multivariable analysis. In addition to its well-described role in rare cancers such as retinoblastoma and osteosarcoma [155], RB1 alterations are encountered in a wide variety of cancers: TCGA indicates a high prevalence of RB1 heterozygous deletions in prostate adenocarcinoma (43%), as well as in common (lung squamous carcinoma—49%, breast—37%) and rare cancers (55–56% in kidney chromophobe carcinoma and pediatric osteosarcoma) [154]. Some evidence for liquid biopsy identification of RB1 in other cancers exists, primarily focusing on retinoblastoma (aqueous humor cfDNA [156]), but emerging evidence also exists for bladder urothelial carcinoma (urinary cfDNA and exosomes) [157].

PTEN loss was the best studied PI3K pathway biomarker, with six studies: two for PTEN negativity in CTCs, and the rest for cfDNA: PTEN alteration, copy number loss, or (in one study [60]) PTEN non-amplification vs. amplification. cfDNA PTEN loss (or non-amplification) was linked to worse OS in all four studies, and with worse PFS in one of the three studies that reported PFS. Nonetheless, multivariable analysis was only performed in one of the two CTC-based studies, which did not confirm PTEN as an independent predictor; additionally, all studies of PTEN were performed primarily in populations of European ancestry. A growing body of evidence supports the utility of cfDNA evaluation of PTEN in diverse solid tumors [158]. According to TCGA, PTEN heterozygous loss is moderately common in PCa (20%), similar to other common cancers such as breast cancer or lung adenocarcinoma.

A noteworthy case is that of BRCA2, which is associated with inconsistent, even contradictory results: in univariate models, BRCA2 gain in CTCs was associated with worse rPFS by Gupta et al. [80], while BRCA2 gain in cfDNA was linked with longer OS by Du et al. [60]; BRCA2 deleterious mutations or copy number loss were linked with worse OS in American patients [128] but not in a Chinese study [103].

miR-375 is the best studied epigenetic marker; nonetheless, three of the four studies were performed in the same clinic by the same team and presumably in similar populations [88,120,121]. Both the above-mentioned studies, performed in Dutch patients receiving either ABI or ENZ (DTX was allowed in the castration-sensitive stage but not in the CRPC context), as well as a study of Danish patients receiving ABI (previous treatment not stated) [122], demonstrated a shorter PFS in miR-375 high patients. OS was only reported in the latter study and was not significantly different in miR-375-high patients [122]. Mechanistically, overexpression of miR-375 was described in PCa cell lines to be associated with proliferation and metastasis; additionally, it is linked with resistance to both ENZ (by silencing the expression of PTPN4, an inactivator of STAT3) and docetaxel (by silencing SEC23A and YAP1) [159,160].

The third category contains the vast majority of identified biomarkers, which were typically investigated in a single study each, or at most two studies with contradictory results. The list is presented in Table 5.

Finally, the following biomarkers have no supporting evidence: DNA methylation patterns at baseline and SPOP alterations in cfDNA, MYCN alterations in CTCs, and TMPRSS2-ERG fusion in CTCs and whole-blood mRNA. Nonetheless, due to the very limited volume of evidence, these markers, especially DNA methylation and MYCN, should not necessarily be excluded from follow-up studies.

Specifically, it must be mentioned that DNA methylation status at baseline was not significantly associated with outcomes; however, a number of DNA methylation patterns after the beginning of treatment are correlated with survival [126]. This suggests that changes in DNA methylation patterns induced by ARSI treatment could point to adaptations undergone by the PCa cell in response to ARSIs.

MYCN amplification is involved in neuroendocrine differentiation, an important mechanism of ARSI resistance [161]; considering the borderline-significant confidence interval (lower bound 0.9) [80] and the relatively low sample size (69 CTC samples), it is likely that the lack of significance reflects the low statistical power of the study and does not disprove the biological relevance of MYCN amplification.

SPOP mutations were identified in up to 15% of PCa and are believed to be an early event in malignant transformation [162]; more importantly, SPOP is normally involved in the ubiquitination and subsequent degradation of full-length AR, and PCa-specific SPOP mutations are associated with the loss of this function [163]. The lack of prognostic significance can be rationalized as follows: a lack of degradation of an otherwise normal AR only leads to increased AR activation in response to existing androgens, which can be compensated by the effect of ARSI.

Finally, TMPRSS2-ERG fusion is an event that occurs early in malignant transformation; the fusion leads to oncogene *ERG* becoming AR-dependent, via the androgen response element of gene *TMPRSS2* [164]. Similar to SPOP, the inhibition of an otherwise normal AR using ARSIs should nullify the effect of the fusion; in other words, TMPRSS2-ERG fusion is not sufficient for ARSI resistance.

Regarding the practical relevance of the above-mentioned biomarkers, it should be noted that biomarkers in the first category are those with the highest potential for clinical relevance, supported by a large amount of evidence in various populations and clinical contexts; additionally, the markers in this category are relatively frequent in mCRPC patients. On the other hand, markers in the second and third categories are either insufficiently studied (especially the latter category), have produced contradictory results, or both. We consider that these markers are in need of further study, both on a clinical and cellular level, in order to elucidate their potential for predicting ARSI responsiveness, on one hand, and for identifying mechanisms of resistance to ARSIs on the other hand. Three markers are nonetheless particularly promising and should be validated in larger, more targeted studies: cfDNA-level alterations or copy number loss in tumor suppressors PTEN, RB1, and TP53, which were linked to more consistent results. Other strengths of these biomarkers are their relatively high prevalence, as well as an ample body of the literature regarding their molecular mechanisms of action. However, a possible weakness is that the statistical significance of PTEN and RB1 is generally not retained in multivariable analyses.

Despite the significant interest in ncRNA research in recent years, including the regulatory approval of the ncRNA-based PCA3 assay in early prostate cancer [165], our review suggests that ncRNAs are in fact understudied: only one miRNA, miR-375, was evaluated in more than two papers; moreover, of the four identified articles, three were performed by the same team on patients presenting in the same institution, which potentially limits the generalizability of the results. Circular RNAs were likewise evaluated in one study [125] and other epigenetic markers such as SIRT2 overexpression (a proxy for low H3K18 acetylation) [127] and DNA methylation patterns [126].

Adherence to reporting guidelines such as the REMARK checklist [26] for full texts of biomarker studies or the STROBE [28] and CONSORT for abstracts [27] of observational studies and, respectively, of clinical trials was acceptable: just under two thirds of both full texts (56 of 88 reports) and abstracts (23 of 35 reports) respecting at least 75% of the items in their respective checklists (see Supplementary Materials Data S2). The most common deficiencies for full texts were the lack of justification for sample size, not evaluating the relation between the investigated biomarkers and standard prognostic variables, and the lack or incompleteness of multivariable analyses. In abstracts of observational studies, the main deficiencies refer to not mentioning the type of study in the title, not reporting the number of participants at the end of the study, and not stating follow-up duration.

An important vulnerability of liquid biopsy approaches is the severe underrepresentation of non-Western populations: most studies were performed on patients from Australia, the United States, Canada, or Italy; East Asian populations are represented by a few studies from China, Japan, and South Korea, while other populations remain essentially un-investigated. In the same note, despite the fact that African Americans have a higher incidence of prostate cancer and worse outcomes compared to other Americans [166], they were severely underrepresented in all but one study [110]. The lack of published papers can be explained, at least in the case of low and middle-income countries, by a lack of awareness and limited interest in adopting liquid biopsy methods, in addition to financial constraints [167].

One of the main limitations of this review is the lack of a meta-analysis. A meta-analysis would have been mathematically impossible or of very limited use for a majority of identified biomarkers, which were only evaluated in at most two studies. In other cases, the main obstacle is the considerable heterogeneity of the included studies. The same marker might have been evaluated using different assays (e.g., CellSearch^®^ or AdnaTest^®^ for CTCs) or different thresholds (ctDNA fraction, overexpression of various genes), and studies often reported different outcome measures (OS, PFS, TTP, or time to treatment failure; clinical, radiographic, or PSA definition of progression). Nonetheless, meta-analyses have been performed for CTC counts [168] and AR-V7 [169], which further support their usefulness in mCRPC.

Another limitation is that this review did not address whether a biomarker is a predictor of worse outcomes under ARSIs but not taxanes (and could be used to guide treatment) or as a predictor of worse outcomes in general (in which case it would only be useful as a prognostic marker). We did not investigate this direction because of a lack of evidence; nonetheless, this topic can only be studied properly in clinical trials.

## 5. Conclusions

Liquid biopsy approaches based on CTCs, cfDNA, and exosomal, plasma, and whole-blood RNA show great potential in predicting response to ARSI treatment. Nonetheless, despite the rapid growth of the field, most biomarkers are still under-investigated, with either few and small-scale studies or contradictory evidence. The best-studied cfDNA biomarkers are AR-V7, AR copy number gain, and ctDNA fraction; AR-V7, AR gain or overexpression, and CTC counts are the most important CTC biomarkers; AR-V7 and AR overexpression are the most promising markers in exosomal and plasma RNA, and AR-V7 in whole-blood RNA assays. Additionally, of the less-studied biomarkers, the most promising are cfDNA-level alterations or copy number loss in the three tumor suppressors PTEN, RB1, and TP53, which are supported by the most evidence and more consistent results across studies; additionally, the prevalence of each of the three alterations is sufficiently high for practical application. Conversely, a few markers are not supported by available evidence; i.e., all studies until now have obtained negative results. Nonetheless, since they were each evaluated in a single, small-scale study, this assessment could change as further evidence emerges, especially for the first two markers: DNA methylation at baseline, MYCN copy number loss, SPOP alterations, and TMPRSS2-ERG fusion.

Our research also highlighted two important vulnerabilities of liquid biopsy research as a whole. The first issue refers to sample representativeness: specifically, liquid biopsy approaches were only evaluated in Western populations (and to a lesser extent in Chinese and Japanese patients); likewise, African Americans were underrepresented in reports from the United States. This highlights a pressing need for further research in large, international studies that could include patients from diverse populations. The second potential weakness of the field that should be addressed in further studies is the incomplete adherence to standardized reporting guidelines, such as REMARK, and particularly the inconsistent reporting of multivariable analysis, which limits the comparability and generalizability of results. 

## Figures and Tables

**Table 1:** CTC counts and CTC dynamics and their relation to outcomes under ARSI treatment.

Marker	Type	Outcome	Treatment	Previous CRPC Treatment	Population	Prevalence of Alteration	Significant in MV Analysis	Follows Checklist	Ref.
≥1 CTC/7.5 mL blood (CellSearch^®^)	FT	PFS HR 2.51 (1.67–3.75), OS HR 5.02 (2.64–9.55)	ABI/ENZ	Any	Belgium	69%	Yes	Yes	[30]
≥1 CTC/7.5 mL blood (CellSearch^®^)	FT	rPFS HR 1.940 (1.01–3.73), OS HR 2.50 (1.12–5.59)	ABI	NR	China	68.40%	Yes	No	[31]
≥3 CTC/mL blood (CellSearch^®^)	FT	OS HR 2.3 (1.6–3.3) (discovery cohort)	ABI/ENZ	Prior ARSI or taxanes allowed	United States	28.70%	Yes	Yes	[32]
≥3 CTC/mL blood (CellSearch^®^)	FT	PFS HR 2.2 (1.4–3.3) OS HR 2.5 (1.6–3.9) (validation cohort)	ABI/ENZ	Prior ARSI or taxanes allowed	United States	36%	Yes	Yes	[32]
≥5 CTC/7.5 mL blood (CellSearch^®^)	FT	rPFS HR 2.35 (1.14–4.84), OS HR 3.08 (1.45–6.54)	ENZ	Taxanes/ABI	Italy	42.2%	No	Yes	[33]
≥5 CTC/7.5 mL blood (CellSearch^®^)	Abs	median rPFS 6.8 vs. 11 mo., median OS 13.6 mo. vs. not reached	ENZ	Post-DTX	United States	49.50%	NR	Yes	[34]
≥5 CTC/7.5 mL blood (CellSearch^®^)	FT	rPFS HR 1.95 (1.03–3.70), OS HR 2.10 (0.845–5.20)	ABI	NR	China	22.50%	Yes	No	[31]
≥5 CTC/7.5 mL blood (CellSearch^®^)	Abs	lower OS (*p* = 0.001)	ABI	NR	United States	34.30%	NR	Yes	[35]
≥5 CTC/7.5 mL blood (CellSearch^®^)	FT	crPFS HR 2.475 (1.72–3.67), OS HR 4.75 (2.76–8.27)	ABI/ENZ	None/DTX/ARSI	Belgium	40.60%	As continuous variable	Yes	[36]
≥5 CTC/7.5 mL blood (CellSearch^®^)	Abs	rPFS HR 3.8 (*p* = 0.02)	ABI	Prior ChT allowed	Germany	27.6% of all; 40% of CTC+	NR	Yes	[37]
≥5 CTC/7.5 mL blood (CellSearch^®^)	Abs	OS HR 1.92 (0.99–3.7)	ABI	No prior ChT	United States	NR	NR	Yes	[38]
≥5 CTC/7.5 mL blood (CellSearch^®^)	FT	median OS 48 vs. 122 wk. (*p* < 0.001)	ABI	Prior ARSI or ChT allowed	United States	73%	NR	No	[39]
≥5 CTC/7.5 mL blood (CellSearch^®^)	Abs	Not significant	ABI/ENZ	NR	Italy	32%	NR	Yes	[40]
≥5 CTC/7.5 mL blood (CellSearch^®^)	FT	OS HR 3.42 (1.53–7.65)	ABI	None (first-line)	United States	39.10%	No	Yes	[41]
≥5 CTC/7.5 mL blood (CellSearch^®^)	Abs	OS HR 2.97 (*p* = 0.0012)	ABI	NR	United States	39.40%	NR	Yes	[42]
≥5 CTC/7.5 mL blood (CellSearch^®^)	FT	median PFS ~3 mo. vs. not reached	ABI/ENZ	NR	United States	NR	NR	No	[43]
≥5 CTC/7.5 mL blood (CellSearch^®^)	Abs	OS HR 3.42 (*p* = 0.02)	ABI	None (first-line)	United States	NR	NR	Yes	[44]
≥5 CTC/7.5 mL blood (flow cytometry-based assay)	Abs	rPFS HR 2.29 (1.0165–5.145), OS HR 3.06 (1.135–8.27)	ENZ	NR	Italy	NR	NR	Yes	[45]
CTC as a continuous variable (CellSearch^®^ assay)	FT	PFS HR 1.33 (1.14–1.55) (multivariate)	ABI/ENZ	Prior ARSI or ChT allowed	Belgium	NA	Yes	Yes	[46]
CTC+ (AdnaTest^®^, Venlo, The Netherlands)	FT	rPFS HR 5.21 (2.82–9.64), PSA PFS HR 3.37 (2.01–5.56), OS HR 4.68 (2.64–8.28)	ENZ	None	Spain	36%	Yes	Yes	[47]
CTC+ (AdnaTest^®^)	FT	PFS HR 2.37 (0.97–5.71)	ENZ	None/DTX/ARSI	Japan	56.50%	NR	No	[48]
CTC+ (AdnaTest^®^)	FT	rPFS HR 4.38 (1.7–11.3), PSA PFS HR 3.85 (1.64–9.01), OS HR 6.21 (1.38–28.17)	ABI/ENZ	Prior ARSI or ChT allowed	Italy	56.80%	NR	No	[49]
CTC+ (AdnaTest^®^)	FT	median PFS 16 mo. vs. not reached (*p* = 0.02), median OS 29 mo. vs. not reached (*p* = 0.05)	ABI/ENZ	None (first-line)	Italy	43.2%	NR	No	[50]
CTC+ (AdnaTest^®^)	FT	OS HR 3.03 (1.11–8.23)	ENZ	Prior ABI or DTX allowed	Japan	60.50%	NR	Yes	[51]
CTC+ (Dynabeads^®^)	FT	PFS HR 3.97 (1.52–10.37), OS HR 5.24 (1.86–14.81)	ABI/ENZ	Prior ARSI or ChT allowed	Germany	73.70%	NR	No	[52]
CTC+ (Epic Sciences^®^, San Diego, CA, USA)	Abs	OS HR 1.58 (0.31–7.90) (multivariate)	ABI/ENZ	NR	United States	69%	NA	Yes	[29]
CTC conversion from ≥5/7.5 mL to <−5/7.5 m at week 12	FT	OS HR 3.76 (*p* < 0.001)	ABI	Post-DTX	Multicenter	18.1%	Yes	Yes	[68]
CTC conversion (≥5 to <5/7.5 mL) at week 9 (CellSearch^®^)	Abs	rPFS HR 0.16 (0.07–0.40)	ENZ	None (first-line)	Multicenter	NR	NR	Yes	[69]
CTC conversion from (≥5 to <5/7.5 mL) at week 17 (CellSearch^®^)	Abs	rPFS HR 0.26 (0.11–0.59)	ENZ	None (first-line)	Multicenter	NR	NR	Yes	[69]
CTC negativation (>0 to 0) at week 9 (CellSearch^®^)	Abs	rPFS HR 0.41 (0.24–0.69)	ENZ	None (first-line)	Multicenter	NR	NR	Yes	[69]
CTC negativation (>0 to 0) at week 17 (CellSearch^®^)	Abs	rPFS HR 0.36 (0.20–0.64)	ENZ	None (first-line)	Multicenter	NR	NR	Yes	[69]
No CTC negativation during treatment (AdnaTest^®^)	FT	OS HR 3.97 (1.36–11.67)	ENZ	Prior ABI or DTX allowed	Japan	46.2% of initially CTC+	Yes	Yes	[51]

Note: Results are presented as hazard ratio (95% confidence interval) unless otherwise specified. Abbreviations: ABI, abiraterone; Abs, abstract; ARSI, androgen receptor signaling inhibitor; ChT, chemotherapy; CTC, circulating tumor cell; DTX, docetaxel; ENZ, enzalutamide; FT, full text; HR, hazard ratio; mo., month; MV, multivariable; NA, not applicable; NR, not reported; OS, overall survival; (c, r)PFS, (clinical, radiographic) progression-free survival; Ref., reference; and wk., week.

**Table 2:** ctDNA fraction and cfDNA concentration and their relation to outcomes under ARSI treatment.

Marker	Type	Outcome	Treatment	Previous CRPC Treatment	Population/Region	Prevalence of Alteration	Significant in MV Analysis	Follows Checklist	Ref.
ctDNA fraction > 18.0%	FT	PSA PFS HR 4.64 (1.53–14.06), OS HR 3.50 (1.14–10.77) (MV)	ABI/ENZ	Prior DTX allowed	Italy	50% (median as threshold)	NA	No	[54]
ctDNA fraction high	Abs	OS HR 2.5 (*p* < 0.001)	APA + ABI	NR	Multicenter	63%	Yes	Yes	[55]
ctDNA fraction > 2%	FT	TTP HR 3.56 (2.28–5.57), OS HR 12.92 (5.68–29.42)	ABI/ENZ	None (first line)	Canada	20.9%	NR	Yes	[56]
ctDNA fraction > 30%	FT	TTP HR 2.05 (1.42–2.96), OS HR 7.51 (3.41–16.57)	ABI/ENZ	None (first line)	Canada	57.2%	Yes	Yes	[56]
ctDNA fraction > 18.8%	FT	PFS HR 1.91 (1.13–3.21), OS HR 2.34 (1.32–4.12) (MV)	ABI/ENZ	Prior ARSIs or taxanes allowed	Italy	50% (median as threshold)	NA	Yes	[57]
ctDNA fraction > 1%	FT	PFS HR 2.48 (1.45–4.23), OS HR 3.56 (1.89–6.72)	ABI/ENZ	No prior ChT	Netherlands	59.3%	NR	Yes	[58]
ctDNA fraction > 30%	FT	PFS HR 6.26 (3.03–12.94), OS HR 8.12 (3.75–17.61)	ABI/ENZ	No prior ChT	Netherlands	19.8%	NR	Yes	[58]
ctDNA mutant allele fraction > 7%	FT	PFS HR 1.76 (1.03–3.01), OS HR 2.92 (1.4–6.11)	ABI/ENZ	Prior ChT or ARSI allowed	United States	43.50%	NR	Yes	[59]
ctDNA fraction high	FT	OS HR 1.04 (1.01–1.07)	ABI	None (first-line)	United States	NR	NR	Yes	[60]
ctDNA genomic complexity high (equivalent to >10% ctDNA)	FT	PFS HR 5.59 (1.03–30.28)	ABI/ENZ	Prior DTX or ARSI allowed	Austria + United States	40%	NR	Yes	[61]
ctDNA fraction ≥2%	Abs	TTPP HR 2.04 (1.43–2.90), OS HR 4.07 (2.40–6.91)	ABI/ENZ	NR	Canada	NR	NR	No	[62]
ctDNA fraction ≥18.1%	FT	rPFS HR 1.83 (1.15–2.94), OS HR 2.23 (1.21–4.09)	ABI	Prior ENZ/orteronel/taxanes allowed	Italy + United Kingdom	50% (median as threshold)	Yes	Yes	[63]
ctDNA positive	FT	rPFS HR 1.8 (1.20–2.96), OS HR 3.01 (1.88–4.83)	ABI	NR	Multicenter	46.9%	Yes	Yes	[64]
ctDNA fraction 3.2–21.1% relative to <3.2%	FT	PSA PFS HR 1.80 (1.13–2.87), OS HR 1.94 (1.07–3.52)	ABI/ENZ	Prior DTX in castration-sensitive context allowed	Denmark	NR	Yes	Yes	[53]
ctDNA fraction >21.1% relative to <3.2%	FT	PSA PFS HR 4.075 (2.50–6.65), OS HR 5.08 (2.77–9.31)	ABI/ENZ	Prior DTX in castration-sensitive context allowed	Denmark	NR	Yes	Yes	[53]
cfDNA concentration > 3.4 ng/mL	FT	median OS ~19 mo. vs. not reached (*p* = 0.01)	ABI/ENZ	None (first line)	Netherlands	50% (median as threshold)	NR	No	[66]
cfDNA concentration > 38.5 ng/mL	FT	OS HR 4.24 (2.64–6.79)	ABI/ENZ	Prior DTX allowed	Italy	50.50%	NR	Yes	[67]
cfDNA concentration > 8.4 ng/mL	FT	PSA PFS HR 2.0 (1.1–3.5), crPFS HR 2.4 (1.3–4.2), OS HR 3.3 (1.7–6.6)	ABI/ENZ	Prior ARSI/ChT allowed	Australia	50% (median as threshold)	Yes	No	[65]

Note: Results are presented as hazard ratio (95% confidence interval) unless otherwise specified. Abbreviations: ABI, abiraterone; Abs, abstract; APA, apalutamide; ARSI, androgen receptor signaling inhibitor; ChT, chemotherapy; cfDNA, cell-free DNA; ctDNA, circulating tumor DNA; DTX, docetaxel; ENZ, enzalutamide; FT, full text; HR, hazard ratio; mo., months; MV, multivariable; NA, not applicable; NR, not reported; OS, overall survival; (c, r)PFS, (clinical, radiographic) progression-free survival; Ref., reference; and TT(C, R, P)P, time to (clinical, radiographic, PSA) progression.

**Table 3:** Presence of AR-V7 and its relation to outcomes under ARSI treatment.

Article Type	Source	Outcome	Treatment	Previous CRPC Treatment	Population/Region	Prevalence of Alteration	Significant in MV Analysis	Follows Checklist	Ref.
FT	CTC	rPFS 7.4 mo. vs. 14.6 mo. PSA-PFS 8.2 mo. vs. 12.6 mo., OS 25.8 mo. vs. 25.4 mo.	ENZ	None (first-line)	Spain	16% of CTC+ (5.8% of total)	NR	Yes	[47]
FT	CTC	median crPFS 4.3 mo. vs. 6.1 mo. (*p* = 0.11)	BAT + ABI/ENZ	ABI/ENZ	United States	19.2%	NR	No	[72]
FT	CTC	Worse PFS (*p* = 0.005), Worse OS (*p* = 0.0055)	ENZ	None (first-line)	Italy	24%	NR	No	[73]
FT	Exosomal RNA	median PFS 5.4 mo. vs. 24.3 mo. (*p* < 0.0001), median OS 16.2 mo. vs. not reached (*p* < 0.0001)	ABI/ENZ	None (first-line)	Italy	36%	Yes	No	[74]
FT	Exosomal RNA	median PFS 4 mo. vs 24 mo. (*p* < 0.0001), median OS 9 mo. vs. not reached (*p* < 0.0001)	ABI/ENZ	Prior taxanes allowed, no prior ARSI	Italy	22%	NR	No	[75]
Abs	CTC	PSA PFS HR 7.4 (2.7–20.6)	ENZ	NR	United States	38.7%	Yes	Yes	[76]
FT	CTC	PSA PFS HR 7.4 (2.7–20.6), crPFS HR 8.5 (2.8–25.5), OS HR 6.9 (1.7–28.1)	ENZ	Prior taxanes or ARSI allowed	United States	39%	PSA PFS and crPFS	Yes	[12]
FT	CTC	PSA PFS HR 6.1 (3.9–66.0) crPFS HR 16.5 (3.3–82.9), OS HR 12.7 (1.3–125.3)	ABI	Prior taxanes or ARSI allowed	United States	19%	PSA PFS and crPFS	Yes	[12]
FT	Whole-blood mRNA	median PSA PFS 2.4 mo. vs. 3.7 mo. (*p* < 0.001), median cPFS 2.7 mo. vs. 5.5 mo. (*p* < 0.001), median OS 4 mo. vs. 13.9 mo. (*p* < 0.001)	ABI/ENZ	Prior DTX, CBZ, or ABI allowed	Germany	18%	Yes	No	[77]
FT	Whole-blood mRNA	PSA PFS HR 8.8 (2.3–29.8), OS HR 6.8 (1.8–22.0)	ABI	Prior DTX or CBZ allowed	Canada	11.1%	Yes	No	[78]
FT	CTC	rPFS HR 5.05 (2.4–10.64), OS HR 2.25 (1.1–4.58)	ENZ	Prior taxanes or ABI allowed	Italy	48.9%	Yes	Yes	[33]
FT	Whole-blood mRNA	TTF HR 1.73 (0.83–3.60) (3rd vs. 1st and 2nd tertile) (MV)	ABI	Prior taxanes or ABI allowed	United States	33%	NA	Yes	[79]
FT	Whole-blood mRNA	TTF HR 2.08 (0.83–5.24) (3rd vs. 1st and 2nd tertile) (MV)	ENZ	Prior taxanes or ABI allowed	United States	33%	NA	Yes	[79]
FT	cfDNA	rPFS HR 3.2 (1.4–7.4)	ABI/ENZ	Prior ABI/ENZ allowed	United States	18% of pts. with AR-V7-negative CTCs	NR	Yes	[80]
FT	CTC (in-house assay)	rPFS HR 2.3 (1.5–3.5), OS HR 2.8 (1.7–4.5)	ABI/ENZ	Prior ABI/ENZ allowed	United States	24%	Yes	Yes	[81]
FT	CTC (Epic Sciences^®^ assay, San Diego, CA, USA)	rPFS HR 2.2 (1.2–4.3), OS HR 3.1 (1.6–5.9)	ABI/ENZ	Prior ABI/ENZ allowed	United States	10%	Yes	Yes	[81]
FT	CTC	PFS HR 8.56 (2.40–30.43)	ENZ	None/DTX/ARSI	Japan	8.7% (15.4% of CTC+)	NR	No	[48]
FT	CTC	median PSA PFS ~55 vs. ~145 days (*p* = 0.011), median crPFS ~30 vs. ~200 days (*p* = 0.004)	ABI/ENZ	NR	United States	NR	NR	No	[82]
FT	cfDNA and cfRNA	PSA PFS HR 2.1 (0.81–5.9), crPFS HR 2.4 (0.82–6.9), OS HR 3.5 (1.1–11)	ABI/ENZ	Prior ARSI or ChT allowed	Australia	16.4%	No	Yes	[83]
Abs	Exosomal RNA	PSA PFS HR 4.08 (1.13–14.69)	ABI	NR	China	39.1%	NR	No	[84]
Abs	CTC	PSA PFS 1.00 (0.24–4.18)	ABI	NR	China	36.4%	NR	No	[84]
FT	CTC	crPFS HR 2.685 (1.44–5.02), OS HR 2.95 (1.63–5.345)	ABI/ENZ	Prior ARSI or ChT allowed	Germany	50.8% (61.1% of CTC+)	No	Yes	[85]
FT	CTC	rPFS HR 4.00 (1.67–9.41), PSA PFS HR 2.98 (1.34–6.61), OS HR 11.1 (2.43–50.63)	ABI/ENZ	Prior ARSI or ChT allowed	Italy	45.9%	NR	No	[49]
FT	CTC (conventional AdnaTest^®^)	rPFS HR 2.1 (0.6–7), PSA PFS HR 3.1 (1.0–9.2)	ABI/ENZ	Prior ChT/ARSI/radium allowed	France	22%	Only PSA PFS	No	[86]
FT	CTC (highly sensitive assay)	rPFS HR 7.3 (1.6–33), PSA PFS HR 10.8 (2.4–48.2)	ABI/ENZ	Prior ChT/ARSI/radium allowed	France	56.1%	Yes	No	[86]
Abs	CTC	PSA PFS HR 4.35 (1.27–14.91), crPFS HR 5.43 (1.52–19.35)	ABI/ENZ	NR	United States	NR	NR	Yes	[87]
FT	Plasma RNA	PFS HR 1.37 (0.53–3.57)	ABI	Prior DTX allowed	Netherlands	11.3%	NR	Yes	[88]
FT	CTC	PFS HR 4.27 (1.77–10.27)	ABI/ENZ/APA	Prior ARSI/taxanes allowed	United States	NR	Yes	Yes	[89]
FT	Exosomal RNA	median PFS 16.0 vs. 28.0 mo. (*p* = 0.049), median OS 22.9 vs. 32.9 mo. (*p* = 0.111)	ABI/ENZ	None (first-line)	Canada	34.3%	NR	No	[90]
FT	CTC	median PFS 13 mo. vs. 16 mo. (*p* = 0.89), median OS NR vs. 29 mo.(*p* = 0.33) (relative to CTC+/AR-V7-)	ABI/ENZ	None (first-line)	Italy	6.8%	NR	No	[50]
Abs	CTC	PFS log-rank *p* = 0.055, OS log-rank *p* = 0.02	ABI/ENZ	NR	Italy	14.3%	NR	Yes	[40]
FT	CTC	median rPFS ~200 vs. ~430 days (*p* = 0.037), median PSA PFS ~130 vs. ~455 days (*p* < 0.0001), median cPFS ~215 zs ~425 days (*p* = 0.012), median cancer-specific survival ~1085 vs. ~1585 days (*p* = 0.474)	ABI	None (first-line)	China	35.8%	Yes	Yes	[91]
FT	CTC	median PFS 3 mo. vs. 20 mo. (*p* < 0.001), median OS 8 mo. vs. not reached (*p* < 0.001)	ABI/ENZ	Prior ABI or taxanes allowed	Italy	38.9%	No	Yes	[71]

Note: Results are presented as hazard ratio (95% confidence interval) unless otherwise specified. Abbreviations: ABI, abiraterone; Abs, abstract; APA, apalutamide; BAT, bipolar androgen therapy; CBZ, cabazitaxel; cfDNA, cell-free DNA; cfRNA, cell-free RNA; ChT, chemotherapy; ENZ, enzalutamide; HR, hazard ratio; mo., months; MV, multivariable; NA, not applicable; NR, not reported; OS, overall survival; (c, r)PFS, (clinical, radiographic) progression-free survival; pts., patients/participants; Ref., reference; and TTF, time to treatment failure.

**Table 4:** AR copy number gain or AR overexpression and their relation to outcomes under ARSI treatment.

Marker	Article Type	Source	Outcome	Treatment	Previous CRPC Treatment	Population/Region	Prevalence of Alteration	Significant in MV Analysis	Follows Checklist	Ref.
AR gain	FT	cfDNA	rPFS HR 9.83 (4.5–21.44), PSA PFS HR 4.03 (1.87–8.72), OS HR 6.65 (3.18–13.91)	ENZ	None (first line)	Spain	11%	Yes	Yes	[47]
	FT	cfDNA	median PFS 4.8 mo. vs. 24.3 mo. (*p* < 0.0001), median OS 8.17 mo. vs. not reached (*p* < 0.0001)	ABI/ENZ	None	Italy	14%	Yes	No	[74]
	FT	cfDNA	PFS HR 2.18 (1.08–4.39), OS HR 3.98 (1.74–9.10)	ABI/ENZ	No prior taxanes	Italy + United Kingdom	NR	Yes	Yes	[92]
	FT	cfDNA	PFS HR 1.95 (1.23–3.11) OS HR 3.81 (2.28–6.37)	ABI/ENZ	Post-DTX	Italy + United Kingdom	NR	Yes	Yes	[92]
	FT	CTC	rPFS HR 1.6 (0.8–3.2)	ABI/ENZ	Prior ABI/ENZ allowed	United States	45%	NR	Yes	[80]
	FT	cfDNA	PSA PFS HR 1.72 (1.05–2.81), OS HR 1.44 (0.86–2.40) (multivariate)	ABI/ENZ	Prior DTX allowed	Italy	33%	NA	No	[54]
	FT	cfDNA	median PFS 5.4 mo. vs. 9.1 mo. (*p* = 0.0005), median OS 9.1 mo. vs. 27.3 mo. (*p* < 0.0001)	ABI/ENZ	Pre- or post-DTX	Italy	21%	NR	No	[93]
	FT	cfDNA	PFS HR 2.68 (1.49–4.82), OS HR 2.09 (1.07–4.10) (MV)	ABI/ENZ	Post-DTX	Italy	30%	NA	Yes	[94]
	FT	cfDNA	PFS HR 2.79 (1.55−5.02), OS HR 3.23 (1.64−6.35)	ENZ	DTX in all, ABI in 48%	Italy	36%	Yes	Yes	[95]
	FT	cfDNA	PFS HR 3.73 (1.95–7.13), OS HR 4.68 (2.17–10.10)	ABI	Post-DTX	Italy	30%	Yes	Yes	[96]
	FT	CTC	median PSA PFS ~70 vs. ~400 days (*p* = 0.006), median crPFS ~100 vs. ~655 days (*p* = 0.029)	ABI/ENZ	NR	United States	NR	NR	No	[82]
	Abs	cfDNA	lower OS (*p* < 0.0001)	ABI	NR	United States	27%	NR	Yes	[35]
	FT	cfDNA	Any CN gain: TTP HR 2.05 (1.43–2.93), CN ≥ 8: TTP HR 2.65 (1.68–4.19), CN < 8: TTP HR 1.67 (1.07–2.62)	ABI/ENZ	None (first line)	Canada	Any CN: 33.2%, CN ≥ 8: 15.3%, CN < 8: 17.8%	No	Yes	[56]
	FT	cfDNA	PSA PFS HR 2.8 (1.3–6.1), crPFS HR 3.4 (1.4–8.2), OS HR 3.2 (1.2–8.5)	ABI/ENZ	Prior ARSI or ChT allowed	Australia	39%	Yes	Yes	[83]
	FT	cfDNA	PFS HR 2.07 (1.2–3.57), OS HR 3.26 (1.52–7)	ABI/ENZ	Prior ARSI or ChT allowed	United States	52%	No	Yes	[59]
	Abs	cfDNA	PFS HR 2.92 (1.59–5.37)	ENZ	NR	Canada	NR	NR	No	[97]
	FT	cfDNA	OS HR 1.56 (0.99–2.46)	ABI	None (first-line)	United States	NR	No	Yes	[60]
	FT	cfDNA	PFS HR 2.2 (1.2–4.4), OS HR 2.9 (1.5–5.7)	ABI/ENZ	Prior ARSI or ChT allowed	Australia	22%	Only PFS	No	[98]
	FT	cfDNA	OS HR 2.64 (1.60–4.35)	ABI/ENZ	Prior DTX allowed	Italy	26%	Yes	Yes	[67]
	FT	cfDNA	rPFS HR 2.0 (0.97–4.405), cPFS HR 1.95 (0.897–3.87), OS HR 2.37 (1.07–5.25) (MV)	ABI/ENZ	None (first-line)	Multicenter	17%	NA	Yes	[99]
	FT	cfDNA	rPFS HR 3.52 (2.02–6.13), OS HR 7.09 (3.34–15.05)	ABI	Prior ENZ/orteronel/taxanes allowed	Italy + United Kingdom	37%	Yes	Yes	[63]
	FT	cfDNA	cPFS HR 2.5 (1.3–4.8), OS HR 1.9 (0.99–3.8)	ABI/ENZ	Prior ARSI or ChT allowed	Australia	42%	Yes	Yes	[100]
	FT	cfDNA	rPFS HR 1.98 (1.23–3.18), OS HR 1.96 (1.17–3.29)	ABI	NR	Multicenter	21.1%	No	Yes	[64]
	FT	cfDNA	PFS HR 6.67 (1.52–33.33), OS HR 8.33 (0.96–100)	ABI	Prior treatment allowed	Belgium	NR	Yes	Yes	[101]
	FT	cfDNA	PFS HR 4.76 (1.41–16.67) OS HR 4.55 (0.94–20)	ENZ	Prior treatment allowed	Belgium	NR	Yes	Yes	[101]
	FT	cfDNA	OS HR 5.25 (2.21–12.46)	ABI	None (first-line)	United States	37%	No	Yes	[41]
	FT	cfDNA	median PFS 5.3 mo. vs. 9 mo.(*p* = 0.0001)	ABI	Prior DTX	Italy	38.9%	Yes	Yes	[102]
	FT	cfDNA	median PFS 2.8 mo. vs. 4.9 mo. (*p* = 0.0001)	ENZ	Prior DTX	Italy	32.40%	Yes	Yes	[102]
	FT	cfDNA	PFS HR 3.91 (1.29–11.28)	ABI	Prior treatment allowed	China	9%	NR	No	[103]
AR over-expression	Abs	CTC	PSA PFS HR 3.61 (1.06–12.29), crPFS HR 4.91 (1.38–17.48)	ABI/ENZ	NR	United States	NR	NR	Yes	[87]
	FT	CTC	median PFS 16 mo. vs. 13 mo. (*p* = 0.99), median OS 29 mo. vs. not reached (*p* = 0.72)	ABI/ENZ	None (first-line)	Italy	27.3%	NR	No	[50]
	Abs	CTC	AR-FL negative vs. below median vs. above median: median PSA PFS 9.6 vs. 6.2 vs. 2.5 mo., median cPFS 11.1 vs. 8.7 vs. 3.2 mo., median OS 33.3 vs. 18.0 vs. 11.3 mo. (all *p* < 0.001) (multivariate)	ABI/ENZ	NR	United States	AR-FL positive: 52%	NA	Yes	[104]
	FT	Exosomal RNA	AR high vs. intermediate vs. low: median PFS 4 mo. vs. 18 mo. vs. 22 mo. (*p* = 0.014)	ABI/ENZ	Prior taxanes allowed, no prior ARSI	Italy	NR	NR	No	[75]

Note: Results are presented as hazard ratio (95% confidence interval) unless otherwise specified. Abbreviations: ABI, abiraterone; Abs, abstract; AR, androgen receptor; AR-FL, full-length AR; ChT, chemotherapy; cfDNA, cell-free DNA; CTC, circulating tumor cells; DTX, docetaxel; ENZ, enzalutamide; HR, hazard ratio; mo., months; MV, multivariable; NA, not applicable; NR, not reported; OS, overall survival; (c, r)PFS, (clinical, radiographic) progression-free survival; Ref., reference; and TTP, time to progression.

**Table 5:** The classification of identified biomarkers of ARSI response.

Source	Best Studied Biomarkers	Relatively Well-Studied Biomarkers	Understudied Biomarkers or Contradictory Evidence	Biomarkers not Supported by Current Evidence
cfDNA	AR-V7, AR gain, ctDNA fraction	More promising: PTEN alteration/loss, RB1 alteration/loss, TP53 alteration/loss Less studied or conflicting evidence: cfDNA concentration, AR point mutations, unspecified AR alterations, PIK3CA gain, PI3K pathway alterations, WNT pathway alterations, genome-wide aneuploidy	APC, BRAF, BRCA2 gain, BRCA2 alteration, CDK12, CYP17A1, MET, MYC, NCOA2, NCOR1, NF1, NKX3-1, OPHN1, ZFHX3, GSTP1, AR or enhancer alterations, AR or BRAF alteration, AR plus NCOA2 alteration, AR-V7 or V9, BRCA2 or ATM alterations, SPOP or CDH1 alteration, DDR pathway alterations, HRR pathway alterations, chr8 and 13q deletion, chr7 and 9q amplification, No. of driver gene mutations/no. of pathways affected	SPOP, DNA methylation patterns at baseline
CTC	AR-V7, AR gain or overexpression, CTC counts	Nuclear localized AR-V7, unspecified AR-V PTEN alteration/loss KLK3 (PSA), PSMA	ANXA2, BMP7, GAS6, GR, HER2, HOXB13, NKX3-1, OCT4, PSCA, RUNX2, SOX2, SPINK1, SYP, TSPAN8, WNT5B, CTC clusters/AR-V7 high, KLK3 high/AR-V7 high, PSA+/PSMA+ high, chromosomal instability phenotype, phenotypic heterogeneity	MYCN, TMPRSS2-ERG fusion
Exosomal or plasma RNA	AR-V7, AR overexpression	Unspecified AR alterations, KLK3, miR-375	AKR1C3, hsa_circ_0113177, 0127731, 0002048, 0097211, 0116020, 0002910 miR-21, -103a, -141, -221, -223, -3687 NAALADL2-AS2, PD-L1, TUBB3, AR-V7 or V9, tdEV concentration	
Whole-blood RNA	AR-V7	KLK3	AR-V12 or V14, FOXA1, GRHL2, HOXB13, KLK2	TMPRSS2-ERG fusion

Note: Unless stated otherwise, biomarkers are listed in the following order: individual genes, multiple genes or pathways, large (cytogenetics-level) alterations, genome-wide alterations, nonspecific markers, and then in alphabetical order. Abbreviations (other than gene/gene product names): AR-V, AR splice variant; cfDNA, cell-free DNA; chr, chromosome; CTC, circulating tumor cells; ctDNA, circulating tumor DNA; DDR, DNA damage response pathway; HRR, homologous recombination repair pathway; and tdEV, tumor-derived extracellular vesicles.

## Data Availability

The tables containing the identified articles at each stage of the literature review process are available upon request from the authors.

## Supplementary Materials

The following supporting information can be downloaded at https://www.mdpi.com/article/10.3390/cancers17152482/s1, Data S1. Database Queries. Data S2. Quality assessment using the REMARK, STROBE, and CONSORT checklists. Table S3.1. AR SNVs as prognostic biomarkers for treatment with second-generation ARSIs. Table S3.2. Other AR-related biomarkers and their relation to outcomes under treatment with second-generation ARSIs. Table S3.3. TP53 alterations as biomarkers of response to ARSI treatment. Table S3.4. DDR or HRR pathway alterations (other than TP53) and their relation to outcomes under ARSI treatment. Table S3.5. RB1 alterations and their relation to outcomes under ARSI treatment. Table S3.6. PTEN/PI3K pathway alterations and their relation to outcomes under ARSI treatment. Table S3.7. WNT pathway alterations and their relation to outcomes under ARSI treatment. Table S3.8. PSA, PSMA, and PSCA and their relation to outcomes under ARSI treatment.
