# Supplementary material for: Liquid Biopsy Biomarkers in Metastatic Castration-Resistant Prostate Cancer Treated with Second-Generation Antiandrogens: Ready for Clinical Practice? A Systematic Review
Source: Cancers (Basel). 2025 Jul 27;17(15):2482. doi: 10.3390/cancers17152482 (PMC12345691; doi:10.3390/cancers17152482)
Supplement: Supplementary file 1 [file cancers-17-02482-s001.zip › Supplementary Data 1.pdf]

Supplementary Data 1. Database Queries

The queries used for database interrogation are as follows.

1. Embase

('prostate cancer'/exp OR 'prostat* cancer' OR 'metastatic prostate cancer'/exp OR 'metastatic prostat* cancer' OR 'castration resistant prostat* cancer'/exp OR 'castration resistant prostate cancer' OR 'prostatic carcinoma' OR 'prostatic adenocarcinoma' OR (prostat* NEAR/5 cancer) OR (prostat* NEAR/5 carcinoma) OR (prostat* NEAR/5 adenocarcinoma)) AND ('liquid biopsy'/exp OR 'liquid biops*' OR 'circulating tumor dna'/exp OR 'circulating tumo* dna' OR 'cell free nucleic acid'/exp OR 'cell free nucleic acid$' OR 'cfdna' OR 'ctdna' OR 'circulating tumor cell'/exp OR 'circulating tumor cell' OR 'exosome'/exp OR 'exosome') AND ('antiandrogen'/exp OR 'antiandrogen' OR 'antiandrogens' OR 'androgen antagonist$' OR 'androgen receptor antagonist$' OR 'abiraterone'/exp OR 'abiraterone*' OR 'abiraterone acetate'/exp OR 'enzalutamide'/exp OR 'enzalutamide' OR 'apalutamide'/exp OR 'apalutamide' OR 'flutamide'/exp OR 'flutamide' OR 'nilutamide'/exp OR 'nilutamide' OR 'bicalutamide'/exp OR 'bicalutamide' OR 'darolutamide' OR 'darolutamide'/exp)

2. PubMed/Medline

((prostat* cancer*) OR "Prostatic Neoplasms"[Mesh] OR (metastatic prostate cancer) OR (castration resistant prostate cancer) OR "Prostatic Neoplasms, Castration-Resistant"[Mesh] OR (prostat* carcinoma*) OR (prostat* adenocarcinoma*)) AND((liquid biops*) OR "Liquid Biopsy"[Mesh] OR (circulating tumo* DNA) OR ctDNA OR "Circulating Tumor DNA"[Mesh] OR (cell free DNA) OR cfDNA OR "Cell-Free Nucleic Acids"[Mesh] OR (circulating tumo* cells) OR "Neoplastic Cells, Circulating"[Mesh] OR (extracellular vesicle*) OR "Extracellular Vesicles"[Mesh] OR exosom* OR "Exosomes"[Mesh]) AND (antiandrogen* OR (androgen receptor antagonist*) OR (androgen antagonist*) OR "Androgen Antagonists"[Mesh] OR abiraterone* OR (abiraterone acetate) OR "Abiraterone Acetate"[Mesh] OR enzalutamide OR apalutamide OR flutamide OR nilutamide OR bicalutamide OR darolutamide)

3. Scopus

TITLE-ABS-KEY (("prostat* cancer" OR "prostat* *carcinoma" OR "prostat* W/5 cancer" OR "prostat* W/5 *carcinoma" OR "metastatic prostat*" OR "castration resistant prostat*") AND ("liquid biops*" OR "circulating tumo* DNA" OR ctDNA OR "circulating tumo* cell*" OR "cell free DNA" OR cfDNA OR "cell free nucleic acid*" OR "exosom*" OR "extracellular vesicle*") AND(antiandrogen* OR "androgen w/5 antagonist*" OR abiraterone OR enzalutamide OR apalutamide OR flutamide OR nilutamide OR bicalutamide OR darolutamide))

4. Web of Science

ALL=(((prostat* cancer*) OR (metastatic prostate cancer) OR (castration resistant prostate cancer) OR (prostat* carcinoma*) OR (prostat* adenocarcinoma*)) AND ((liquid biops*) OR (circulating tumo* DNA) OR ctDNA OR (cell free DNA) OR cfDNA OR (circulating tumo* cells) OR (extracellular vesicle*) OR exosom*) AND (antiandrogen* OR (androgen receptor antagonist*) OR (androgen antagonist*) OR abiraterone* OR (abiraterone acetate) OR enzalutamide OR apalutamide OR flutamide OR nilutamide OR bicalutamide OR darolutamide))
