# Supplementary material for: Liquid Biopsy Biomarkers in Metastatic Castration-Resistant Prostate Cancer Treated with Second-Generation Antiandrogens: Ready for Clinical Practice? A Systematic Review
Source: Cancers (Basel). 2025 Jul 27;17(15):2482. doi: 10.3390/cancers17152482 (PMC12345691; doi:10.3390/cancers17152482)
Supplement: Supplementary file 1 [file cancers-17-02482-s001.zip › Supplementary Data 2.pdf]

Supplementary Data 2. Quality assessment using the REMARK, STROBE and CONSORT checklists

Table S1. Quality assessment for full-texts (REMARK checklist)

Reference	(1)	(2)	(3)	(4)	(5)	(6)	(7)	(8)	(9)	(10)	(11)	(12)	(13)	(14)	(15)	(16)	(17)	(18)	(19)	(20)
Study	Fernandez-Perez et al. 2023	Markowski et al. 2021	Peter et al. 2022	Sepe et al. 2024	Del Re et al. 2021	Del Re et al. 2019	Conteduca et al. 2017 A	Miyamoto et al. 2012	Antonarakis et al. 2014 A	Okegawa et al. 2018	Seitz et al. 2017	Erb et al. 2020	Todenhöfer et al. 2017	Scher et al. 2018	Di Lorenzo et al. 2021	Zedan et al. 2020	Qu et al. 2017	Scher et al. 2016	Brown et al. 2021	Gupta et al. 2020
Year	2023	2021	2022	2024	2021	2019	2017	2012	2014	2018	2017	2020	2017	2018	2021	2020	2017	2016	2021	2020
1. Marker, objectives, pre-specified hypotheses	1	1	1	1	1	1	1	1	1	1	1	1	1	1	1	1	1	1	1	1
2. Characteristics of patients	1	1	1	1	1	1	1	1	1	1	1	1	1	1	1	1	1	1	1	1
3. Treatments	1	1	1	1	1	1	1	0	1	1	1	1	1	1	1	0	1	1	1	1
4. Biological material	1	0	1	1	1	1	1	1	1	1	1	1	1	1	1	1	1	1	1	1
5. Assay, protocol, blinding	1	1	1	1	1	1	1	1	1	1	1	1	1	1	1	1	1	1	1	1
6. Case selection, time period, end of follow-up, median follow-up	1	1	0	1	0	0	1	1	1	1	0	0	0	1	1	1	1	1	1	0
7. Define all clinical endpoints	1	1	1	1	1	1	1	1	1	1	1	1	1	1	1	1	1	1	1	1
8. List all candidate variables	0	0	0	0	1	1	1	0	1	1	1	0	0	1	0	0	1	1	1	0
9. Explain sample size / effect size	0	1	0	1	0	0	0	0	1	0	0	0	0	0	0	0	0	0	0	0
10. Specify all statistical methods, variable selection, assumptions, missing data	1	1	1	0	1	1	1	1	1	1	1	1	1	1	1	1	1	1	1	1
11. Clarify how marker values were handled, cutpoint determination	1	1	1	1	1	1	1	1	1	1	1	1	1	1	1	1	1	1	1	1
12. Flow of patients	0	1	0	1	0	0	1	0	1	0	0	0	0	1	0	1	0	0	1	1
13. Demographic characteristics	1	1	0	1	1	1	1	0	1	1	1	1	1	1	1	1	1	1	1	1

prognostic variables, missing values.																				
14. Relation of marker to standard prognostic variables	1	0	0	0	0	0	0	0	1	1	0	0	0	0	0	0	0	0	1	1
15. Univariable analyses	1	1	1	1	1	1	1	1	1	1	1	1	1	1	1	1	1	1	1	1
16. Key multivariable analyses: report estimated effect. For final model, all other variables in the model	1	0	1	0	1	0	1	0	1	1	1	0	0	0	1	0	1	1	1	0
17. Estimated effects with confidence intervals from an analysis with marker and standard prognostic variable	1	0	1	0	0	0	0	0	0	1	1	0	1	0	0	0	0	1	0	0
18. Further investigations (assumptions, sensitivity analyses, internal validation)	1	0	1	0	0	0	0	1	1	0	1	0	0	0	1	1	1	0	0	1
19. Interpret results in context of pre-specified hypotheses and other studies; include limitations	1	1	1	1	1	1	1	1	1	1	1	1	1	1	1	1	1	1	1	1
20. Implications for future research	1	1	1	1	1	1	1	1	1	1	1	1	1	1	1	1	1	1	1	1
Total	17	14	14	14	14	13	16	12	19	17	16	12	13	15	15	14	16	16	17	15
Follows REMARK checklist	Yes	No	No	No	No	No	Yes	No	Yes	Yes	Yes	No	No	Yes	Yes	No	Yes	Yes	Yes	Yes

Reference	(21)	(22)	(23)	(24)	(25)	(26)	(27)	(28)	(29)	(30)	(31)	(32)	(33)	(34)	(35)	(36)	(37)	(38)	(39)	(40)
Study	Armstrong et al. 2020	Halabi et al. 2024 A	Gupta et al. 2023	Oeyen et al. 2021	Conteduca et al. 2022 A	Haas et al. 2021	Hirano et al. 2023	Hendriks et al. 2018	Gurioli et al. 2020	Conteduca et al. 2017 B	Salvi et al. 2016	Salvi et al. 2015	Lorente et al. 2018	Gu et al. 2023	Chung et al. 2019	De Laere et al. 2018	Annala et al. 2018	Moses et al. 2020	Mizuno et al. 2021	Antonarakis et al. 2017
Year	2020	2024	2023	2021	2022	2021	2023	2018	2020	2017	2016	2015	2018	2023	2019	2018	2018	2020	2021	2017
1. Marker, objectives, pre-specified hypotheses	1	1	1	1	1	1	1	1	1	1	1	1	1	1	1	1	1	1	1	1
2. Characteristics of patients	1	1	1	1	1	1	1	1	1	1	1	1	1	1	1	1	1	1	1	1
3. Treatments.	1	1	1	1	1	1	1	0	1	1	1	1	1	1	1	1	1	1	1	1
4. Biological material	1	1	1	1	1	1	1	1	1	1	1	1	1	1	1	1	1	1	1	1
5. Assay, protocol, blinding	1	1	1	1	1	1	1	1	1	1	1	1	1	1	1	1	1	0	1	1
6. Case selection, time period, end of follow-up, median follow-up	1	1	1	1	0	1	1	1	0	1	0	0	1	0	1	1	1	0	1	1
7. Define all clinical endpoints	1	1	1	1	1	1	1	0	1	1	1	0	1	1	1	1	1	1	1	1
8. List all candidate variables	0	1	1	1	0	1	0	0	0	0	1	0	1	0	0	1	1	0	1	1
9. Explain sample size / effect size	1	0	0	1	0	0	0	0	0	0	0	0	0	0	0	0	0	0	0	1
10. Specify all statistical methods, variable selection, assumptions, missing data	1	1	1	1	1	1	1	0	1	1	1	1	1	1	1	1	1	1	1	1
11. Clarify how marker values were handled, cutpoint determination	1	1	1	1	1	1	1	1	1	1	1	1	1	1	1	1	1	1	1	1
12. Flow of patients	1	1	1	1	0	0	1	0	0	0	0	0	1	0	0	0	1	0	1	0
13. Demographic characteristics prognostic variables, missing values	1	1	1	0	1	1	1	1	1	1	1	1	1	1	1	1	1	1	1	1

14. Relation of marker to standard prognostic variables	1	1	1	0	0	0	0	0	0	0	0	1	0	1	0	1	1	0	0	0
15. Univariable analyses	1	1	1	1	1	1	1	1	1	1	1	1	1	1	1	1	1	1	1	1
16. Key multivariable analyses: report estimated effect. For final model, all other variables in the model	0	1	1	1	1	1	0	0	0	1	1	1	1	1	0	1	1	0	1	1
17. Estimated effects with confidence intervals from an analysis with marker and standard prognostic variable	0	0	0	0	1	1	0	0	0	1	0	1	1	0	0	1	1	0	1	1
18. Further investigations (assumptions, sensitivity analyses, internal validation)	0	0	0	1	0	1	0	0	0	0	1	1	1	0	0	0	0	0	1	0
19. Interpret results in context of pre-specified hypotheses and other studies; include limitations	1	1	1	1	1	1	1	1	1	1	1	1	1	1	1	1	1	1	1	1
20. Implications for future research	1	1	1	1	1	1	1	1	1	1	1	1	1	1	1	1	1	1	1	1
Total	16	17	17	17	14	17	14	10	12	15	15	15	18	14	13	17	18	11	18	17
Follows REMARK checklist	Yes	Yes	Yes	Yes	No	Yes	No	No	No	Yes	Yes	Yes	Yes	No	No	Yes	Yes	No	Yes	Yes

Reference	(41)	(42)	(43)	(44)	(45)	(46)	(47)	(48)	(49)	(50)	(51)	(52)	(53)	(54)	(55)	(56)	(57)	(58)	(59)	(60)
Study	Graf et al. 2020	Wüstmann et al. 2023	Fettke et al. 2020	Conteduca et al. 2022 B	Schlack et al. 2022	De Laere et al. 2017	Sepe et al. 2019	Scher et al. 2021	Filon et al. 2023	Tolmeijer et al. 2023	De Laere et al. 2021	Zhu et al. 2021	Torquato et al. 2019	Du et al. 2020	Belic et al. 2018	Maillet et al. 2021 A	Zhu et al. 2022	Maillet et al. 2021 B	Fettke et al. 2021 A	Boerrigter et al. 2021
Year	2020	2023	2020	2022	2022	2017	2019	2021	2023	2023	2021	2021	2019	2020	2018	2021	2022	2021	2021	2021
1. Marker, objectives, pre-specified hypotheses	1	1	1	1	1	1	1	1	1	1	1	1	1	1	1	1	1	1	1	1
2. Characteristics of patients	1	1	1	1	1	1	1	1	0	1	1	1	1	1	1	1	1	1	1	1
3. Treatments.	1	1	0	1	1	1	1	1	1	1	1	1	1	1	1	0	1	0	0	1
4. Biological material	1	1	1	1	1	1	1	1	1	1	1	1	1	1	1	1	1	1	1	1
5. Assay, protocol, blinding	1	1	1	1	1	1	1	1	1	1	1	1	1	1	1	1	1	1	1	1
6. Case selection, time period, end of follow-up, median follow-up	1	1	1	1	1	1	1	1	0	1	0	1	0	1	0	0	0	1	1	1
7. Define all clinical endpoints	1	0	1	1	1	1	1	1	0	1	1	1	1	1	0	1	0	1	1	1
8. List all candidate variables	1	0	1	1	0	0	0	1	0	0	1	0	1	0	1	0	0	0	0	1
9. Explain sample size / effect size	0	0	0	0	0	0	0	0	0	0	0	0	0	0	0	0	0	0	0	0
10. Specify all statistical methods, variable selection, assumptions, missing data	1	1	1	1	1	1	1	1	1	1	1	1	1	1	1	1	1	1	1	1
11. Clarify how marker values were handled, cutpoint determination	1	1	1	1	1	1	1	1	1	1	1	1	1	1	1	1	1	1	1	1
12. Flow of patients	1	0	1	0	0	0	0	1	0	1	1	0	1	1	1	0	0	0	0	1
13. Demographic characteristics prognostic variables, missing values	1	0	1	1	1	1	1	1	0	1	1	1	1	1	1	1	1	1	1	1

14. Relation of marker to standard prognostic variables	1	0	0	1	1	0	0	1	0	0	0	1	0	0	1	0	0	1	0	0
15. Univariable analyses	1	1	1	1	1	1	1	1	1	1	1	1	1	1	1	1	1	1	1	1
16. Key multivariable analyses: report estimated effect. For final model, all other variables in the model	1	1	1	1	1	0	0	1	0	1	1	1	1	1	1	1	1	1	1	1
17. Estimated effects with confidence intervals from an analysis with marker and standard prognostic variable	1	1	0	1	0	0	0	0	0	0	0	0	1	0	1	0	0	0	0	0
18. Further investigations (assumptions, sensitivity analyses, internal validation)	0	0	1	0	0	1	0	1	0	0	0	0	0	0	0	0	0	0	0	0
19. Interpret results in context of pre-specified hypotheses and other studies; include limitations	1	1	1	1	1	1	1	1	1	1	1	1	1	1	1	1	1	1	1	1
20. Implications for future research	1	1	1	1	1	1	1	1	1	1	1	1	1	1	1	1	1	1	1	1
Total	18	13	16	17	15	14	13	18	9	15	15	15	16	15	16	12	12	14	13	16
Follows REMARK checklist	Yes	No	Yes	Yes	Yes	No	No	Yes	No	Yes	Yes	Yes	Yes	Yes	Yes	No	No	No	No	Yes

Reference	(61)	(62)	(63)	(64)	(65)	(66)	(67)	(68)	(69)	(70)	(71)	(72)	(73)	(74)	(75)	(76)	(77)	(78)	(79)	(80)
Study	Danila et al. 2011	Conteduca et al. 2019	Scher et al. 2017 A	Ma 2023	McKay et al. 2021	Scher et al. 2017 B	Jayaram et al. 2019	Romanel et al. 2015	Fettke et al. 2021 B	Kwan et al. 2021	Tao et al. 2023	Joncas et al. 2019	Detassis et al. 2024	Jayaram et al. 2021	Francolini et al. 2022	Buelens et al. 2018	Kohli et al. 2018 A	Sharova et al. 2021	Nørgaard et al. 2023	Benoist et al. 2020
Year	2011	2019	2017	2023	2021	2017	2019	2015	2021	2021	2023	2019	2024	2021	2022	2018	2018	2021	2023	2020
1. Marker, objectives, pre-specified hypotheses	1	1	1	1	1	1	1	1	1	1	1	1	1	1	1	1	1	1	1	1
2. Characteristics of patients	1	1	1	1	1	1	1	1	1	1	1	0	1	1	1	1	1	1	1	1
3. Treatments.	1	1	0	1	1	1	1	1	1	1	1	0	1	1	1	1	1	1	1	1
4. Biological material	1	1	1	1	1	1	1	1	1	1	1	1	1	1	1	1	1	1	1	1
5. Assay, protocol, blinding	1	1	1	1	1	1	1	1	1	1	1	1	1	1	1	1	1	1	1	1
6. Case selection, time period, end of follow-up, median follow-up	1	1	1	1	1	0	1	1	1	1	1	1	0	1	1	1	1	1	1	0
7. Define all clinical endpoints	1	1	1	1	1	1	0	1	1	1	1	0	1	1	0	1	1	1	1	1
8. List all candidate variables	0	0	1	0	1	1	1	1	0	0	1	0	0	1	0	0	1	1	1	0
9. Explain sample size / effect size	1	0	0	0	1	0	0	0	0	0	1	0	0	0	0	0	0	0	0	0
10. Specify all statistical methods, variable selection, assumptions, missing data	1	1	1	1	1	1	1	1	1	1	1	1	1	1	1	1	1	1	1	1
11. Clarify how marker values were handled, cutpoint determination	1	1	1	1	1	1	1	1	1	1	1	1	1	1	1	1	1	1	1	1
12. Flow of patients	0	0	1	0	1	1	1	1	0	0	1	0	0	0	0	1	1	0	1	1
13. Demographic characteristics prognostic variables, missing values	1	1	1	1	1	1	1	1	1	1	1	1	0	0	1	1	1	1	1	1

14. Relation of marker to standard prognostic variables	0	1	0	1	0	0	1	0	0	0	0	0	0	1	1	0	0	0	1	0
15. Univariable analyses	1	1	1	1	1	1	1	1	1	1	1	1	1	1	1	1	1	1	1	1
16. Key multivariable analyses: report estimated effect. For final model, all other variables in the model	0	1	1	1	0	1	1	1	1	1	1	0	0	1	0	1	1	1	1	1
17. Estimated effects with confidence intervals from an analysis with marker and standard prognostic variable	0	0	0	0	0	1	1	1	0	1	1	0	0	0	0	0	0	0	1	1
18. Further investigations (assumptions, sensitivity analyses, internal validation)	0	1	0	0	0	0	0	1	0	1	1	0	0	0	0	1	1	0	0	0
19. Interpret results in context of pre-specified hypotheses and other studies; include limitations	1	1	1	1	1	1	1	1	1	1	1	1	1	1	1	1	1	1	1	1
20. Implications for future research	1	1	1	1	1	1	1	1	1	1	1	1	1	1	1	1	1	1	1	1
Total	14	16	15	15	16	16	17	18	14	16	19	10	11	15	13	16	17	15	18	15
Follows REMARK checklist	No	Yes	Yes	Yes	Yes	Yes	Yes	Yes	No	Yes	Yes	No	No	Yes	No	Yes	Yes	Yes	Yes	Yes

Reference	(81)	(82)	(83)	(84)	(85)	(86)	(87)	(88)
Study	Pal et al. 2018	Lolli et al. 2019	Wang et al. 2022	Nakamura et al. 2024	Del Re et al. 2017	De Laere et al. 2019 A	Groen et al. 2023	Dong et al. 2021
Year	2018	2019	2022	2024	2017	2019	2023	2021
1. Marker, objectives, pre-specified hypotheses	1	1	1	1	1	1	1	1
2. Characteristics of patients	1	1	1	1	1	1	1	1
3. Treatments.	1	1	1	1	1	1	0	0
4. Biological material	1	1	1	1	1	1	1	1
5. Assay, protocol, blinding	1	1	1	1	1	1	1	1
6. Case selection, time period, end of follow-up, median follow-up	1	1	1	0	1	1	0	1
7. Define all clinical endpoints	0	0	1	0	1	1	0	1
8. List all candidate variables	0	0	1	1	0	1	0	0
9. Explain sample size / effect size	0	0	0	0	0	1	0	0
10. Specify all statistical methods, variable selection, assumptions, missing data	1	1	1	1	1	1	1	1
11. Clarify how marker values were handled, cutpoint determination	1	1	1	1	1	1	1	1
12. Flow of patients	1	0	0	1	0	1	0	1
13. Demographic characteristics prognostic variables, missing values	1	1	1	1	1	1	1	1

14. Relation of marker to standard prognostic variables	0	1	1	1	1	0	1	0
15. Univariable analyses	1	1	1	1	1	1	1	1
16. Key multivariable analyses: report estimated effect. For final model, all other variables in the model	1	1	1	1	1	1	1	1
17. Estimated effects with confidence intervals from an analysis with marker and standard prognostic variable	0	1	1	0	0	1	0	0
18. Further investigations (assumptions, sensitivity analyses, internal validation)	0	0	0	0	0	0	0	0
19. Interpret results in context of pre-specified hypotheses and other studies; include limitations	1	1	1	1	1	1	1	1
20. Implications for future research	1	1	1	1	1	1	1	1
Total	14	15	17	15	15	18	12	14
Follows REMARK checklist	No	Yes	Yes	Yes	Yes	Yes	No	No

Table S2. Quality assessment for abstracts of observational studies (STROBE checklist)

Title	(89)	(90)	(91)	(92)	(93)	(94)	(95)	(96)	(97)	(98)	(99)	(100)	(101)	(102)	(103)	(104)	(105)
Study	Halabi et al. 2024 B	Antonarakis et al. 2014 B	To et al. 2018	Zhang et al. 2022	Shevrin et al. 2021	Efstathiou et al. 2022	Fleisher et al. 2015	de Bono et al. 2021	Kohli et al. 2017	Morra et al. 2019	Silberstein et al. 2017	Schlack et al. 2020	Alahi et al. 2023	Zhu et al. 2020	Chung et al. 2017	De Laere et al. 2019 B	Steuber et al. 2012
Year	2024	2014	2018	2022	2021	2022	2015	2021	2017	2019	2017	2020	2023	2020	2017	2019	2012
Title	0	0	0	0	0	1	1	1	0	0	0	0	0	0	0	0	0
Authors	1	1	1	1	1	1	1	1	1	1	1	1	1	1	1	1	1
Study design	1	1	1	1	1	1	1	1	1	1	1	1	0	0	1	1	1
Objective	1	1	1	1	1	1	1	1	1	1	1	1	1	1	1	1	1
Setting	0	0	0	0	0	0	0	0	1	0	1	0	0	0	1	0	1
Participants	1	1	1	1	1	1	1	1	1	1	1	1	1	1	1	1	1
Variables	1	1	1	1	1	1	1	1	1	1	1	1	1	1	1	1	1
Statistical methods	1	1	0	0	0	1	0	1	1	0	1	0	1	0	1	1	1
Participants	0	0	0	0	0	1	0	1	1	0	0	0	1	0	0	0	0
Main results	1	1	1	1	0	0	1	1	0	1	1	1	0	1	1	1	0
Conclusions	1	1	1	1	1	1	1	1	1	1	1	1	1	1	1	1	1
Total	8	8	7	7	6	9	8	10	9	7	9	7	7	6	9	8	8
Follows STROBE checklist	Yes	Yes	No	No	No	Yes	Yes	Yes	Yes	No	Yes	No	No	No	Yes	Yes	Yes

Title	(106)	(107)	(108)	(109)	(110)	(111)	(112)	(113)	(114)	(115)	(116)	(117)	(118)	(119)	(120)	(121)
Study	Yip et al. 2024	Boerrigter et al. 2020	Azad et al. 2016	Chi et al. 2015	Kohli et al. 2018 B	Wise et al. 2017	Conteduca et al. 2022 C	Cho et al. 2024	Gill et al. 2017	Morgan et al. 2017	Shiang et al. 2022	Isebia et al. 2023	Maruzzo et al. 2018	Giridhar et al. 2018	Antonarakis et al. 2023	Giridhar et al. 2017
Year	2024	2020	2016	2015	2018	2017	2022	2024	2017	2017	2022	2023	2018	2018	2023	2017
Title	1	1	0	0	0	0	0	0	0	0	0	0	0	0	0	0
Authors	1	1	1	1	1	1	1	1	1	1	1	1	1	1	1	1
Study design	1	1	1	1	1	1	1	0	1	1	1	1	1	1	1	1
Objective	1	1	1	1	1	1	1	1	1	1	1	1	1	1	1	1
Setting	1	1	0	0	1	0	1	0	0	0	0	0	1	1	1	1
Participants	1	1	1	1	1	1	1	1	1	1	1	1	1	1	1	1
Variables	1	1	0	1	1	1	1	1	1	1	1	1	1	1	1	1
Statistical methods	0	1	0	1	1	1	1	1	1	1	1	1	1	1	1	1
Participants	0	0	0	0	1	0	0	0	0	0	1	0	0	0	1	0
Main results	1	0	0	0	1	1	1	1	0	1	0	1	0	0	0	0
Conclusions	1	1	1	1	1	1	1	1	1	1	1	1	1	1	1	1
Total	9	9	5	7	10	8	9	7	7	8	8	8	8	8	9	8
Follows STROBE checklist	Yes	Yes	No	No	Yes	Yes	Yes	No	No	Yes	Yes	Yes	Yes	Yes	Yes	Yes

Table S2. Quality assessment for abstracts of randomized clinical trials (CONSORT for abstracts checklist)

Title	Author	Year	Title	Author	Trial design	Participants	Interventions	Objective	Outcome	Randomisation	Blinding	Numbers randomised	Recruitment	Numbers analysed	Outcome	Harms	Conclusions	Trial registration	Funding	Total	Follows CONSORT checklist
(122)	Chi et al. 2017	2017	1	1	1	1	1	1	1	1	0	1	1	1	1	0	1	1	0	14	Yes
(123)	Khalaf et al. 2018	2018	1	1	1	1	1	1	1	1	0	1	0	0	1	0	1	1	0	12	No

Note: in Tables S1-S3, when multiple articles have the same first author and year of publication, they are disambiguated with a capital letter (in order of first appearance in this document).

References

1. Fernandez-Perez MP, Perez-Navarro E, Alonso-Gordoa T, Conteduca V, Font A, Vázquez-Estévez S, et al. A correlative biomarker study and integrative prognostic model in chemotherapy-naïve metastatic castration-resistant prostate cancer treated with enzalutamide. Prostate. 2023 Mar;83(4):376–84.

2. Markowski MC, Wang H, Sullivan R, Rifkind I, Sinibaldi V, Schweizer MT, et al. A Multicohort Open-label Phase II Trial of Bipolar Androgen Therapy in Men with Metastatic Castration-resistant Prostate Cancer (RESTORE): A Comparison of Post-abiraterone Versus Post-enzalutamide Cohorts. European Urology. 2021 May 1;79(5):692–9.

3. Peter MR, Bilenky ,Misha, Shi ,Yuliang, Pu ,Jiajie, Kamdar ,Shivani, Hansen ,Aaron R, et al. A novel methylated cell-free DNA marker panel to monitor treatment response in metastatic prostate cancer. Epigenomics. 2022 Jul 1;14(13):811–22.

4. Sepe P, Procopio G, Pircher CC, Basso U, Caffo O, Cappelletti V, et al. A phase II study evaluating the efficacy of enzalutamide and the role of liquid biopsy for evaluation of ARv7 in mCRPC patients with measurable metastases including visceral disease (Excalibur study). Ther Adv Med Oncol. 2024 Feb 1;16:17588359231217958.

5. Del Re M, Conteduca V, Crucitta S, Gurioli G, Casadei C, Restante G, et al. Androgen receptor gain in circulating free DNA and splicing variant 7 in exosomes predict clinical outcome in CRPC patients treated with abiraterone and enzalutamide. Prostate Cancer Prostatic Dis. 2021 Jun;24(2):524–31.

6. Del Re M, Crucitta S, Sbrana A, Rofi E, Paolieri F, Gianfilippo G, et al. Androgen receptor (AR) splice variant 7 and full-length AR expression is associated with clinical outcome: a translational study in patients with castrate-resistant prostate cancer. BJU International. 2019;124(4):693–700.

7. Conteduca V, Wetterskog D, Sharabiani MTA, Grande E, Fernandez-Perez MP, Jayaram A, et al. Androgen receptor gene status in plasma DNA associates with worse outcome on enzalutamide or abiraterone for castration-resistant prostate cancer: a multi-institution correlative biomarker study. Annals of Oncology. 2017 Jul 1;28(7):1508–16.

8. Miyamoto DT, Lee RJ, Stott SL, Ting DT, Wittner BS, Ulman M, et al. Androgen Receptor Signaling in Circulating Tumor Cells as a Marker of Hormonally Responsive Prostate Cancer. Cancer Discovery. 2012 Nov 11;2(11):995–1003.

9. Antonarakis ES, Lu C, Wang H, Luber B, Nakazawa M, Roeser JC, et al. AR-V7 and resistance to enzalutamide and abiraterone in prostate cancer. N Engl J Med. 2014 Sep 11;371(11):1028–38.

10. Okegawa T, Ninomiya N, Masuda K, Nakamura Y, Tambo M, Nutahara K. AR-V7 in circulating tumor cells cluster as a predictive biomarker of abiraterone acetate and enzalutamide treatment in castration-resistant prostate cancer patients. The Prostate. 2018;78(8):576–82.

11. Seitz AK, Thoene S, Bietenbeck A, Nawroth R, Tauber R, Thalgott M, et al. AR-V7 in Peripheral Whole Blood of Patients with Castration-resistant Prostate Cancer: Association with Treatment-specific Outcome Under Abiraterone and Enzalutamide. European Urology. 2017 Nov 1;72(5):828–34.

12. Erb HHH, Sparwasser P, Diehl T, Hemmerlein-Thomas M, Tsaur I, Jüngel E, et al. AR-V7 Protein Expression in Circulating Tumour Cells Is Not Predictive of Treatment Response in mCRPC. Urologia Internationalis. 2020 Jan 17;104(3–4):253–62.

13. Todenhöfer T, Azad A, Stewart C, Gao J, Eigl BJ, Gleave ME, et al. AR-V7 Transcripts in Whole Blood RNA of Patients with Metastatic Castration Resistant Prostate Cancer Correlate with Response to Abiraterone Acetate. The Journal of Urology [Internet]. 2017 Jan [cited 2025 Mar 6]; Available from: https://www.auajournals.org/doi/10.1016/j.juro.2016.06.094

14. Scher HI, Graf RP, Schreiber NA, Jayaram A, Winquist E, McLaughlin B, et al. Assessment of the Validity of Nuclear-Localized Androgen Receptor Splice Variant 7 in Circulating Tumor Cells as a Predictive Biomarker for Castration-Resistant Prostate Cancer. JAMA Oncology. 2018 Sep 1;4(9):1179–86.

15. Lorenzo GD, Zappavigna S, Crocetto F, Giuliano M, Ribera D, Morra R, et al. Assessment of Total, PTEN–, and AR-V7+ Circulating Tumor Cell Count by Flow Cytometry in Patients with Metastatic Castration-Resistant Prostate Cancer Receiving Enzalutamide. Clinical Genitourinary Cancer. 2021 Oct 1;19(5):e286–98.

16. Zedan AH, Osther PJS, Assenholt J, Madsen JS, Hansen TF. Circulating miR-141 and miR-375 are associated with treatment outcome in metastatic castration resistant prostate cancer. Sci Rep. 2020 Jan 14;10(1):227.

17. Qu F, Xie W, Nakabayashi M, Zhang H, Jeong SH, Wang X, et al. Association of AR-V7 and Prostate-Specific Antigen RNA Levels in Blood with Efficacy of Abiraterone Acetate and Enzalutamide Treatment in Men with Prostate Cancer. Clinical Cancer Research. 2017 Jan 31;23(3):726–34.

18. Scher HI, Lu D, Schreiber NA, Louw J, Graf RP, Vargas HA, et al. Association of AR-V7 on Circulating Tumor Cells as a Treatment-Specific Biomarker With Outcomes and Survival in Castration-Resistant Prostate Cancer. JAMA Oncology. 2016 Nov 1;2(11):1441–9.

19. Brown LC, Halabi S, Schonhoft JD, Yang Q, Luo J, Nanus DM, et al. Circulating Tumor Cell Chromosomal Instability and Neuroendocrine Phenotype by Immunomorphology and Poor Outcomes in Men with mCRPC Treated with Abiraterone or Enzalutamide. Clinical Cancer Research. 2021 Jul 15;27(14):4077–88.

20. Gupta S, Hovelson DH, Kemeny G, Halabi S, Foo WC, Anand M, et al. Discordant and heterogeneous clinically relevant genomic alterations in circulating tumor cells vs plasma DNA from men with metastatic castration resistant prostate cancer. Genes, Chromosomes and Cancer. 2020;59(4):225–39.

21. Armstrong AJ, Luo J, Nanus DM, Giannakakou P, Szmulewitz RZ, Danila DC, et al. Prospective Multicenter Study of Circulating Tumor Cell AR-V7 and Taxane Versus Hormonal Treatment Outcomes in Metastatic Castration-Resistant Prostate Cancer. JCO Precis Oncol. 2020 Oct;(4):1285–301.

22. Halabi S, Guo S, Park JJ, Nanus DM, George DJ, Antonarakis ES, et al. The Impact of Circulating Tumor Cell HOXB13 RNA Detection in Men with Metastatic Castration-Resistant Prostate Cancer (mCRPC) Treated with Abiraterone or Enzalutamide. Clinical Cancer Research. 2024 Mar 15;30(6):1152–9.

23. Gupta S, Halabi S, Yang Q, Roy A, Tubbs A, Gore Y, et al. PSMA-positive Circulating Tumor Cell Detection and Outcomes with Abiraterone or Enzalutamide Treatment in Men with Metastatic Castrate-resistant Prostate Cancer. Clinical Cancer Research. 2023 May 15;29(10):1929–37.

24. Oeyen S, Liégeois V, De Laere B, Buys A, Strijbos M, Dirix P, et al. Automated enumeration and phenotypic characterization of CTCs and tdEVs in patients with metastatic castration resistant prostate cancer. Prostate Cancer Prostatic Dis. 2021 Jun;24(2):499–506.

25. Conteduca V, Casadei C, Scarpi E, Brighi N, Schepisi G, Lolli C, et al. Baseline Plasma Tumor DNA (ctDNA) Correlates with PSA Kinetics in Metastatic Castration-Resistant Prostate Cancer (mCRPC) Treated with Abiraterone or Enzalutamide. Cancers. 2022 Jan;14(9):2219.

26. Haas NB, LaRiviere MJ, Buckingham TH, Cherkas Y, Calara-Nielsen K, Foulk B, et al. Blood-based gene expression signature associated with metastatic castrate-resistant prostate cancer patient response to abiraterone plus prednisone or enzalutamide. Prostate Cancer Prostatic Dis. 2021 Jun;24(2):448–56.

27. Hirano H, Nagata M, Nagaya N, Nakamura S, Ashizawa T, Lu Y, et al. Bone scan index (BSI) scoring by using bone scintigraphy and circulating tumor cells (CTCs): predictive factors for enzalutamide effectiveness in patients with castration-resistant prostate cancer and bone metastases. Sci Rep. 2023 May 29;13(1):8704.

28. Hendriks RJ, Dijkstra S, Smit FP, Vandersmissen J, Van de Voorde H, Mulders PFA, et al. Epigenetic markers in circulating cell-free DNA as prognostic markers for survival of castration-resistant prostate cancer patients. The Prostate. 2018;78(5):336–42.

29. Gurioli G, Conteduca V, Lolli C, Schepisi G, Gargiulo S, Altavilla A, et al. Plasma AR Copy Number Changes and Outcome to Abiraterone and Enzalutamide. Front Oncol [Internet]. 2020 Sep 24 [cited 2025 Mar 7];10. Available from: https://www.frontiersin.org/journals/oncology/articles/10.3389/fonc.2020.567809/full

30. Conteduca V, Scarpi E, Caroli P, Salvi S, Lolli C, Burgio SL, et al. Circulating androgen receptor combined with 18F-fluorocholine PET/CT metabolic activity and outcome to androgen receptor signalling-directed therapies in castration-resistant prostate cancer. Sci Rep. 2017 Nov 14;7(1):15541.

31. Salvi S, Casadio V, Conteduca V, Lolli C, Gurioli G, Martignano F, et al. Circulating AR copy number and outcome to enzalutamide in docetaxel-treated metastatic castration-resistant prostate cancer. Oncotarget. 2016 May 13;7(25):37839–45.

32. Salvi S, Casadio V, Conteduca V, Burgio SL, Menna C, Bianchi E, et al. Circulating cell-free AR and CYP17A1 copy number variations may associate with outcome of metastatic castration-resistant prostate cancer patients treated with abiraterone. Br J Cancer. 2015 May;112(10):1717–24.

33. Lorente D, Olmos D, Mateo J, Dolling D, Bianchini D, Seed G, et al. Circulating tumour cell increase as a biomarker of disease progression in metastatic castration-resistant prostate cancer patients with low baseline CTC counts. Annals of Oncology. 2018 Jul 1;29(7):1554–60.

34. Gu T, Li J, Chen T, Zhu Q, Ding J. Circulating tumor cell quantification during abiraterone plus prednisone therapy may estimate survival in metastatic castration-resistant prostate cancer patients. Int Urol Nephrol. 2023 Apr 1;55(4):883–92.

35. Chung JS, Wang Y, Henderson J, Singhal U, Qiao Y, Zaslavsky AB, et al. Circulating Tumor Cell–Based Molecular Classifier for Predicting Resistance to Abiraterone and Enzalutamide in Metastatic Castration-Resistant Prostate Cancer. Neoplasia. 2019 Aug 1;21(8):802–9.

36. De Laere B, Oeyen S, Van Oyen P, Ghysel C, Ampe J, Ost P, et al. Circulating tumor cells and survival in abiraterone- and enzalutamide-treated patients with castration-resistant prostate cancer. The Prostate. 2018;78(6):435–45.

37. Annala M, Vandekerkhove G, Khalaf D, Taavitsainen S, Beja K, Warner EW, et al. Circulating Tumor DNA Genomics Correlate with Resistance to Abiraterone and Enzalutamide in Prostate Cancer. Cancer Discovery. 2018 Apr 1;8(4):444–57.

38. Moses M, Niu A, Lilly MB, Hahn AW, Nussenzveig R, Ledet E, et al. Circulating-tumor DNA as predictor of enzalutamide response post-abiraterone treatment in metastatic castration-resistant prostate cancer. Cancer Treatment and Research Communications. 2020 Jan 1;24:100193.

39. Mizuno K, Sumiyoshi T, Okegawa T, Terada N, Ishitoya S, Miyazaki Y, et al. Clinical Impact of Detecting Low-Frequency Variants in Cell-Free DNA on Treatment of Castration-Resistant Prostate Cancer. Clinical Cancer Research. 2021 Nov 15;27(22):6164–73.

40. Antonarakis ES, Lu C, Luber B, Wang H, Chen Y, Zhu Y, et al. Clinical Significance of Androgen Receptor Splice Variant-7 mRNA Detection in Circulating Tumor Cells of Men With Metastatic Castration-Resistant Prostate Cancer Treated With First- and Second-Line Abiraterone and Enzalutamide. JCO. 2017 Jul;35(19):2149–56.

41. Graf RP, Hullings M, Barnett ES, Carbone E, Dittamore R, Scher HI. Clinical Utility of the Nuclear-localized AR-V7 Biomarker in Circulating Tumor Cells in Improving Physician Treatment Choice in Castration-resistant Prostate Cancer. European Urology. 2020 Feb 1;77(2):170–7.

42. Wüstmann N, Seitzer K, Humberg V, Vieler J, Grundmann N, Steinestel J, et al. Co-expression and clinical utility of AR-FL and AR splice variants AR-V3, AR-V7 and AR-V9 in prostate cancer. Biomarker Research. 2023 Apr 5;11(1):37.

43. Fettke H, Kwan EM, Docanto MM, Bukczynska P, Ng N, Graham LJK, et al. Combined Cell-free DNA and RNA Profiling of the Androgen Receptor: Clinical Utility of a Novel Multianalyte Liquid Biopsy Assay for Metastatic Prostate Cancer. European Urology. 2020 Aug 1;78(2):173–80.

44. Conteduca V, Scarpi E, Caroli P, Lolli C, Gurioli G, Brighi N, et al. Combining liquid biopsy and functional imaging analysis in metastatic castration-resistant prostate cancer helps predict treatment outcome. Molecular Oncology. 2022;16(2):538–48.

45. Schlack K, Seitzer K, Wüstmann N, Humberg V, Grundmann N, Steinestel J, et al. Comparison of circulating tumor cells and AR-V7 as clinical biomarker in metastatic castration-resistant prostate cancer patients. Sci Rep. 2022 Jul 13;12(1):11846.

46. De Laere B, van Dam PJ, Whitington T, Mayrhofer M, Diaz EH, Van den Eynden G, et al. Comprehensive Profiling of the Androgen Receptor in Liquid Biopsies from Castration-resistant Prostate Cancer Reveals Novel Intra-AR Structural Variation and Splice Variant Expression Patterns. European Urology. 2017 Aug 1;72(2):192–200.

47. Sepe P, Verzoni E, Miodini P, Claps M, Ratta R, Martinetti A, et al. Could Circulating Tumor Cells and ARV7 Detection Improve Clinical Decisions in Metastatic Castration-Resistant Prostate Cancer? The Istituto Nazionale dei Tumori (INT) Experience. Cancers. 2019 Jul;11(7):980.

48. Scher HI, Armstrong AJ, Schonhoft JD, Gill A, Zhao JL, Barnett E, et al. Development and validation of circulating tumour cell enumeration (Epic Sciences) as a prognostic biomarker in men with metastatic castration-resistant prostate cancer. European Journal of Cancer. 2021 Jun 1;150:83–94.

49. Filon M, Yang B, Purohit TA, Schehr J, Singh A, Bigarella M, et al. Development of a multiplex assay to assess activated p300/CBP in circulating prostate tumor cells. Oncotarget. 2023 Jan 12;14:738–46.

50. Tolmeijer SH, Boerrigter E, Sumiyoshi T, Kwan EM, Ng SWS, Annala M, et al. Early On-treatment Changes in Circulating Tumor DNA Fraction and Response to Enzalutamide or Abiraterone in Metastatic Castration-Resistant Prostate Cancer. Clinical Cancer Research. 2023 Aug 1;29(15):2835–44.

51. De Laere B, Crippa A, Mortezavi A, Ghysel C, Rajan P, Eklund M, et al. Increased Pathway Complexity Is a Prognostic Biomarker in Metastatic Castration-Resistant Prostate Cancer. Cancers. 2021 Jan;13(7):1588.

52. Zhu S, Ni Y, Sun G, Wang Z, Chen J, Zhang X, et al. Exosomal TUBB3 mRNA expression of metastatic castration-resistant prostate cancer patients: Association with patient outcome under abiraterone. Cancer Medicine. 2021;10(18):6282–90.

53. Torquato S, Pallavajjala A, Goldstein A, Valda Toro P, Silberstein JL, Lee J, et al. Genetic Alterations Detected in Cell-Free DNA Are Associated With Enzalutamide and Abiraterone Resistance in Castration-Resistant Prostate Cancer. JCO Precis Oncol. 2019 Apr;(3):1–14.

54. Du M, Tian Y, Tan W, Wang L, Wang L, Kilari D, et al. Plasma cell-free DNA-based predictors of response to abiraterone acetate/prednisone and prognostic factors in metastatic castration-resistant prostate cancer. Prostate Cancer Prostatic Dis. 2020 Dec;23(4):705–13.

55. Belic J, Graf R, Bauernhofer T, Cherkas Y, Ulz P, Waldispuehl-Geigl J, et al. Genomic alterations in plasma DNA from patients with metastasized prostate cancer receiving abiraterone or enzalutamide. International Journal of Cancer. 2018;143(5):1236–48.

56. Maillet D, Allioli N, Péron J, Plesa A, Decaussin-Petrucci M, Tartas S, et al. Her2 Expression in Circulating Tumor Cells Is Associated with Poor Outcomes in Patients with Metastatic Castration-Resistant Prostate Cancer. Cancers. 2021 Jan;13(23):6014.

57. Zhu S, Ni Y, Wang Z, Zhang X, Zhang Y, Zhao F, et al. Plasma Exosomal AKR1C3 mRNA Expression Is a Predictive and Prognostic Biomarker in Patients with Metastatic Castration-Resistant Prostate Cancer. The Oncologist. 2022 Nov 1;27(11):e870–7.

58. Maillet D, Allioli N, Peron J, Plesa A, Decaussin-Petrucci M, Tartas S, et al. Improved Androgen Receptor Splice Variant 7 Detection Using a Highly Sensitive Assay to Predict Resistance to Abiraterone or Enzalutamide in Metastatic Prostate Cancer Patients. European Urology Oncology. 2021 Aug 1;4(4):609–17.

59. Fettke H, Kwan EM, Bukczynska P, Steen JA, Docanto M, Ng N, et al. Independent prognostic impact of plasma NCOA2 alterations in metastatic castration-resistant prostate cancer. The Prostate. 2021;81(13):992–1001.

60. Boerrigter E, Benoist GE, van Oort IM, Verhaegh GW, van Hooij O, Groen L, et al. Liquid biopsy reveals KLK3 mRNA as a prognostic marker for progression free survival in patients with metastatic castration-resistant prostate cancer undergoing first-line abiraterone acetate and prednisone treatment. Molecular Oncology. 2021;15(9):2453–65.

61. Danila DC, Anand A, Sung CC, Heller G, Leversha MA, Cao L, et al. TMPRSS2-ERG Status in Circulating Tumor Cells as a Predictive Biomarker of Sensitivity in Castration-Resistant Prostate Cancer Patients Treated With Abiraterone Acetate. European Urology. 2011 Nov 1;60(5):897–904.

62. Conteduca V, Scarpi E, Matteucci F, Caroli P, Ravaglia G, Fantini L, et al. Multimodal Approach to Outcome Prediction in Metastatic Castration-Resistant Prostate Cancer by Integrating Functional Imaging and Plasma DNA Analysis. JCO Precis Oncol. 2019 Apr;(3):1–13.

63. Scher HI, Graf RP, Schreiber NA, McLaughlin B, Lu D, Louw J, et al. Nuclear-specific AR-V7 Protein Localization is Necessary to Guide Treatment Selection in Metastatic Castration-resistant Prostate Cancer. European Urology. 2017 Jun 1;71(6):874–82.

64. Ma Y. OCT4-positive circulating tumor cells may predict a poor prognosis in patients with metastatic castration-resistant prostate cancer treated with abiraterone plus prednisone therapy. Oncology Letters. 2023 Oct 1;26(4):1–9.

65. McKay RR, Kwak L, Crowdis JP, Sperger JM, Zhao SG, Xie W, et al. Phase II Multicenter Study of Enzalutamide in Metastatic Castration-Resistant Prostate Cancer to Identify Mechanisms Driving Resistance. Clinical Cancer Research. 2021 Jul 1;27(13):3610–9.

66. Scher HI, Graf RP, Schreiber NA, McLaughlin B, Jendrisak A, Wang Y, et al. Phenotypic Heterogeneity of Circulating Tumor Cells Informs Clinical Decisions between AR Signaling Inhibitors and Taxanes in Metastatic Prostate Cancer. Cancer Research. 2017 Oct 15;77(20):5687–98.

67. Jayaram A, Wingate A, Wetterskog D, Conteduca V, Khalaf D, Sharabiani MTA, et al. Plasma Androgen Receptor Copy Number Status at Emergence of Metastatic Castration-Resistant Prostate Cancer: A Pooled Multicohort Analysis. JCO Precis Oncol. 2019 Sep;(3):1–13.

68. Romanel A, Tandefelt DG, Conteduca V, Jayaram A, Casiraghi N, Wetterskog D, et al. Plasma AR and abiraterone-resistant prostate cancer. Science Translational Medicine. 2015 Nov 4;7(312):312re10-312re10.

69. Fettke H, Kwan EM, Bukczynska P, Ng N, Nguyen-Dumont T, Southey MC, et al. Prognostic Impact of Total Plasma Cell-free DNA Concentration in Androgen Receptor Pathway Inhibitor–treated Metastatic Castration-resistant Prostate Cancer. European Urology Focus. 2021 Nov 1;7(6):1287–91.

70. Kwan EM, Dai C, Fettke H, Hauser C, Docanto MM, Bukczynska P, et al. Plasma Cell–Free DNA Profiling of PTEN-PI3K-AKT Pathway Aberrations in Metastatic Castration-Resistant Prostate Cancer. JCO Precis Oncol. 2021 Apr;(5):622–37.

71. Tao W, Luo ZH, He YD, Wang BY, Xia TL, Deng WM, et al. Plasma extracellular vesicle circRNA signature and resistance to abiraterone in metastatic castration-resistant prostate cancer. Br J Cancer. 2023 Mar;128(7):1320–32.

72. Joncas FH, Lucien F, Rouleau M, Morin F, Leong HS, Pouliot F, et al. Plasma extracellular vesicles as phenotypic biomarkers in prostate cancer patients. The Prostate. 2019;79(15):1767–76.

73. Detassis S, Precazzini F, Grasso M, Del Vescovo V, Maines F, Caffo O, et al. Plasma microRNA Signature as Companion Diagnostic for Abiraterone Acetate Treatment in Metastatic Castration-Resistant Prostate Cancer: A Pilot Study. International Journal of Molecular Sciences. 2024 Jan;25(11):5573.

74. Jayaram A, Wingate A, Wetterskog D, Wheeler G, Sternberg CN, Jones R, et al. Plasma tumor gene conversions after one cycle abiraterone acetate for metastatic castration-resistant prostate cancer: a biomarker analysis of a multicenter international trial. Annals of Oncology. 2021 Jun 1;32(6):726–35.

75. Francolini G, Loi M, Ciccone LP, Detti B, Di Cataldo V, Pinzani P, et al. Prospective assessment of AR splice variant and multi-biomarker expression on circulating tumor cells of mCRPC patients undergoing androgen receptor targeted agents: interim analysis of PRIMERA trial (NCT04188275). Med Oncol. 2022 Jun 10;39(8):119.

76. Buelens S, Claeys T, Dhondt B, Poelaert F, Vynck M, Yigit N, et al. Prognostic and Therapeutic Implications of Circulating Androgen Receptor Gene Copy Number in Prostate Cancer Patients Using Droplet Digital Polymerase Chain Reaction. Clinical Genitourinary Cancer. 2018 Jun 1;16(3):197-205.e5.

77. Kohli M, Li J, Du M, Hillman DW, Dehm SM, Tan W, et al. Prognostic association of plasma cell-free DNA-based androgen receptor amplification and circulating tumor cells in pre-chemotherapy metastatic castration-resistant prostate cancer patients. Prostate Cancer Prostatic Dis. 2018 Sep;21(3):411–8.

78. Sharova E, Maruzzo M, Del Bianco P, Cavallari I, Pierantoni F, Basso U, et al. Prognostic Stratification of Metastatic Prostate Cancer Patients Treated With Abiraterone and Enzalutamide Through an Integrated Analysis of Circulating Free microRNAs and Clinical Parameters. Front Oncol [Internet]. 2021 Mar 16 [cited 2025 Mar 20];11. Available from: https://www.frontiersin.org/journals/oncology/articles/10.3389/fonc.2021.626104/full

79. Nørgaard M, Bjerre MT, Fredsøe J, Vang S, Jensen JB, De Laere B, et al. Prognostic Value of Low-Pass Whole Genome Sequencing of Circulating Tumor DNA in Metastatic Castration-Resistant Prostate Cancer. Clinical Chemistry. 2023 Apr 3;69(4):386–98.

80. Benoist GE, van Oort IM, Boerrigter E, Verhaegh GW, van Hooij O, Groen L, et al. Prognostic Value of Novel Liquid Biomarkers in Patients with Metastatic Castration-Resistant Prostate Cancer Treated with Enzalutamide: A Prospective Observational Study. Clinical Chemistry. 2020 Jun 1;66(6):842–51.

81. Pal SK, He M, Chen L, Yang L, Pillai R, Twardowski P, et al. Synaptophysin expression on circulating tumor cells in patients with castration resistant prostate cancer undergoing treatment with abiraterone acetate or enzalutamide. Urologic Oncology: Seminars and Original Investigations. 2018 Apr 1;36(4):162.e1-162.e6.

82. Lolli C, De Lisi D, Conteduca V, Gurioli G, Scarpi E, Schepisi G, et al. Testosterone levels and androgen receptor copy number variations in castration-resistant prostate cancer treated with abiraterone or enzalutamide. The Prostate. 2019;79(11):1211–20.

83. Wang S, Du P, Cao Y, Tang X, Yang X, Ma J, et al. The association of AR-V7 with resistance to Abiraterone in metastatic castration-resistant prostate cancer. Journal of Men's Health. 2022;18(3):1–8.

84. Nakamura S, Nagata M, Nagaya N, Ashizawa T, Hirano H, Lu Y, et al. The Detection and Negative Reversion of Circulating Tumor Cells as Prognostic Biomarkers for Metastatic Castration-Resistant Prostate Cancer with Bone Metastases Treated by Enzalutamide. Cancers. 2024 Jan;16(4):772.

85. Del Re M, Biasco E, Crucitta S, Derosa L, Rofi E, Orlandini C, et al. The Detection of Androgen Receptor Splice Variant 7 in Plasma-derived Exosomal RNA Strongly Predicts Resistance to Hormonal Therapy in Metastatic Prostate Cancer Patients. European Urology. 2017 Apr 1;71(4):680–7.

86. De Laere B, Oeyen S, Mayrhofer M, Whitington T, van Dam PJ, Van Oyen P, et al. TP53 Outperforms Other Androgen Receptor Biomarkers to Predict Abiraterone or Enzalutamide Outcome in Metastatic Castration-Resistant Prostate Cancer. Clinical Cancer Research. 2019 Mar 15;25(6):1766–73.

87. Groen L, Kloots I, Englert D, Seto K, Estafanos L, Smith P, et al. Transcriptome Profiling of Circulating Tumor Cells to Predict Clinical Outcomes in Metastatic Castration-Resistant Prostate Cancer. International Journal of Molecular Sciences. 2023 Jan;24(10):9002.

88. Dong B, Fan L, Yang B, Chen W, Li Y, Wu K, et al. Use of Circulating Tumor DNA for the Clinical Management of Metastatic Castration-Resistant Prostate Cancer: A Multicenter, Real-World Study. Journal of the National Comprehensive Cancer Network. 2021 May 14;19(8):905–14.

89. Halabi S, Luo B, Guo SS, Knutson T, Lyman J, Kobilka A, et al. A clinical-genetic (CG) circulating tumor DNA (ctDNA)-based prognostic model for predicting overall survival (OS) in men with metastatic castrate-resistant prostate cancer (mCRPC) treated with potent androgen receptor inhibition (Alliance). JCO. 2024 Jun;42(16_suppl):5007–5007.

90. Antonarakis ES, Lu C, Wang H, Luber B, Nakazawa M, Roeser JC, et al. Abstract 2910: Androgen receptor splice variant-7 predicts resistance to enzalutamide in patients with castration-resistant prostate cancer. Cancer Research. 2014 Oct 1;74(19_Supplement):2910.

91. To SQ, Kwan E, Fettke H, Mant A, Docanto M, Martelotto L, et al. Abstract 2593: AR-V7 and AR-V9 expression is not predictive of response to AR-axis targeting agents in metastatic castration-resistant prostate cancer. Cancer Research. 2018 Jul 1;78(13_Supplement):2593.

92. Zhang J, Zimmermann B, Galletti G, Halabi S, Gjyrezi A, Yang Q, et al. Association of circulating tumor cell RB1 loss RNA signature with outcomes and immune phenotypes in men with mCRPC. JCO. 2022 Feb 20;40(6_suppl):139–139.

93. Shevrin DH, Yang M, Imas P, Gulukota K, Northshore University Healthsystem. Associations of circulating cell-free DNA (cfDNA) and clinical outcomes in metastatic castrate-resistant prostate cancer (mCRPC). JCO. 2021 Feb 20;39(6_suppl):137–137.

94. Efstathiou E, Attard G, Lucas J, Thomas S, Gormley M, Aguilar-Bonavides C, et al. Blood biomarkers and association with clinical outcomes in metastatic castration-resistant prostate cancer (mCRPC): prespecified

longitudinal analysis from the ACIS study of apalutamide (APA) or placebo combined with abiraterone acetate plus prednisone (AAP). JCO. 2022 Feb 20;40(6_suppl):142–142.

95. Fleisher M, Danila DC, Fizazi K, Hirmand M, Selby B, Phung D, et al. Circulating tumor cell (CTC) enumeration in men with metastatic castration-resistant prostate cancer (mCRPC) treated with enzalutamide post-chemotherapy (phase 3 AFFIRM study). JCO. 2015 May 20;33(15_suppl):5035–5035.

96. Bono JS de, Pantel K, Efstathiou E, Sternberg CN, Gauna DC, Fizazi K, et al. 614P Circulating tumor cell (CTC) morphologic sub-types present prior to treatment in the CARD trial identify therapy resistance. Annals of Oncology. 2021 Sep 1;32:S653–4.

97. Kohli M, Li J, Du M, Hillman DW, Tan W, Carlson R, et al. Circulating tumor cells (CTCs) and plasma cell free DNA (cfDNA) androgen receptor amplification (ARamp)-based prognosis in metastatic castration-resistant prostate cancer (mCRPC). JCO. 2017 Feb 20;35(6_suppl):152–152.

98. Morra R, Zappavigna S, Facchini G, Ribera D, Morelli F, Luce A, et al. Circulating tumor cells count in prostate cancer patients treated with enzalutamide: The LANZA study. Tumori. 2019 Nov 1;105(6_suppl):1–216.

99. Silberstein J, Luber B, Wang H, Lu C, Chen Y, Zhu Y, et al. Clinical significance of AR mRNA quantification from circulating tumor cells (CTCs) in men with metastatic castration-resistant prostate cancer (mCRPC) treated with abiraterone (Abi) or enzalutamide (Enza). JCO. 2017 Feb 20;35(6_suppl):132–132.

100. Schlack K, Seitzer K, Boegemann M, Krabbe LM, Schrader AJ, Grundmann N, et al. Combinatorial expression of androgen receptor splice variants: No predictive value in castration-resistant prostate cancer patients treated with enzalutamide (enza) or abiraterone (abi). JCO. 2020 May 20;38(15_suppl):e17547–e17547.

101. Alahi I, Chauhan PS, Shiang AL, Webster J, Dang HX, Greiner L, et al. Abstract 6698: Combinatorial genomic and epigenomic cell-free DNA analysis of high-risk metastatic castration resistant prostate cancer reveals prognostic liquid biopsy signatures. Cancer Research. 2023 Apr 4;83(7_Supplement):6698.

102. Zhu S, Sun G, Zhao X, Zhao J, Chen J, Shen P, et al. 650P Comparing the predictive value of exosome, circulating tumor cells, and tumor tissue in detecting AR-V7 among metastatic castration-resistant prostate cancer treated with abiraterone. Annals of Oncology. 2020 Sep 1;31:S530.

103. Chung JS, Wang Y, James H, Singhal U, Qiao Y, Zaslavsky A, et al. PD71-06 CTC-BASED GENE EXPRESSION FOR PREDICTING RESISTANCE TO ABIRATERONE AND ENZALUTAMIDE IN MCRPC. The Journal of Urology [Internet]. 2017 Apr [cited 2025 Mar 9]; Available from: https://www.auajournals.org/doi/10.1016/j.juro.2017.02.3173

104. Laere BD, Crippa A, Ghysel C, Ost P, Rajan P, Eklund M, et al. Elevated driver mutational burden or number of perturbed pathways and poor response to abiraterone or enzalutamide in metastatic castration-resistant prostate cancer. Annals of Oncology. 2019 Oct 1;30:v30–1.

105. Steuber T, Stroelin P, Schlomm T, Heinzer H, Becker A, Budäus L, et al. 954 Evaluation of circulating tumor cells to predict metastatic progression in men treated with abirateron acetat for castration resistant prostate cancer: a sub-analysis of the german named patient program. The Journal of Urology [Internet]. 2012 Apr [cited 2025 Mar 5]; Available from: https://www.auajournals.org/doi/10.1016/j.juro.2012.02.1052

106. Yip S, Fizazi K, Laird D, Matsubara N, Azad A, Joung JY, et al. Exploration of circulating tumor cell (CTC) conversion and CTC0 as prognostic biomarkers for efficacy in TALAPRO-2: Phase 3 study of talazoparib (TALA) + enzalutamide (ENZA) vs placebo (PBO) + ENZA as first-line (1L) treatment in patients (pts) with metastatic castration-resistant prostate cancer (mCRPC). JCO. 2024 Jun;42(16_suppl):5023–5023.

107. Boerrigter E, Benoist GE, van Oort IM, Verhaegh GW, van Hooij O, Groen L, et al. Abstract 1413: Exploring the prognostic value of microRNAs and drug exposure in patients with metastatic castration resistant prostate cancer treated with abiraterone: a prospective observational study. Cancer Research. 2020 Aug 15;80(16_Supplement):1413.

108. Azad A, Wyatt A, Volik, S, Gleave M, Collins `Colin, Chi K. Genomic alterations in cell-free dna and enzalutamide resistance in castration-resistant prostate cancer. Asia-Pacific Journal of Clinical Oncology. 2016;12(S3):44–51.

109. Chi K, Azad A, Volik S, Haegert A, Zalcberg J, Bihan SL, et al. 2504 Genomic predictive and prognostic factors from plasma cell-free DNA (cfDNA) for metastatic castration-resistant prostate cancer (mCRPC) patients (pts) commencing enzalutamide (ENZ). European Journal of Cancer. 2015 Sep 1;51:S474–5.

110. Kohli M, Du M, Wang L, Huang CC. Abstract 4588: Prognostic association of plasma cell free DNA (cfDNA) copy number variation based algorithmic score with survival in metastatic castration resistant prostate cancer (mCRPC). Cancer Research. 2018 Jul 1;78(13_Supplement):4588.

111. Wise D, Kelvin J, Graf R, Schreiber NA, McLaughlin B, Fernandez L, et al. Glucocorticoid receptor (GR) expression in circulating tumor cells (CTCs) to prognosticate overall survival (OS) for metastatic castration-resistant prostate cancer (mCRPC) patients (pts) treated with androgen receptor signaling inhibitors (ARSi). JCO. 2017 Feb 20;35(6_suppl):194–194.

112. Conteduca V, Del Re M, Scarpi E, Crucitta S, Gurioli G, Restante G, et al. High exosomal PD-L1 expression in relation to lymph node progression in metastatic castration-resistant prostate cancer (mCRPC) treated with abiraterone (abi) or enzalutamide (enza). JCO. 2022 Jun;40(16_suppl):e17038–e17038.

113. Cho H, Cha J, Han KH, Chung JS. Abstract 3699: Identifying novel resistance biomarkers in circulating tumor cell-expressed transcriptomes of metastatic castration-resistant prostate cancer patients treated with androgen receptor signaling inhibitors. Cancer Research. 2024 Mar 22;84(6_Supplement):3699.

114. Gill DM, Agarwal N, Hahn AW, Johnson E, Poole A, Carroll E, et al. Impact of circulating tumor cell (CTC) nucleus size on outcomes with abiraterone acetate (AA) therapy in men with metastatic castration-resistant prostate cancer (mCRPC). JCO. 2017 Feb 20;35(6_suppl):253–253.

115. Morgan TM, Chung JS, Wang Y, Henderson J, Singhal U, Qiao Y, et al. Indentification of a CTC-based gene expression signature predicting resistance to abiraterone and enzalutamide in mCRPC. JCO. 2017 May 20;35(15_suppl):5072–5072.

116. Shiang A, Chauhan PS, Dang HX, Webster J, Ledet EM, Babbra RK, et al. Liquid biopsy AR/enhancer alteration detection before AR-targeted therapy and correlation with survival in metastatic castrate-resistant prostate cancer patients. JCO. 2022 Feb 20;40(6_suppl):171–171.

117. Isebia KT, de Jong A, de Weerd V, Beaufort C, Hamberg P, Lolkema MP, et al. mFAST-SeqS based aneuploidy score in circulating cell-free DNA and role as early response marker in patients with metastatic prostate cancer treated with androgen receptor signaling inhibitor. JCO. 2023 Jun;41(16_suppl):5058–5058.

118. Maruzzo M, Rossi E, Basso U, Facchinetti A, Anile G, Pierantoni F, et al. Prognostic and predictive role of CTCs and AR-V7+ CTCs expression in metastatic catrate resistant prostate cancer (mCRPC): A feasibility study. JCO. 2018 Feb 20;36(6_suppl):367–367.

119. Giridhar K, Sanhueza CT, Hillman DW, Alkhateeb H, Carlson R, Tan W, et al. Prognostic value of chromogranin-a (CGA) compared to circulating tumor cells (CTCs) in metastatic castration resistant prostate cancer (mCRPC). JCO. 2018 Feb 20;36(6_suppl):249–249.

120. Antonarakis ES, Zhang N, Saha J, Nevalaita L, Shell SA, Garratt C, et al. Real-world assessment of AR-LBD mutations in metastatic castration-resistant prostate cancer. JCO. 2023 Feb 20;41(6_suppl):204–204.

121. Giridhar K, Sosa C, Hillman DW, Sanhueza CT, Wang L, Cheville JC, et al. Whole blood androgen receptor (AR) variant (ARV12, ARV14) expression and overall survival (OS) in metastatic castrate resistant prostate cancer (mCRPC). JCO. 2017 May 20;35(15_suppl):5058–5058.

122. Chi KN, Annala M, Sunderland K, Khalaf D, Finch D, Oja CD, et al. A randomized phase II cross-over study of abiraterone + prednisone (ABI) vs enzalutamide (ENZ) for patients (pts) with metastatic, castration-resistant prostate cancer (mCRPC). JCO. 2017 May 20;35(15_suppl):5002–5002.

123. Khalaf D, Annala M, Finch DL, Oja CD, Vergidis J, Zulfiqar M, et al. Phase 2 randomized cross-over trial of abiraterone + prednisone (ABI+P) vs enzalutamide (ENZ) for patients (pts) with metastatic castration resistant prostate cancer (mCPRC): Results for 2nd-line therapy. JCO. 2018 May 20;36(15_suppl):5015–5015.
