# Supplementary material for: Liquid Biopsy Biomarkers in Metastatic Castration-Resistant Prostate Cancer Treated with Second-Generation Antiandrogens: Ready for Clinical Practice? A Systematic Review
Source: Cancers (Basel). 2025 Jul 27;17(15):2482. doi: 10.3390/cancers17152482 (PMC12345691; doi:10.3390/cancers17152482)
Supplement: Supplementary file 1 [file cancers-17-02482-s001.zip › Supplementary Data 3.pdf]

Table S3.1. AR SNVs as prognostic biomarkers for treatment with second-generation ARSI

Mutations	Article type	Source	Outcome	Treatment	Previous CRPC treatment	Population / Region	Prevalence of alteration	Significant in MV analysis	Follows checklist	Ref.
Pathogenic LBD mutations (L702H, T878A, F877L)	FT	cfDNA	PFS HR 2.10 (0.98–4.51)	ABI/ENZ	Prior taxanes allowed, no prior ARSI	Italy+United Kingdom	4.70%	Yes	Yes	(1)
Pathogenic LBD mutations	FT	cfDNA	TTP HR 1.02 (0.53–1.95)	ABI/ENZ	None (first-line)	Canada	6.90%	No	Yes	(2)
Pathogenic LBD mutations	FT	cfDNA	PFS HR 2.39 (1.11-5.14), OS HR 1.64 (0.56-4.82)	ABI/ENZ	Prior ChT or ARSI allowed	United States	12.90%	Only PFS	Yes	(3)
Pathogenic LBD mutations	Abs	cfDNA	Median OS 50.1 vs 60.7 mo. (p=0.013)	ABI/ENZ	First- or second-line ABI/ENZ	United States	21%	NR	Yes	(4)
AR mutations	Abs	cfDNA	crPFS HR 1.9 (p=0.11)	ENZ	Prior DTX and ABI allowed	Canada	22%	NR	No	(5)
AR mutations	FT	cfDNA	PFS HR 1.4 (0.73-2.8), OS HR 1.4 (0.71-2.9)	ABI/ENZ	Prior ARSI or ChT allowed	Australia	16.50%	NR	No	(6)
AR mutations	FT	cfDNA	PFS HR 1.523 (0.592-3.915)	ABI	Prior treatment allowed	China	8.60%	NR	No	(7)
AR mutations (>=2)	Abs	cfDNA	PFS HR 3.94 (1.46-10.64)	ENZ	NR	Canada	NR	NR	No	(8)

Note: Results are presented as hazard ratio (95% confidence interval) unless otherwise specified. Abbreviations: ABI, abiraterone; Abs, abstract; AR, androgen receptor; cfDNA, cell-free DNA; DTX, docetaxel; ENZ, enzalutamide; FT, full-text; HR, hazard ratio; LBD, ligand-binding domain; mo., months; MV, multivariable; NA, not applicable; NR, not reported; (c, r)PFS, (clinical, radiographic) progression-free survival; OS, overall survival; Ref., reference; TTP, time to progression

Table S3.2. Other AR-related biomarkers and their relation to outcomes under treatment with second-generation ARSI

Article type	AR-V	Source	Outcome	Treatment	Previous CRPC treatment	Population / Region	Prevalence of alteration	Significant in MV analysis	Follows checklist	Ref.
Abs	AR alterations	cfDNA	TTPP HR 2.04 (1.39 - 3.00)	ABI/ENZ	None (first line)	Canada	NR	No	Yes	(9)
FT	AR alterations	cfDNA	longer TTP (no p-value or CI)	ENZ	NR	United States	36%	NR	No	(10)

FT	AR alterations	cfDNA	PFS HR 2.47 (1.44–4.25),<br>OS HR 4.07 (1.85–8.95)	ABI/ENZ	Prior ARSI allowed	Japan	36%	No	Yes	(1<br>1)
FT	AR alterations	cfDNA and cfRNA	PSA PFS HR 2.8 (1.2-6.3),<br>crPFS HR 3.2 (1.2-8.3),<br>OS HR 3.1 (1.0-9.5)	ABI/ENZ	Prior ARSI or ChT allowed	Australia	53.70%	No	Yes	(1<br>2)
FT	AR alterations	cfDNA	PFS HR 1.9 (1.1-3.4),<br>OS HR 2.4 (1.3-4.4)	ABI/ENZ	Prior ARSI or ChT allowed	Australia	35.20%	PFS only	No	(6)
Abs	AR alterations	cfDNA	crPFS HR 3.5 (p<0.001)	ENZ	Prior DTX and ABI allowed	Canada	NR	NR	No	(5)
FT	AR-V7 by IHC	CTC	median PSA PFS ~170 days vs ~350 days (p=0.2976),<br>median cPFS ~165 days vs 160 days (p-0.5783)	ABI/ENZ	DTX or ARSI allowed	Germany	42.40%	NR	No	(1<br>3)
FT	Nuclear localized AR-V7	CTC	rPFS HR 3.7 (1.44-9.5),<br>median OS 4.6 mo. vs not reached (p<0.001)	ABI/ENZ/<br>APA	Unspecified first-line therapy	United States	12.5%	Yes	Yes	(1<br>4)
FT	Nuclear localized AR-V7	CTC	OS HR 0.62 (0.28 - 1.39)	ARSI	Unspecified first-line therapy	United States	23.90%	NR	Yes	(1<br>5)
FT	Nuclear localized AR-V7	CTC	median PFS 5.7 mo. (CI 3.8-7.0) vs 17.6 mo. (14.7-22.6)	ABI/ENZ/<br>APA	Prior ARSI allowed	United States	NR	Yes	Yes	(1<br>6)
FT	Nuclear localized AR-V7	CTC	PFS HR 10.39 (2.1-51.47)	ABI/ENZ/<br>APA	Prior ARSI/taxanes allowed	United States	NR	Yes	Yes	(1<br>7)
FT	AR splice variants	CTC	PFS HR 2.052 (1.013-4.160),<br>OS HR 2.093 (1.085-4.039)	ABI/ENZ	Prior ARSI or ChT allowed	Germany	48.30%	No	No	(1<br>8)
FT	1 AR splice variant (vs 2 or 3)	CTC	PFS HR 0.901 (0.363-2.234),<br>OS HR 0.528 (0.198-1.411)	ABI/ENZ	Prior ARSI or ChT allowed	Germany	20% of AR-V+ patients	No	No	(1<br>8)
FT	2 AR splice variants (vs 1 or 3)	CTC	PFS HR 0.998 (0.462-2.158),<br>OS HR 0.777 (0.368-1.641)	ABI/ENZ	Prior ARSI or ChT allowed	Germany	40% of AR-V+ patients	No	No	(1<br>8)
FT	3 AR splice variants (vs 1 or 2)	CTC	PFS HR 1.086 (0.496-2.380),<br>OS HR 2.063 (0.985-4.323)	ABI/ENZ	Prior ARSI or ChT allowed	Germany	40% of AR-V+ patients	No	No	(1<br>8)
FT	AR splice variants	CTC	time to no longer clinically benefiting HR 4.53 (1.424–14.41)	ABI	Prior ARSI allowed	Belgium	56.60%	NR	No	(1<br>9)
FT	AR splice variants	CTC	median PFS 3.9 vs. 10.0 mo. (p<0.0001)	ABI/ENZ	Prior ARSI or ChT allowed	Belgium	71.30%	No	Yes	(2<br>0)
Abs	AR-V7 or AR-V9	whole blood mRNA	median PSA PFS 9.2 mo. vs. not reached, p=0.894	ABI/ENZ	NR	Australia	24%	NR	No	(2<br>1)
FT	AR-V7 or AR-V9	cfDNA and cfRNA	PSA PFS HR 2.7 (1.0-7.0),<br>crPFS HR 3.0 (1.1-8.1),<br>OS HR 3.9 (1.3-12)	ABI/ENZ	Prior ARSI or ChT allowed	Australia	19.40%	No	Yes	(1<br>2)
Abs	AR-V12 or AR-V14	whole blood mRNA	OS HR 3.46 (p=0.006)	ABI	None (first-line)	United States	NR	NR	Yes	(2<br>2)
Abs	AR/enhancer alterations	cfDNA	PFS HR 2.21 (p=0.009),<br>OS HR 2.60 (p=0.02)	ARSI	None (first line)	United States	44%	NR	No	(2<br>3)
Abs	AR/enhancer alterations	cfDNA	PFS HR 2.1 (p = 0.046),<br>OS HR 1.05 (p = 0.19)	ABI/ENZ	No prior ARSI	United States	36%	NR	Yes	(2<br>4)

FT	AR LBD mutation or gain	cfDNA	PFS HR 2 (1.15-3.47), OS HR 2.95 (1.36-6.36)	ABI/ENZ	Prior ChT or ARSI allowed	United States	64.50%	No	Yes	(3)

Note: Results are presented as hazard ratio (95% confidence interval) unless otherwise specified. Abbreviations: ABI, abiraterone; Abs, abstract; AR, androgen receptor; AR-V, AR splice variant; cfDNA, cell-free DNA; cfRNA, cell-free RNA; CTC, circulating tumor cells; DTX, docetaxel; ENZ, enzalutamide; FT, full-text; HR, hazard ratio; LBD, ligand-binding domain; mo., months; MV, multivariable; NA, not applicable; NR, not reported; OS, overall survival; (c, r)PFS, (clinical, radiographic) progression-free survival; Ref., reference; TT(P)P, time to (PSA) progression.

Table S3.3. TP53 alterations as biomarkers of response to ARSI treatment

Article type	Source	Outcome	Treatment	Previous CRPC treatment	Population / Region	Prevalence of alteration	Significant in MV analysis	Follows checklist	Ref.
Abs	cfDNA	TTPP HR 2.84 (1.90 - 4.23)	ABI/ENZ	None (first-line)	Canada	NR	Yes	Yes	(9)
FT	cfDNA	>=1 alteration: TTP HR 2.70 (1.86–3.91), >=2 alterations: TTP HR 5.65 (3.14–10.17)	ABI/ENZ	None (first-line)	Canada	>=1 alteration: 32.7%; >=2 alterations: 9.4%	Yes	Yes	(2)
FT	cfDNA	PFS HR 2.55 (1.33–4.89), OS HR 4.05 (1.76–9.32)	ABI/ENZ	Prior ARSI allowed	Japan	18%	No	Yes	(11)
FT	cfDNA	PFS HR 1.33 (0.77-2.3), OS HR 3.19 (1.53-6.64)	ABI/ENZ	Prior ARSI or ChT allowed	United States	37.10%	OS only	Yes	(3)
FT	cfDNA	PFS HR 3.2 (1.6-6.4), OS HR 3.2 (1.6-6.4)	ABI/ENZ	Prior ARSI or ChT allowed	Australia	19.80%	NR	No	(6)
FT	cfDNA	rPFS HR 1.59 (1.004 - 2.51), OS HR 2.27 (1.38 - 3.72)	ABI	NR	Multicenter	24.2%	No	Yes	(25)
FT	cfDNA	median PFS 3.0 vs. 8.7 mo. (p<0.0001)	ABI/ENZ	Prior ARSI or ChT allowed	Belgium	24.80%	Yes	Yes	(20)

Note: Results are presented as hazard ratio (95% confidence interval) unless otherwise specified. Abbreviations: ABI, abiraterone; Abs, abstract; AR, androgen receptor; cfDNA, cell-free DNA; ENZ, enzalutamide; FT, full-text; HR, hazard ratio; mo., months; MV, multivariable; NA, not applicable; NR, not reported; OS, overall survival; (c, r)PFS, (clinical, radiographic) progression-free survival; Ref., reference; TT(P)P, time to (PSA) progression.

Table S3.4. DDR or HRR pathway alterations (other than TP53) and their relation to outcomes under ARSI treatment

Marker	Article type	Source	Outcome	Treatment	Previous CRPC treatment	Population / Region	Prevalence of alteration	Significant in MV analysis	Follows checklist	Ref.
BRCA2 or ATM alteration	Abs	cfDNA	TTPP HR 4.13 (2.55 - 6.68)	ABI/ENZ	None (first-line)	Canada	NR	Yes	Yes	(9)
BRCA2/ATM truncating mutation	FT	cfDNA	TTP HR 6.14 (3.35–11.26)	ABI/ENZ	None (first-line)	Canada	6.90%	Yes	Yes	(2)

BRCA2/ATM monoallelic deletion only	FT	cfDNA	TTP HR 2.58 (1.58–4.21)	ABI/ENZ	None (first-line)	Canada	10.90%	No	Yes	(2)
HRR pathway defect	Abs	CTC	PFS HR 2.42 (1.0-5.9)	ABI/ENZ	Prior ABI/ENZ allowed	United States	NR	NR	No	(26)
HRR pathway defect	FT	cfDNA	PFS HR 2.83 (1.40–5.73), OS HR 1.86 (0.76–4.59)	ABI/ENZ	Prior ARSI allowed	Japan	13%	NR	Yes	(11)
BRCA1/BRCA2/ATM mutation or loss	FT	cfDNA	PFS HR 0.95 (0.55-1.64), OS HR 1.45 (0.69-3.06)	ABI/ENZ	Prior ChT or ARSI allowed	United States	38.70%	No	Yes	(3)
BRCA1/BRCA2/ATM mutation	FT	cfDNA	PFS HR 0.89 (0.47-1.68), OS HR 1.03 (0.41-2.57)	ABI/ENZ	Prior ChT or ARSI allowed	United States	22.60%	No	Yes	(3)
DDR alteration	FT	cfDNA	rPFS HR 2.13 (1.30 - 3.48), OS HR 1.32 (0.78 - 2.30)	ABI	NR	Multicenter	17.2%	Yes	Yes	(25)

Note: Results are presented as hazard ratio (95% confidence interval) unless otherwise specified. Abbreviations: ABI, abiraterone; Abs, abstract; cfDNA, cell-free DNA; CTC, circulating tumor cells; DDR, DNA damage response pathway; ENZ, enzalutamide; FT, full-text; HR, hazard ratio; HRR, homologous recombination repair pathway; mo., months; MV, multivariable; NA, not applicable; NR, not reported; OS, overall survival; (c, r)PFS, (clinical, radiographic) progression-free survival; Ref., reference; TT(P)P, time to (PSA) progression.

Table S3.5. RB1 alterations and their relation to outcomes under ARSI treatment

Article type	Source	Outcome	Treatment	Previous CRPC treatment	Population / Region	Prevalence of alteration	Significant in MV analysis	Follows checklist	Ref.
Abs	cfDNA	TTPP HR 1.96 (1.28 - 3.00)	ABI/ENZ	None (first-line)	Canada	NR	No	Yes	(9)
FT	cfDNA	TTP HR 2.03 (1.36–3.04)	ABI/ENZ	None (first-line)	Canada	18.30%	No	Yes	(2)
FT	cfDNA	PFS HR 3.20 (1.63–6.29), OS HR 3.21 (1.40–7.36)	ABI/ENZ	Prior ARSI allowed	Japan	12%	No	Yes	(11)
FT	cfDNA	PFS HR 0.94 (0.52-1.7), OS HR 1.47 (0.68-3.18)	ABI/ENZ	Prior ARSI or ChT allowed	United States	27.40%	No	Yes	(3)
Abs	cfDNA	PFS HR 4.46 (2.28-8.74)	ENZ	NR	Canada	NR	NR	No	(8)
FT	cfDNA	OS HR 2.38 (1.37-4.17) (non-amplified vs amplified)	ABI	None (first-line)	United States	NR	Yes	Yes	(27)
FT	cfDNA	PFS HR 1.7 (0.92-3.2), OS HR 3.4 (1.8-6.5)	ABI/ENZ	Prior ARSI or ChT allowed	Australia	23.10%	NR	No	(6)
FT	cfDNA	rPFS HR 1.47 (0.90 - 2.41), OS HR 2.31 (1.34 - 4.0)	ABI	NR	Multicenter	18.0%	No	Yes	(25)

Note: Results are presented as hazard ratio (95% confidence interval) unless otherwise specified. Abbreviations: ABI, abiraterone; Abs, abstract; cfDNA, cell-free DNA; ChT, chemotherapy; ENZ, enzalutamide; FT, full-text; HR, hazard ratio; mo., months; MV, multivariable; NA, not applicable; NR, not reported; OS, overall survival; (c, r)PFS, (clinical, radiographic) progression-free survival; Ref., reference; TT(P)P, time to (PSA) progression.

Table S3.6. PTEN/PI3K pathway alterations and their relation to outcomes under ARSI treatment

Marker	Article type	Source	Outcome	Treatment	Previous CRPC treatment	Population / Region	Prevalence of alteration	Significant in MV analysis	Follows checklist	Ref.
PTEN negative	FT	CTC	rPFS HR 3.96 (1.8 - 8.72), OS HR 2.36 (1.12 - 5)	ENZ	Prior taxanes in most patients<br>Prior ABI in a minority	Italy	42.2%	No	Yes	(28)
PTEN negative	FT	CTC	rPFS HR 2.5 (1.25-5)	ABI/ENZ	Prior ABI/ENZ allowed	United States	20%	NR	Yes	(29)
PTEN not amplified vs amplified	FT	cfDNA	OS HR 2.04 (1.16-3.45) (not amplified vs amplified)	ABI	None (first-line)	United States	NR	No	Yes	(27)
PTEN alteration or loss	FT	cfDNA	PFS HR 1.3 (0.7-2.5), OS HR 2.1 (1.1-3.9)	ABI/ENZ	Prior ARSI or ChT allowed	Australia	29.70%	NR	No	(6)
PTEN alteration or loss	FT	cfDNA	cPFS HR 2.4 (1.3-4.6), OS HR 3.6 (1.7-7.3)	ABI/ENZ	Prior ARSI or ChT allowed	Australia	51.30%	Yes	Yes	(30)
PTEN alteration or loss	FT	cfDNA	rPFS HR 1.32 (0.78 - 2.30), OS HR 3.29 (1.77 -6.11)	ABI	NR	Multicenter	14.1%	Yes	Yes	(25)
PI3K pathway defect	FT	cfDNA	TTP HR 2.45 (1.71–3.51)	ABI/ENZ	None (first-line)	Canada	29.20%	No	Yes	(2)
PI3K pathway defect	FT	cfDNA	PFS HR 2.47 (1.23–4.97), OS HR 6.77 (2.91–15.8)	ABI/ENZ	Prior ARSI allowed	Japan	12%	No	Yes	(11)
PI3K pathway defect	FT	cfDNA	PFS HR 1.77 (0.97-3.22), OS HR 3.64 (1.69-7.86)	ABI/ENZ	Prior ARSI or ChT allowed	United States	24.20%	OS only	Yes	(3)
PIK3CA gain	FT	cfDNA	OS HR 2.39 (1.35-4.25)	ABI	None (first-line)	United States	NR	Yes	Yes	(27)
PIK3CA gain	FT	cfDNA	cPFS HR 2.8 (1.4-5.7), OS HR 4.6 (2.2-9.7)	ABI/ENZ	Prior ARSI or ChT allowed	Australia	17%	OS only	Yes	(30)
PIK3CA gain	FT	cfDNA	rPFS HR 1.16 (0.60 - 2.23), OS HR 2.07 (1.21 - 3.54)	ABI	NR	Multicenter	10.2%	No	Yes	(25)

Note: Results are presented as hazard ratio (95% confidence interval) unless otherwise specified. Abbreviations: ABI, abiraterone; Abs, abstract; cfDNA, cell-free DNA; ChT, chemotherapy; CTC, circulating tumor cells; ENZ, enzalutamide; FT, full-text; HR, hazard ratio; mo., months; MV,

multivariable; NA, not applicable; NR, not reported; PI3K, phosphoinositide 3-kinase; OS, overall survival; (c, r)PFS, (clinical, radiographic) progression-free survival; Ref., reference; TTP, time to progression.

Table S3.7. WNT pathway alterations and their relation to outcomes under ARSI treatment

Marker	Article type	Source	Outcome	Treatment	Previous CRPC treatment	Population / Region	Prevalence of alteration	Significant in MV analysis	Follows checklist	Ref.
WNT pathway defect	FT	cfDNA	TTP HR 1.29 (0.71–2.34)	ABI/ENZ	None (first-line)	Canada	7.90%	No	Yes	(2)
WNT pathway defect	FT	cfDNA	PFS HR 0.58 (0.18–1.89), OS HR 1.36 (0.41–4.51)	ABI/ENZ	Prior ARSI allowed	Japan	6%	NR	Yes	(11)
WNT pathway defect	FT	cfDNA	PFS HR 1.32 (0.64-2.73), OS HR 2.92 (1.28-6.68)	ABI/ENZ	Prior ARSI or ChT allowed	United States	14.50%	No	Yes	(3)
APC gain	FT	cfDNA	median OS ~17 mo. vs not reached (p=0.03)	ABI/ENZ	None (first-line)	Netherlands	50% (median expression used as threshold)	NR	No	(31)
WNT5B over-expression	FT	CTC	median PSA PFS ~65 vs ~165 days (p=0.002), median crPFS ~60 vs ~370 days (p=0.003)	ABI/ENZ	NR	United States	NR	NR	No	(32)
WNT5B over-expression	Abs	CTC	PSA PFS HR 5.4 (1.54-18.95), crPFS HR 4.78 (1.40-16.25)	ABI/ENZ	NR	United States	NR	NR	Yes	(33)

Note: Results are presented as hazard ratio (95% confidence interval) unless otherwise specified. Abbreviations: ABI, abiraterone; Abs, abstract; cfDNA, cell-free DNA; ChT, chemotherapy; CTC, circulating tumor cells; ENZ, enzalutamide; FT, full-text; HR, hazard ratio; NA, not applicable; NR, not reported; mo., months; MV, multivariable; OS, overall survival; (c, r)PFS, (clinical, radiographic) progression-free survival; Ref., reference; TTP, time to progression

Table S3.8. PSA, PSMA and PSCA and their relation to outcomes under ARSI treatment

Marker	Article type	Source	Outcome	Treatment	Previous CRPC treatment	Population / Region	Prevalence of alteration	Significant in MV analysis	Follows checklist	Ref.

KLK3	FT	whole blood mRNA	PSA PFS HR 1.2 (0.6 - 2.8), OS HR 1.4 (0.6 - 4.2)	ABI	Prior taxanes allowed	Canada	70.4%	NR	No	(34)
KLK3	FT	whole blood mRNA	TTF HR 1.84 (0.81–4.19) (multivariate)	ABI	Prior taxanes or ABI allowed	United States	56.80%	NA	Yes	(35)
KLK3	FT	whole blood mRNA	TTF HR 1.61 (0.63–4.17) (multivariate)	ENZ	Prior taxanes or ABI allowed	United States	62.70%	NA	Yes	(35)
KLK3	Abs	CTC	PSA PFS HR 7.12 (1.63-31.03), crPFS HR 6.37 (1.75-23.07)	ABI/ENZ	NR	United States	NR	NR	Yes	(33)
KLK3	FT	plasma RNA	PFS HR 3.16 (1.59-6.27)	ABI	Prior DTX allowed	Netherlands	32%	Yes	Yes	(36)
KLK3	FT	CTC	median PFS 16 mo. vs 13 mo. (p=0.56), median OS 29 mo. vs not reached (p=0.96) (relative to CTC+/KLK3-)	ABI/ENZ	None (first-line)	Italy	34.1%	NR	No	(37)
KLK3	FT	CTC	median PSA PFS ~60 vs ~145 days (p=0.002), median crPFS ~30 vs ~200 days (p=0.002)	ABI/ENZ	NR	United States	NR	NR	No	(32)
PSMA	FT	CTC	rPFS HR 2.8 (1.4–5.8), OS HR 3.4 (1.6–7.0) (relative to CTC-)	ABI/ENZ	Prior ABI/ENZ allowed	United States	17%	No	Yes	(38)
PSMA	Abs	CTC	PSA PFS HR 3.83 (1.62-9.03)	ABI/ENZ	NR	United States	NR	NR	Yes	(39)
PSMA	Abs	CTC	rPFS HR 4.75 (2.27-9.93), PSA PFS HR 2.28 (1.20-4.33), OS HR 6.99 (2.93-16.73)	ABI/ENZ	NR	South Korea	NR	NR	No	(40)
PSMA	FT	CTC	median PFS 16 mo. vs 13 mo. (p=0.92), median OS NR vs 29 mo. (p=0.37) (relative to CTC+/PSMA-)	ABI/ENZ	None (first-line)	Italy	31.8%	NR	No	(37)
PSCA	Abs	CTC	PSA PFS HR 4.35 (1.27-14.91), crPFS HR 5.43 (1.52-19.36)	ABI/ENZ	NR	United States	NR	NR	Yes	(33)
PSCA	FT	CTC	median PSA PFS ~65 vs ~230 days (p=0.001), median crPFS ~80 vs ~445 days (p=0.001)	ABI/ENZ	NR	United States	NR	NR	No	(32)

Note: Results are presented as hazard ratio (95% confidence interval) unless otherwise specified. Abbreviations: ABI, abiraterone; Abs, abstract; ChT, chemotherapy; CTC, circulating tumor cells; DTX, docetaxel; ENZ, enzalutamide; FT, full-text; HR, hazard ratio; mo., months; MV, multivariable; NA, not applicable; NR, not reported; OS, overall survival; (c, r)PFS, (clinical, radiographic) progression-free survival; Ref., reference; TTF, time to treatment failure.

References:

1. Conteduca V, Wetterskog D, Sharabiani MTA, Grande E, Fernandez-Perez MP, Jayaram A, et al. Androgen receptor gene status in plasma DNA associates with worse outcome on enzalutamide or abiraterone for castration-resistant prostate cancer: a multi-institution correlative biomarker study. Annals of Oncology. 2017 Jul 1;28(7):1508–16.

2. Annala M, Vandekerkhove G, Khalaf D, Taavitsainen S, Beja K, Warner EW, et al. Circulating Tumor DNA Genomics Correlate with Resistance to Abiraterone and Enzalutamide in Prostate Cancer. Cancer Discovery. 2018 Apr 1;8(4):444–57.

3. Torquato S, Pallavajjala A, Goldstein A, Valda Toro P, Silberstein JL, Lee J, et al. Genetic Alterations Detected in Cell-Free DNA Are Associated With Enzalutamide and Abiraterone Resistance in Castration-Resistant Prostate Cancer. JCO Precis Oncol. 2019 Apr;(3):1–14.

4. Antonarakis ES, Zhang N, Saha J, Nevalaita L, Shell SA, Garratt C, et al. Real-world assessment of AR-LBD mutations in metastatic castration-resistant prostate cancer. JCO. 2023 Feb 20;41(6_suppl):204–204.

5. Chi K, Azad A, Volik S, Haegert A, Zalcberg J, Bihan SL, et al. 2504 Genomic predictive and prognostic factors from plasma cell-free DNA (cfDNA) for metastatic castration-resistant prostate cancer (mCRPC) patients (pts) commencing enzalutamide (ENZ). European Journal of Cancer. 2015 Sep 1;51:S474–5.

6. Fettke H, Kwan EM, Bukczynska P, Steen JA, Docanto M, Ng N, et al. Independent prognostic impact of plasma NCOA2 alterations in metastatic castration-resistant prostate cancer. The Prostate. 2021;81(13):992–1001.

7. Dong B, Fan L, Yang B, Chen W, Li Y, Wu K, et al. Use of Circulating Tumor DNA for the Clinical Management of Metastatic Castration-Resistant Prostate Cancer: A Multicenter, Real-World Study. Journal of the National Comprehensive Cancer Network. 2021 May 14;19(8):905–14.

8. Azad A, Wyatt A, Volik, S, Gleave M, Collins ` Colin, Chi K. Genomic alterations in cell-free dna and enzalutamide resistance in castration-resistant prostate cancer. Asia-Pacific Journal of Clinical Oncology. 2016;12(S3):44–51.

9. Chi KN, Annala M, Sunderland K, Khalaf D, Finch D, Oja CD, et al. A randomized phase II cross-over study of abiraterone + prednisone (ABI) vs enzalutamide (ENZ) for patients (pts) with metastatic, castration-resistant prostate cancer (mCRPC). JCO. 2017 May 20;35(15_suppl):5002–5002.

10. Moses M, Niu A, Lilly MB, Hahn AW, Nussenzveig R, Ledet E, et al. Circulating-tumor DNA as predictor of enzalutamide response post-abiraterone treatment in metastatic castration-resistant prostate cancer. Cancer Treatment and Research Communications. 2020 Jan 1;24:100193.

11. Mizuno K, Sumiyoshi T, Okegawa T, Terada N, Ishitoya S, Miyazaki Y, et al. Clinical Impact of Detecting Low-Frequency Variants in Cell-Free DNA on Treatment of Castration-Resistant Prostate Cancer. Clinical Cancer Research. 2021 Nov 15;27(22):6164–73.

12. Fettke H, Kwan EM, Docanto MM, Bukczynska P, Ng N, Graham LJK, et al. Combined Cell-free DNA and RNA Profiling of the Androgen Receptor: Clinical Utility of a Novel Multianalyte

Liquid Biopsy Assay for Metastatic Prostate Cancer. European Urology. 2020 Aug 1;78(2):173–80.

13. Erb HHH, Sparwasser P, Diehl T, Hemmerlein-Thomas M, Tsaur I, Jüngel E, et al. AR-V7 Protein Expression in Circulating Tumour Cells Is Not Predictive of Treatment Response in mCRPC. Urologia Internationalis. 2020 Jan 17;104(3–4):253–62.

14. Scher HI, Lu D, Schreiber NA, Louw J, Graf RP, Vargas HA, et al. Association of AR-V7 on Circulating Tumor Cells as a Treatment-Specific Biomarker With Outcomes and Survival in Castration-Resistant Prostate Cancer. JAMA Oncology. 2016 Nov 1;2(11):1441–9.

15. Scher HI, Graf RP, Schreiber NA, Jayaram A, Winquist E, McLaughlin B, et al. Assessment of the Validity of Nuclear-Localized Androgen Receptor Splice Variant 7 in Circulating Tumor Cells as a Predictive Biomarker for Castration-Resistant Prostate Cancer. JAMA Oncology. 2018 Sep 1;4(9):1179–86.

16. Graf RP, Hullings M, Barnett ES, Carbone E, Dittamore R, Scher HI. Clinical Utility of the Nuclear-localized AR-V7 Biomarker in Circulating Tumor Cells in Improving Physician Treatment Choice in Castration-resistant Prostate Cancer. European Urology. 2020 Feb 1;77(2):170–7.

17. Scher HI, Graf RP, Schreiber NA, McLaughlin B, Lu D, Louw J, et al. Nuclear-specific AR-V7 Protein Localization is Necessary to Guide Treatment Selection in Metastatic Castration-resistant Prostate Cancer. European Urology. 2017 Jun 1;71(6):874–82.

18. Wüstmann N, Seitzer K, Humberg V, Vieler J, Grundmann N, Steinestel J, et al. Co-expression and clinical utility of AR-FL and AR splice variants AR-V3, AR-V7 and AR-V9 in prostate cancer. Biomarker Research. 2023 Apr 5;11(1):37.

19. De Laere B, van Dam PJ, Whitington T, Mayrhofer M, Diaz EH, Van den Eynden G, et al. Comprehensive Profiling of the Androgen Receptor in Liquid Biopsies from Castration-resistant Prostate Cancer Reveals Novel Intra-AR Structural Variation and Splice Variant Expression Patterns. European Urology. 2017 Aug 1;72(2):192–200.

20. De Laere B, Oeyen S, Mayrhofer M, Whitington T, van Dam PJ, Van Oyen P, et al. TP53 Outperforms Other Androgen Receptor Biomarkers to Predict Abiraterone or Enzalutamide Outcome in Metastatic Castration-Resistant Prostate Cancer. Clinical Cancer Research. 2019 Mar 15;25(6):1766–73.

21. To SQ, Kwan E, Fettke H, Mant A, Docanto M, Martelotto L, et al. Abstract 2593: AR-V7 and AR-V9 expression is not predictive of response to AR-axis targeting agents in metastatic castration-resistant prostate cancer. Cancer Research. 2018 Jul 1;78(13_Supplement):2593.

22. Giridhar K, Sosa C, Hillman DW, Sanhueza CT, Wang L, Cheville JC, et al. Whole blood androgen receptor (AR) variant (ARV12, ARV14) expression and overall survival (OS) in metastatic castrate resistant prostate cancer (mCRPC). JCO. 2017 May 20;35(15_suppl):5058–5058.

23. Alahi I, Chauhan PS, Shiang AL, Webster J, Dang HX, Greiner L, et al. Abstract 6698: Combinatorial genomic and epigenomic cell-free DNA analysis of high-risk metastatic castration resistant prostate cancer reveals prognostic liquid biopsy signatures. Cancer Research. 2023 Apr 4;83(7_Supplement):6698.

24. Shiang A, Chauhan PS, Dang HX, Webster J, Ledet EM, Babbra RK, et al. Liquid biopsy AR/enhancer alteration detection before AR-targeted therapy and correlation with survival in metastatic castrate-resistant prostate cancer patients. JCO. 2022 Feb 20;40(6_suppl):171–171.

25. Jayaram A, Wingate A, Wetterskog D, Wheeler G, Sternberg CN, Jones R, et al. Plasma tumor gene conversions after one cycle abiraterone acetate for metastatic castration-resistant prostate cancer: a biomarker analysis of a multicenter international trial. Annals of Oncology. 2021 Jun 1;32(6):726–35.

26. Zhang J, Zimmermann B, Galletti G, Halabi S, Gjyrezi A, Yang Q, et al. Association of circulating tumor cell RB1 loss RNA signature with outcomes and immune phenotypes in men with mCRPC. JCO. 2022 Feb 20;40(6_suppl):139–139.

27. Du M, Tian Y, Tan W, Wang L, Wang L, Kilari D, et al. Plasma cell-free DNA-based predictors of response to abiraterone acetate/prednisone and prognostic factors in metastatic castration-resistant prostate cancer. Prostate Cancer Prostatic Dis. 2020 Dec;23(4):705–13.

28. Lorenzo GD, Zappavigna S, Crocetto F, Giuliano M, Ribera D, Morra R, et al. Assessment of Total, PTEN–, and AR-V7+ Circulating Tumor Cell Count by Flow Cytometry in Patients with Metastatic Castration-Resistant Prostate Cancer Receiving Enzalutamide. Clinical Genitourinary Cancer. 2021 Oct 1;19(5):e286–98.

29. Gupta S, Hovelson DH, Kemeny G, Halabi S, Foo WC, Anand M, et al. Discordant and heterogeneous clinically relevant genomic alterations in circulating tumor cells vs plasma DNA from men with metastatic castration resistant prostate cancer. Genes, Chromosomes and Cancer. 2020;59(4):225–39.

30. Kwan EM, Dai C, Fettke H, Hauser C, Docanto MM, Bukczynska P, et al. Plasma Cell–Free DNA Profiling of PTEN-PI3K-AKT Pathway Aberrations in Metastatic Castration-Resistant Prostate Cancer. JCO Precis Oncol. 2021 Apr;(5):622–37.

31. Hendriks RJ, Dijkstra S, Smit FP, Vandersmissen J, Van de Voorde H, Mulders PFA, et al. Epigenetic markers in circulating cell-free DNA as prognostic markers for survival of castration-resistant prostate cancer patients. The Prostate. 2018;78(5):336–42.

32. Chung JS, Wang Y, Henderson J, Singhal U, Qiao Y, Zaslavsky AB, et al. Circulating Tumor Cell–Based Molecular Classifier for Predicting Resistance to Abiraterone and Enzalutamide in Metastatic Castration-Resistant Prostate Cancer. Neoplasia. 2019 Aug 1;21(8):802–9.

33. Morgan TM, Chung JS, Wang Y, Henderson J, Singhal U, Qiao Y, et al. Indentification of a CTC-based gene expression signature predicting resistance to abiraterone and enzalutamide in mCRPC. JCO. 2017 May 20;35(15_suppl):5072–5072.

34. Todenhöfer T, Azad A, Stewart C, Gao J, Eigl BJ, Gleave ME, et al. AR-V7 Transcripts in Whole Blood RNA of Patients with Metastatic Castration Resistant Prostate Cancer Correlate with Response to Abiraterone Acetate. The Journal of Urology [Internet]. 2017 Jan [cited 2025 Mar 6]; Available from: https://www.auajournals.org/doi/10.1016/j.juro.2016.06.094

35. Qu F, Xie W, Nakabayashi M, Zhang H, Jeong SH, Wang X, et al. Association of AR-V7 and Prostate-Specific Antigen RNA Levels in Blood with Efficacy of Abiraterone Acetate and Enzalutamide Treatment in Men with Prostate Cancer. Clinical Cancer Research. 2017 Jan 31;23(3):726–34.

36. Boerrigter E, Benoist GE, van Oort IM, Verhaegh GW, van Hooij O, Groen L, et al. Liquid biopsy reveals KLK3 mRNA as a prognostic marker for progression free survival in patients with metastatic castration-resistant prostate cancer undergoing first-line abiraterone acetate and prednisone treatment. Molecular Oncology. 2021;15(9):2453–65.

37. Francolini G, Loi M, Ciccone LP, Detti B, Di Cataldo V, Pinzani P, et al. Prospective assessment of AR splice variant and multi-biomarker expression on circulating tumor cells of mCRPC patients undergoing androgen receptor targeted agents: interim analysis of PRIMERA trial (NCT04188275). Med Oncol. 2022 Jun 10;39(8):119.

38. Gupta S, Halabi S, Yang Q, Roy A, Tubbs A, Gore Y, et al. PSMA-positive Circulating Tumor Cell Detection and Outcomes with Abiraterone or Enzalutamide Treatment in Men with Metastatic Castrate-resistant Prostate Cancer. Clinical Cancer Research. 2023 May 15;29(10):1929–37.

39. Chung JS, Wang Y, James H, Singhal U, Qiao Y, Zaslavsky A, et al. PD71-06 CTC-BASED GENE EXPRESSION FOR PREDICTING RESISTANCE TO ABIRATERONE AND ENZALUTAMIDE IN MCRPC. The Journal of Urology [Internet]. 2017 Apr [cited 2025 Mar 9]; Available from: https://www.auajournals.org/doi/10.1016/j.juro.2017.02.3173

40. Cho H, Cha J, Han KH, Chung JS. Abstract 3699: Identifying novel resistance biomarkers in circulating tumor cell-expressed transcriptomes of metastatic castration-resistant prostate cancer patients treated with androgen receptor signaling inhibitors. Cancer Research. 2024 Mar 22;84(6_Supplement):3699.
